# Alkali Metal Dihydropyridines in Transfer Hydrogenation Catalysis of Imines: Amide Basicity versus Hydride Surrogacy

**DOI:** 10.1002/anie.202304966

**Published:** 2023-05-22

**Authors:** Peter A. Macdonald, Sumanta Banerjee, Alan R. Kennedy, Alexander van Teijlingen, Stuart D. Robertson, Tell Tuttle, Robert E. Mulvey

**Affiliations:** ^1^ WestCHEM, Department of Pure and Applied Chemistry University of Strathclyde Glasgow G1 1XL UK

**Keywords:** Alkali Metal, Caesium, Catalysis, Dihydropyridine, Hydrogenation

## Abstract

Catalytic reduction of a representative set of imines, both aldimines and ketimines, to amines has been studied using transfer hydrogenation from 1,4‐dicyclohexadiene. Unusually, this has been achieved using s‐block pre‐catalysts, namely 1‐metallo‐2‐*tert*‐butyl‐1,2‐dihydropyridines, 2‐*t*BuC_5_H_5_NM, M(*t*BuDHP), where M=Li–Cs. Reactions have been monitored in C_6_D_6_ and tetrahydrofuran‐d_8_ (THF‐d_8_). A definite trend is observed in catalyst efficiency with the heavier alkali metal *t*BuDHPs outperforming the lighter congeners. In general, Cs(*t*BuDHP) is the optimal pre‐catalyst with, in the best cases, reactions producing quantitative yields of amines in minutes at room temperature using 5 mol % catalyst. Supporting the experimental study, Density Functional Theory (DFT) calculations have also been carried out which reveal that Cs has a pathway with a significantly lower rate determining step than the Li congener. In the postulated initiation pathways DHP can act as either a base or as a surrogate hydride.

## Graphical abstract

When it comes to catalytic transfer hydrogenation of imines to amines using 1,4‐cyclohexadiene as the hydrogen source, the 2‐*tert*‐butyl‐1,2‐dihydropyridyl complexes of the heavyweight alkali metals potassium, rubidium and caesium pack the greater power over their lightweight lithium and sodium rivals with, in best cases, near quantitative yields in minutes and reactions run at room temperature.

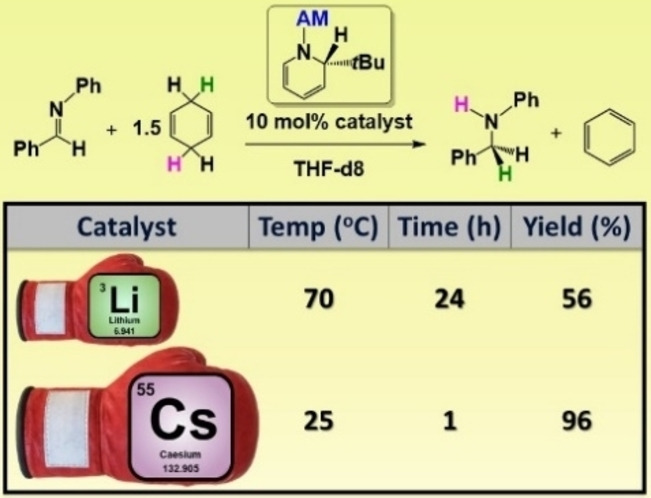

## Introduction

Organic amines are common synthons for the synthesis of many agrochemicals, fine chemicals, natural products, perfumes, and pharmaceuticals.^[1]^ An effective route for the synthesis of this valuable building block is reduction of imines by direct or transfer hydrogenation.^[1]^ An assortment of precious transition metal organometallic complexes are known to be effective catalysts for this important transformation.^[2]^ Despite the growing demand for sustainable catalysts,^[3]^ elements from the main group have received much less attention as alternatives for catalytic reduction, with the exception of a few notable precedents.[^3c^, ^4^] Thus, Stephan pioneered the use of Lewis acidic B(C_6_F_5_)_3_ in metal‐free hydrogenation of imines, exploiting the Frustrated Lewis Pair (FLP) mechanism,^[5]^ while soluble molecular aluminium^[6]^ and zinc^[5b]^ hydrides have been found to accomplish the same, albeit under harsh conditions (about 50–100 °C, 100 atm, mostly over 24 hours). Examples from the s‐block include Harder's use of Group 2 amides [M{N(SiMe_3_)_2_}_2_], where M=Mg, Ca, Sr, Ba,^[4b]^ and aluminates [M(AlH_4_)_2_ ⋅ (THF)_n_], where M=Mg, Ca, Sr,^[7]^ and two examples illustrating alkali metal mediation (*AMM*) using pre‐catalyst LiAlH_4_ for imine hydrogenation.[^3c^, ^4c^] Although direct hydrogenation is effective as an atom economical process, the drawbacks with respect to the handling and storage of H_2_ gas makes transfer hydrogenation an appealing alternative.^[8]^ To this end, promising but sporadic reports of both homogeneous and heterogeneous catalysis by alkali metal hydroxides, alkoxides, and phosphates in reduction of polar carbonyl functional groups using iso‐propanol as the transfer hydrogenating reagent have appeared.^[9]^ However, systematic studies of alkali metal based catalysts in transfer hydrogenation catalysis seem to be missing.

**Scheme 1:**
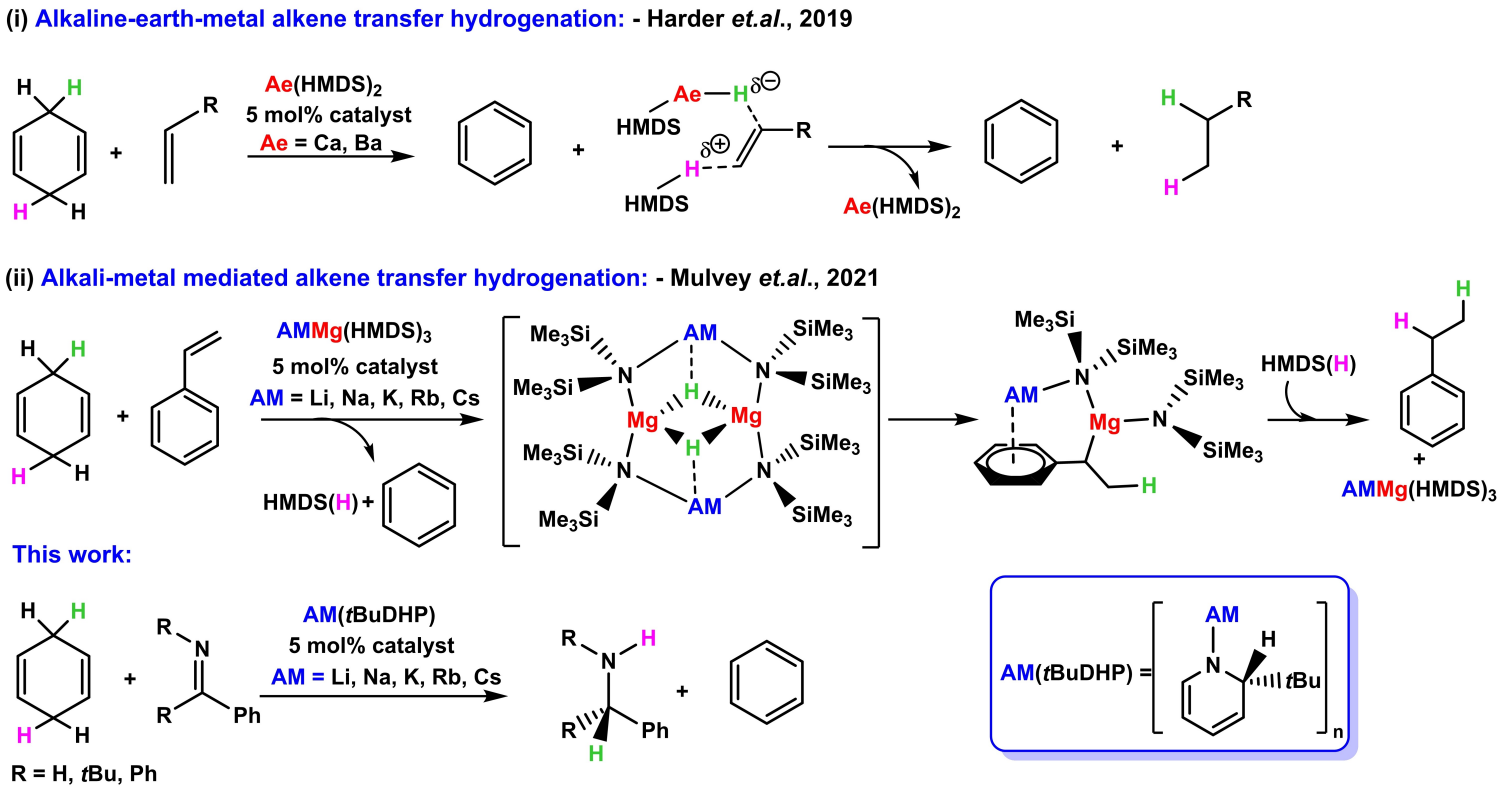
Transfer hydrogenation catalysis by s‐block catalysts using 1,4‐cyclohexadiene as the hydrogen source.

Harder demonstrated that 1,4‐cyclohexadiene (1,4‐CHD) can act as an effective hydrogen source by eliminating aromatic benzene in transfer hydrogenation of alkenes using alkaline earth metal amide catalysts [M{N(SiMe_3_)_2_}_2_] [Scheme 1(i)].^[10]^ Our group exploited this property of hydrogen transferability to study catalytic transfer hydrogenation of styrene and 1,1′‐diphenylethylene via a series of alkali‐metal magnesiates [AMMg{N(SiMe_3_)_2_}_3_]^[11]^ (AM=Li, Na, K, Rb, Cs) as catalysts, highlighting the necessity of *AMM* [(Scheme 1(ii)].[^11^, ^12^] It was proposed that reduction of alkenes by [M{N(SiMe_3_)_2_}_2_] is facilitated by forming the active heteroleptic Group 2 metal amide‐hydride species [M(H){N(SiMe_3_)_2_}]_n_ in situ via an unstable M—Meisenheimer intermediate that liberates benzene in a thermodynamically facile process.^[10]^ In the bimetallic scenario, a series of [AMMg(H){N(SiMe_3_)_2_}_2_]_2_ ⋅ (C_6_H_6_)_n_ inverse crown complexes (where AM=Na, K, Rb), thought to form by a similar deprotonation and hydride‐transfer mechanism, were isolated and characterised.^[11]^

From these findings, we envisioned that a soluble ‘AM−H’ species could be a potential candidate for transfer hydrogenation catalysis. Since our inception of [1‐lithio‐2‐*tert*‐butyl‐1,2‐dihydropyridine, 2‐*t*BuC_5_H_5_NLi, Li(*t*BuDHP)] as a surrogate source of soluble molecular ‘LiH’,^[13]^ its catalytic ability has been shown in homogeneous hydroboration of aldehydes and ketones, and in dehydrogenative cyclisation of diamine boranes.^[14]^ Aspiring to develop further use of heavier alkali metals in catalysis, we prepared the Na–Cs dihydropyridines by transmetallating the lithium congener with appropriate alkali metal alkoxides.^[15]^ These compounds of general formula [{AM(*t*BuDHP)}_n_, where AM=Li–Cs] remain stable for months when stored under inert conditions as powders in a freezer.

## Results and Discussion

Exploiting the completed set of donor‐free alkali‐metal‐dihydropyridines [AM(*t*BuDHP)], we screened them as catalysts for transfer hydrogenation using *N*‐benzylideneaniline [PhC(H)=NPh] as our substrate for optimizing reaction conditions. Its C=N bond had unexpectedly proved difficult to reduce using LiAlH_4_ ^[16]^ and Group 2 aluminates [e.g., Ca(AlH_4_)_2_ ⋅ (THF)_4_; Sr(AlH_4_)_2_] in direct hydrogenation.^[7]^ We chose C_6_D_6_ as the reaction medium along with 1.5 equivalents of 1,4‐CHD as hydrogen source. At room temperature using 10 mol % of AM(*t*BuDHP) (AM=Li, Na, K) no reaction occurred, but on raising the temperature to 70 °C for 24 hours, conversion was achieved showing a trend of increasing reactivity going down the group (21, 57, 85 % respectively, Table 1, entries 1–3). Testing the heaviest [Rb/Cs(*t*BuDHP)] at room temperature gave similar low activity as that of the lighter congeners due to their poor solubility; but yields (calculated using adamantane as standard) increased impressively to 92/97 % on heating to 70 °C (entries 4 and 5). Pleasingly, reaction times also decreased substantially compared to that of the lighter alkali metal congeners with Cs(*t*BuDHP) performing best among its peers, reaching 97 % yield in only 1 hour. Comparing this to a transition metal catalyst, Li et al., showed that the iridium anion in [Cp*Ir(2,2′‐bpyO)(OH)]^−^[Na]^+^ (bpy=2,2′‐bipyridine) could catalytically reduce this imine to 81 % yield in 12 hours, albeit using a different hydrogen source and a lower catalyst loading (methanol, 1 mol %).^[17]^ Adhering to the above conditions and decreasing the catalyst load to 5 mol %, did not affect the catalytic performance of [Cs(*t*BuDHP)] in the transfer hydrogenation of PhC(H)=NPh (96 %, entry 6). On changing the solvent to THF‐d_8_ yields increased to 56 % for [Li(*t*BuDHP)] (entry 7). For the heavier alkali metal catalysts (Rb, Cs), yields remained consistently high (>90 %) and reaction times shortened to 2.5 hours and 15 minutes respectively (entries 8 and 9). This acceleration could be explained by the enhanced solubility of the catalyst in this solvent. Significantly, it was also possible to complete this reaction using [Cs(*t*BuDHP)] in THF‐d_8_ without additional heating in 1 hour (entry 10). A 5 mol % catalytic loading did not lengthen reaction times, reaching a 96 % yield in 15 minutes at 70 °C (entry 11) with the Cs catalyst. Further reducing the catalyst loading to 2.5% expectedly led to longer reactions but conversion rates remained high (entry 12, 96 % 1.5 h, 70 °C). In light of these results the optimized conditions were set to 5 mol % of catalyst at 70 °C in THF‐d_8_ or C_6_D_6_.

**Table 1:**
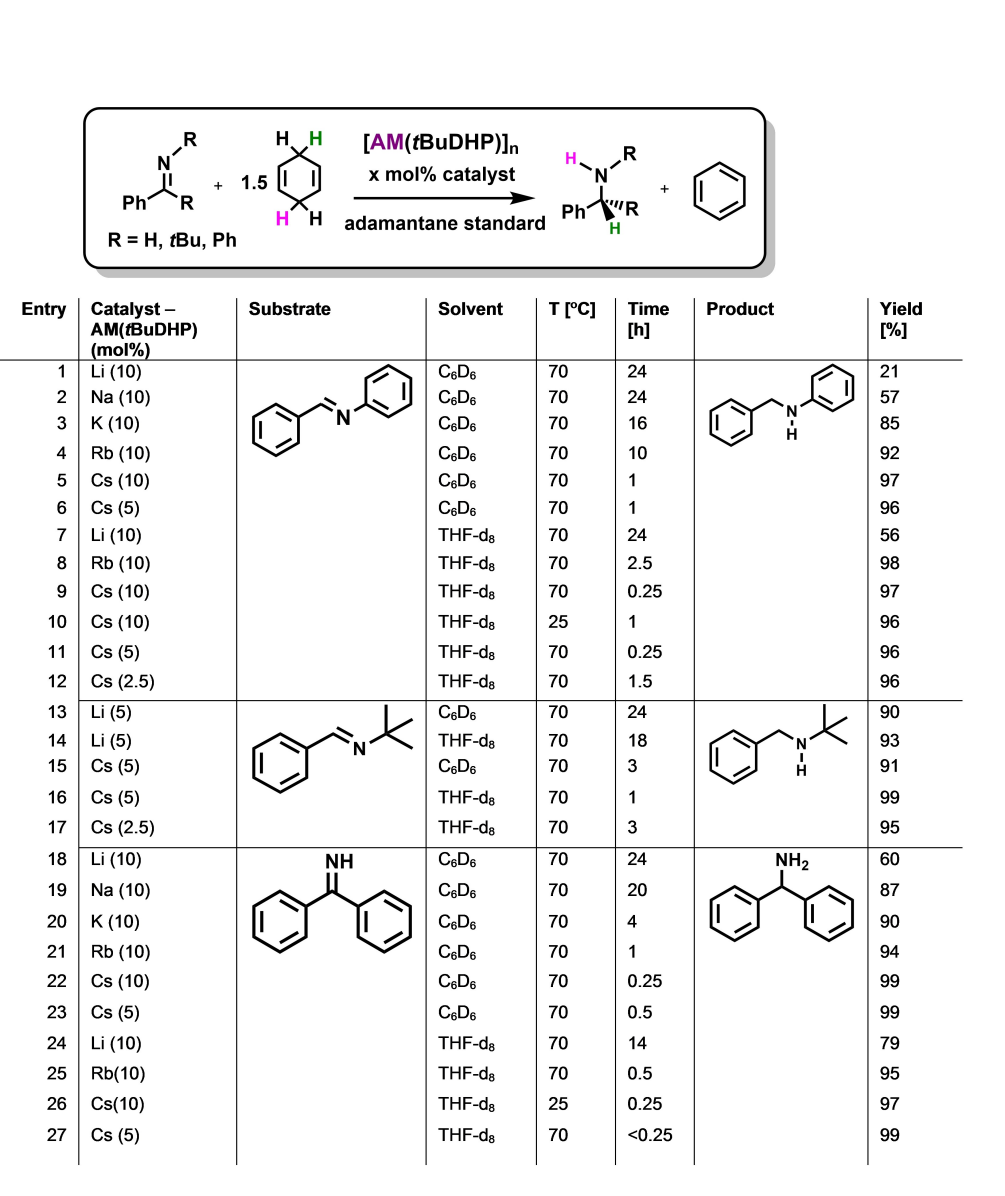
Catalytic transfer hydrogenation of imines using AM(*t*BuDHP).

For a comparison, we next turned to *N*‐benzylidene‐*tert*‐butylamine [PhC(H)=N*t*Bu], with its more polar C=N bond due to its electron donating *t*‐butyl group. This imine was probed using the best conditions optimised experimentally from Table 1. Both Li(*t*BuDHP) and Cs(*t*BuDHP) were tested catalytically to compare the two extremes of Group 1. Full conversion and nearly quantitative yields were found for Cs(*t*BuDHP) in C_6_D_6_ (91 % yield, 3 h, 70 °C, entry 15). In contrast, Li(*t*BuDHP) gave minimal conversion (15 %) after heating for 3 hours though conversions increased on heating for 24 hours, giving yields of 90 % compared to the 21 % seen for [PhC(H)=NPh] (entries 13 and 1 respectively). Changing the solvent to THF‐d_8_ again showed an improvement in yields and reaction times across both extremes, although to obtain a high yield (93 %), the Li(*t*BuDHP) catalysed reaction still required heating for 18 hours (entry 14). The reaction could be completed using the Cs(*t*BuDHP) catalyst with quantitative yields (99 %) in 1 hour at 5 mol % catalyst loading (entry 16), confirming that THF‐d_8_ is the most suitable solvent. Decreasing the catalytic loading further to 2.5 mol % followed the same trend of increased reaction times but yields still remain high (entry 17, 95 %, 3 h). In the absence of a metal, Soós^[18]^ showed for the first time that [PhC(H)=N*t*Bu] could be reduced by FLP chemistry with high conversion rates (99 %), although this required the use of pressurised H_2_ and long reaction times (42 h) in contrast to the performance in our study. Notably, Harder reported a diminishing reactivity with *N*‐benzylidene‐*tert*‐butylamine [PhC(H)=N*t*Bu] on going down the Group 1 aluminates ([LiAlH_4_]>[NaAlH_4_]>[KAlH_4_]).^[16]^ Solubility issues for the heavier AM systems is potentially a factor for the drop in reactivity, a problem partially thwarted in our *t*BuDHP systems where we see a reverse trend with the heavier metals being the leading performers.

Ketimines have generally been avoided or shown poor reactivity when tested for hydrogenation by main group metal catalysts.[^7^, ^19^] Thus, next we chose benzophenone imine [Ph_2_C=N(H)] as a more challenging test for the full AM(*t*BuDHP) series. Impressively, greater reactivity was observed across the whole series compared to that of the aldimines, again using C_6_D_6_ as the reaction medium with 10 mol % catalyst. Although the performances of the Li, Na and K (*t*BuDHP)’s were poorer than those of the heavier DHPs, their yields were significantly higher with shorter reaction times in this ketimine study (60–90 %, entries 18–20) compared to that of the aldimines (21–80 % yields). Rb(*t*BuDHP) was shown to yield 94 % of the target amine in 1 h (entry 21) although, Cs(*t*BuDHP) again gave the best results where an immediate colour change to dark red was noticed on heating after 1,4‐CHD addition. ^1^H NMR spectroscopic studies confirmed conversion to the corresponding amine (99 % yield) occurred in about 15 minutes at 70 °C at 10 % catalyst loading or 30 minutes at 5 % catalyst loading (entry 22 and 23). On changing the solvent to THF‐d_8_, Li(*t*BuDHP) and Rb(*t*BuDHP) provided shorter reaction times and better yields compared to using C_6_D_6_ as the reaction medium (79 % after 14 hours and 95 % after 30 minutes respectively, entries 24 and 25). Impressively, for Cs(*t*BuDHP) the reaction proceeded at room temperature over 15 minutes giving quantitative yields (entry 26, 97 %), while heating the solution decreased the reaction time to less than 15 minutes with no drop in yield even at 5 % catalyst loading (entry 27). Assessing how the Cs catalyst performs once the reaction had completed, another equivalent of imine and 1,4‐CHD was added (THF‐d_8_, 70 °C, 5 mol %, benzophenone imine), but the NMR yield and reaction time remained essentially the same. This addition was repeated three times with consistent results, before the reaction ceased (Figure S28, Supporting Information). To test how long the catalyst could survive in solution once a reaction (5 mol %, 70 °C, first cycle, *N*‐benzylidene‐*tert*‐butylamine) had completed, more imine and hydrogen source were added a week later. (Figure S29, Supporting Information). No decrease in reactivity was observed, prompting a study on the nature of the catalyst and the catalytic cycle.

**Figure 1:**
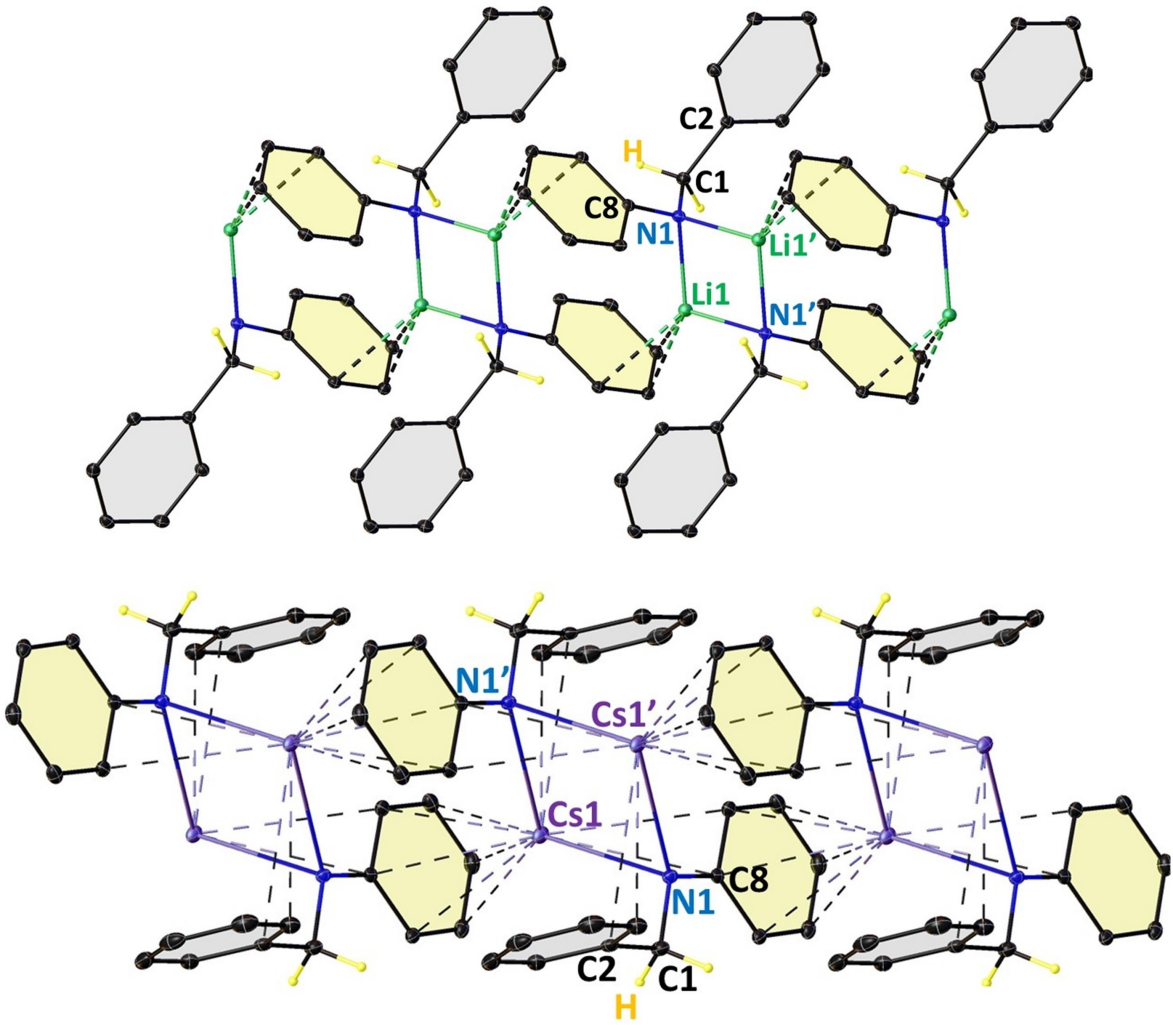
Polymeric structures of [PhCH_2_N(Li)Ph]_∞_, **1** (top), and [PhCH_2_N(Cs)Ph]_∞_, **2** (bottom).

From our earlier mechanistic studies on Li(*t*BuDHP) in ketone hydroboration, where the first catalytic step was insertion of the surrogate hydride into the polar carbonyl bond, we suspected a similar surrogate hydride reactivity to reduce the imine to the Cs‐amide with 2‐*tert*‐butylpyridine elimination. NMR scale stoichiometric reactions with Cs(*t*BuDHP) and PhC(H)=NPh established Cs‐amide [PhCH_2_N(Cs)Ph] was formed in THF‐d_8_, at 70 °C, in 45 mins. This was ascertained from the [PhC ***H***_2_N(Cs)Ph] resonance at 3.92 ppm and the 2‐*t*‐butylpyridine resonances in the aromatic region of the ^1^H NMR spectrum (see Figure S30, Supporting Information). A similar observation was made on mixing 1 : 1 quantities of Li(*t*BuDHP) with [PhC(H)=NPh] in C_6_D_6_, at 70 °C, for 12 hours (see Figure S31, Supporting Information). Pure Cs and Li amide products could also be isolated from 1 : 1 reaction of [(AM)N(SiMe_3_)_2_], AM=Li, Cs] with *N*‐benzylaniline [PhCH_2_N(H)Ph], with X‐ray quality crystals of potential reaction intermediates [PhCH_2_N(AM)Ph]_∞_, (AM=Li, **1**; Cs, **2**) grown from concentrated benzene solutions.^[20]^ XRD experiments revealed that both structures contain cyclodimeric [MN]_2_ central units, a common alkali‐metal amide structural motif,^[21]^ which polymerize via interactions between the alkali‐metal and the π‐system of a neighbouring N−Ph unit (Figure 1, see Table S1 for relevant bond parameters). The principal difference is in the amide geometry, as in the Cs case the benzyl group is twisted [C−N−C−C=177.30(16)°] so that it can provide additional π‐interactions to the larger alkali‐metal; whereas for smaller Li this interaction is not required [C−N−C−C=98.37(13)°]. To the best of our knowledge, these structures constitute the first such examples of alkali‐metal benzyl(phenyl)amide complexes, although we note lithium benzyl(*o*‐propenylphenyl)amide structures^[22]^ and lithium diphenylmethyl(phenyl)amide^[23]^ have been reported. Reacting 1,4‐CHD with the potential “Cs‐intermediate” **2**, gave the amine, *N*‐benzylaniline, in a 16 % conversion (24 hours at 70 °C in THF‐d_8_, Figure S34, Supporting Information) as determined by ^1^H NMR, showing that this could potentially be catalytically significant. However, these harsh conditions also caused the transformation of 1,4‐CHD to its 1,3‐CHD tautomer (60 %).

Probing the potential initiation step we reacted Cs(*t*BuDHP) with 1,4‐CHD in a 1 : 1 ratio in THF‐d_8_, at 70 °C for 1 hour, resulting in complete depletion of 1,4‐CHD resonances, with new resonances at 7.31 ppm and 4.55 ppm in the ^1^H NMR spectrum confirming benzene and a minute amount of H_2_ formation (see Figure S35, Supporting Information). Significantly, the five ^1^H NMR caesium dihydropyridyl [Cs(*t*BuDHP)] signals were retained, hinting towards a mechanism involving deprotonation of 1,4‐CHD by the amide [Cs(*t*BuDHP)] to form transient 2‐*t*‐butyldihydropyridine [2‐*t*‐Bu−C_5_H_5_NH] and the putative Cs‐Meisenheimer intermediate. This Cs‐Meisenheimer complex could eliminate benzene while the active ‘Cs−H’ formed in situ could deprotonate amine [2‐*t*‐Bu‐C_5_H_5_NH] to reform Cs(*t*BuDHP) and eliminate H_2_. Minute resonances of 1‐cyclohexene and 2‐*t*‐butylpyridine also in this spectrum indicates reduction of any excess 1,4‐CHD that may have been present due to experimental errors in measuring small amounts of reactants in a J. Young's NMR tube. Notably, while evidence of 2‐alkyl(phenyl)‐dihydropyridine can be found in previous literature studying decomposition and reaction pathways of 2‐alkyl(phenyl)‐1‐lithio‐1,2‐dihydropyridine, to the best of our knowledge no simple dihydropyridine (2‐R−C_5_H_5_NH) has been isolated.^[24]^

**Scheme 2:**
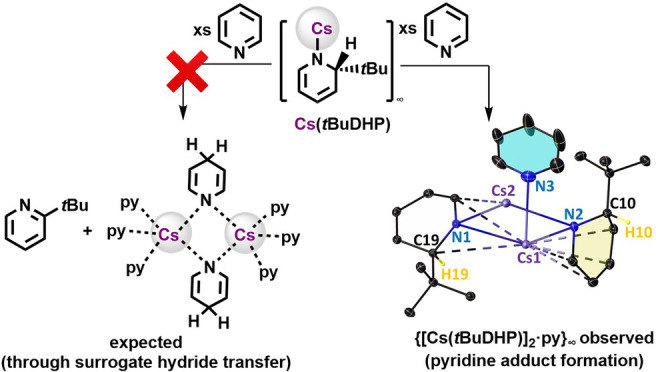
Pyridine adduct formation; asymmetric unit of {[Cs(*t*BuDHP)]_2_ ⋅ py}_∞_ (**3**) polymer depicted with thermal ellipsoids of all atoms displayed at 30 % probability. H atoms except the sp^3^ hybridized CH on the DHP ring have been removed for clarity.

To study the extent of (any) hydride donating ability of the surrogate hydrides in this system, we studied the reaction of Cs(*t*BuDHP) with pyridine, anticipating a pyridine‐stabilized 1,4‐dihydropyridine product, akin to the case with Li(***n*** **Bu**DHP) in excess pyridine (py), where bis(pyridine)1,4‐dihydropyridyllithium forms via a facile hydride transfer[^24a^, ^25^] (Scheme 2). Instead, we isolated crystals of the pyridine solvate {[Cs(***t*** **Bu**DHP)]_2_ ⋅ py}_∞_ (**3**), which crystallises as a polymer (see Figure S36/S37, Table S2, Supporting Information), hinting towards a decrease in surrogate hydride reactivity with respect to its *n*‐butyl congener. A similar experiment adding excess pyridine to donor free [Li(*t*BuDHP)] in hexane also yielded the monomeric tris‐pyridine solvate [Li(*t*BuDHP) ⋅ (py)_3_] (**4**, see Figure S38, Supporting Information).^[20]^ Isolation of **3** and **4** lends weight that the AM(*t*BuDHP)s do not necessarily behave as surrogate hydrides, with the hint above suggesting they could act as bases, where the balance of which may depend on the alkyl group located at the 2‐position on the DHP ring.

**Scheme 3:**
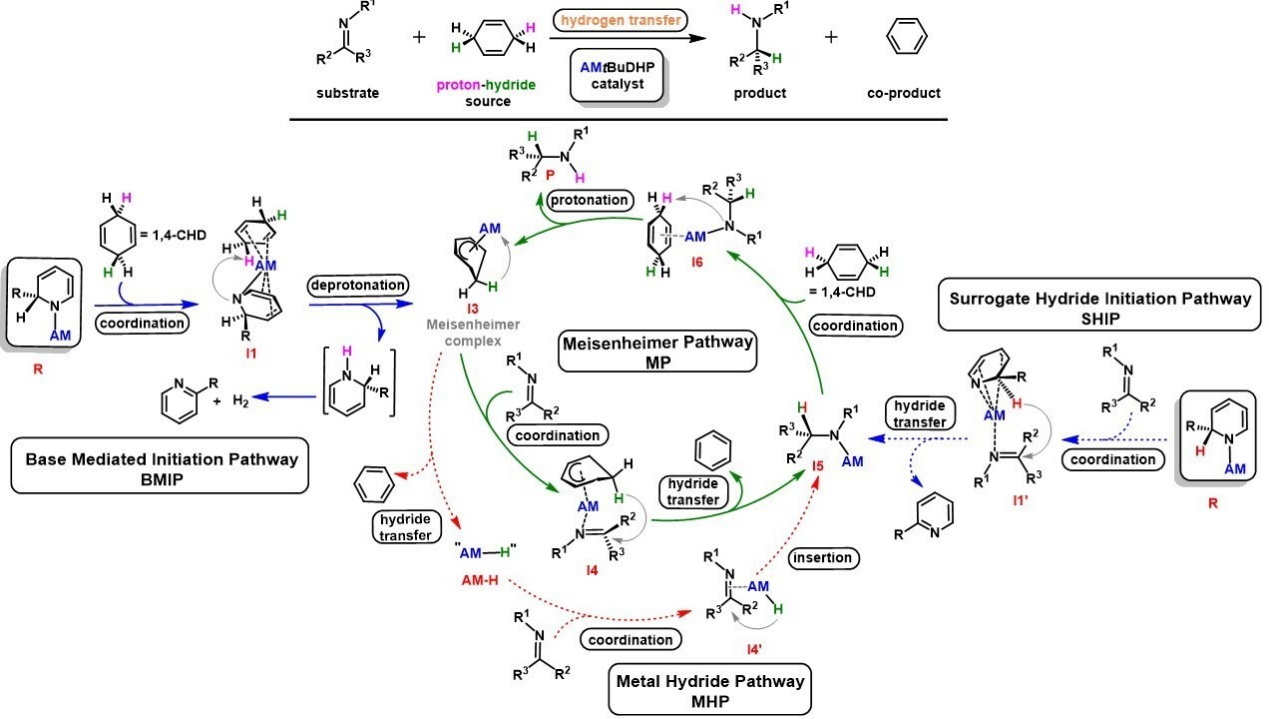
Proposed catalytic cycle for transfer hydrogenation of imines using AM(*t*BuDHP) where AM=Li–Cs. Blue arrows represent initiation pathways, with catalytic pathways distinguished by green and red arrows.

Using the above rationale, we propose a catalytic cycle (Scheme 3) initiated by Cs(*t*BuDHP) which acts as a base deprotonating 1,4‐CHD via coordination of the π‐rich diene system to the soft alkali metal (**I1**) to form a Cs‐Meisenheimer intermediate (**I3**) and [2‐*t*‐Bu−C_5_H_5_NH] (Base Mediated Initiation Pathway—BMIP). The Cs‐Meisenheimer complex can act in two alternative ways. It can eliminate benzene and the monomeric ‘Cs−H’ unit which is free to reduce the imine substrate to Cs‐amide (**I5**) (Metal Hydride Pathway—MHP). Alternatively, **I3** acts in a concerted fashion towards the imine (**I4**), to form the Cs‐amide intermediate (**I5**) via hydride transfer (Meisenheimer Pathway—MP). DFT calculations suggest the latter is more energetically feasible (see below). This newly formed metal amide can deprotonate 1,4‐CHD via intermediate **I6**, as experimentally verified (see above) thereby delivering the target product (**P**) and reforming the Cs—Meisenheimer complex (**I3**). From our experimental observations above, a contrasting initiation pathway (Surrogate Hydride Initiation Pathway—SHIP) can be postulated whereby the broken aromatic Cs(*t*BuDHP) pre‐catalyst acts as a surrogate hydride reducing the imine directly in a concerted manner to give Cs‐amide (**I5**) directly, thus accessing the same catalytic cycle as discussed above.

Supporting this reasoning, in a control reaction a 10 mol % sample of **2** (PhCH_2_N(Cs)Ph, equivalent to **I5** in Scheme 3) was dissolved in THF‐d_8_ and reacted with equimolar quantities of 1,4‐CHD and the imine (*N*‐benzylideneaniline) at 70 °C for 10 hours. This reaction was successful providing an NMR yield of 98 % of the target product *N*‐benzylaniline (see Supporting Information, Figure S39). However, PhCH_2_N(Cs)Ph is not as basic as Cs(*t*BuDHP) as evidenced from a further control reaction whereby PhCH_2_N(H)Ph was deprotonated by Cs(*t*BuDHP) (see Supporting Information, Figure S40).

The implication of BMIP is that this reaction could potentially be catalysed by other sufficiently basic caesium amides. To investigate this possibility we used Cs[N(SiMe_3_)_2_]^[26]^ as catalyst (10 mol %) for the transfer hydrogenation of *N*‐benzylideneaniline with 1,4‐CHD in THF‐d_8_. This reaction reached 98 % conversion after heating the mixture for 3 hours at 70 °C (see Supporting Information, Figure S41). Significantly, the same conversion can be reached using Cs(*t*BuDHP) as the catalyst in only 15 mins when performed under the same reaction conditions (Table 1, entry 9), suggesting that either Cs(*t*BuDHP) executes this initiation step quicker (presumably on account of being a stronger base) or this pre‐catalyst prefers the Surrogate Hydride Initiation Pathway which is not available to Cs[N(SiMe_3_)_2_].

Switching to the popular utility base Li[N(SiMe_3_)_2_], it proved to be a much inferior catalyst requiring 24 hours under identical conditions to reach only a 10 % conversion (see Supporting Information, Figure S42). This is in contrast with the results obtained with Li(*t*BuDHP) (Table 1, entry 7) which gave a five‐fold higher yield (56 %). Again, this can be explained by Li(*t*BuDHP) pre‐catalyst having access to both initiation pathways (BMIP and SHIP) as opposed to Li[N(SiMe_3_)_2_] which is limited to initiation via BMIP.

To investigate the potential competing mechanistic pathways, we initially optimised the geometries of the model structures with the aid of a deep neural network based on the ANI^[27]^ architecture and retrained to help find organopotassium transition states (see ESI—Deep neural network optimiser). Once located, all reported transition states and intermediates were calculated using the ORCA (version 5.0.3) software^[28]^ at the wB97X/def2‐TZVPP level of theory utilising the auxiliary basis set def2/J and D4 dispersion corrections.^[29]^ The free energies (G) for all species along the AM(*t*BuDHP) initiation pathways and catalytic cycles, where AM=Li or Cs (comparison for AM=K, is included in the Supporting Information, Figure S45, S46) were determined.

**Figure 2:**
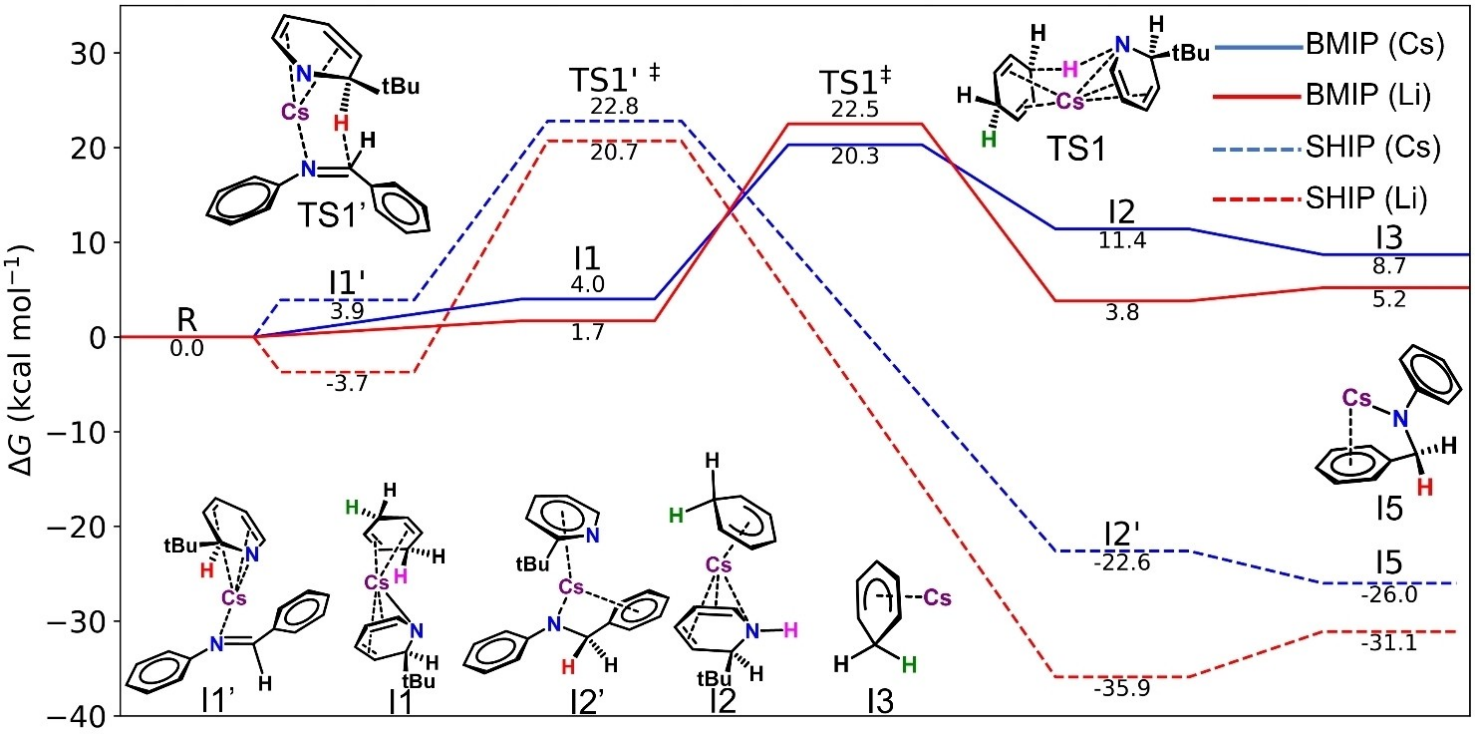
Gibbs free energy profile in kcal mol^−1^ for the catalytic initiation mechanisms, Solid lines represent Base Mediated Initiation Pathway (BMIP), Dashed lines represent Surrogate Hydride Initiation Pathway (SHIP) for caesium (blue) and lithium (red). All intermediates and transition states shown are for Cs catalysis. For corresponding Li intermediates and transition states, see Supporting Information Figure S43.

Analysing the initiation reactions (Figure 2) we find that both pathways have similar energy barriers (**TS1**/**TS1′**) with Cs(*t*BuDHP) marginally favouring BMIP (by 2.5 kcal mol^−1^), and Li(*t*BuDHP) following a similar trend (BMIP favoured by 1.9 kcal mol^−1^ over SHIP). However, for both metals, the stabilisation of **I5** is considerably greater than that of **I3**. The endergonic nature of the reaction to form **I3** indicates that the reaction is reversible, however, once **I3** is formed, the reverse reaction is limited due to the loss of H_2_ through evaporation. The necessity for H_2_ to be present to carry out the reaction from **I3** to **I1**, also explains why **I3** is not a decomposition pathway for the catalyst. Given the similarity in the activation barriers, both metals may allow access to the catalytic cycle through either the kinetically favoured **I3** intermediate, or the thermodynamically favoured **I5** intermediate. This supports the observation that our chosen dihydropyridyl complexes are superior catalysts to the other alkali‐metal secondary amides free of a surrogate hydride component that we have studied.

**Figure 3:**
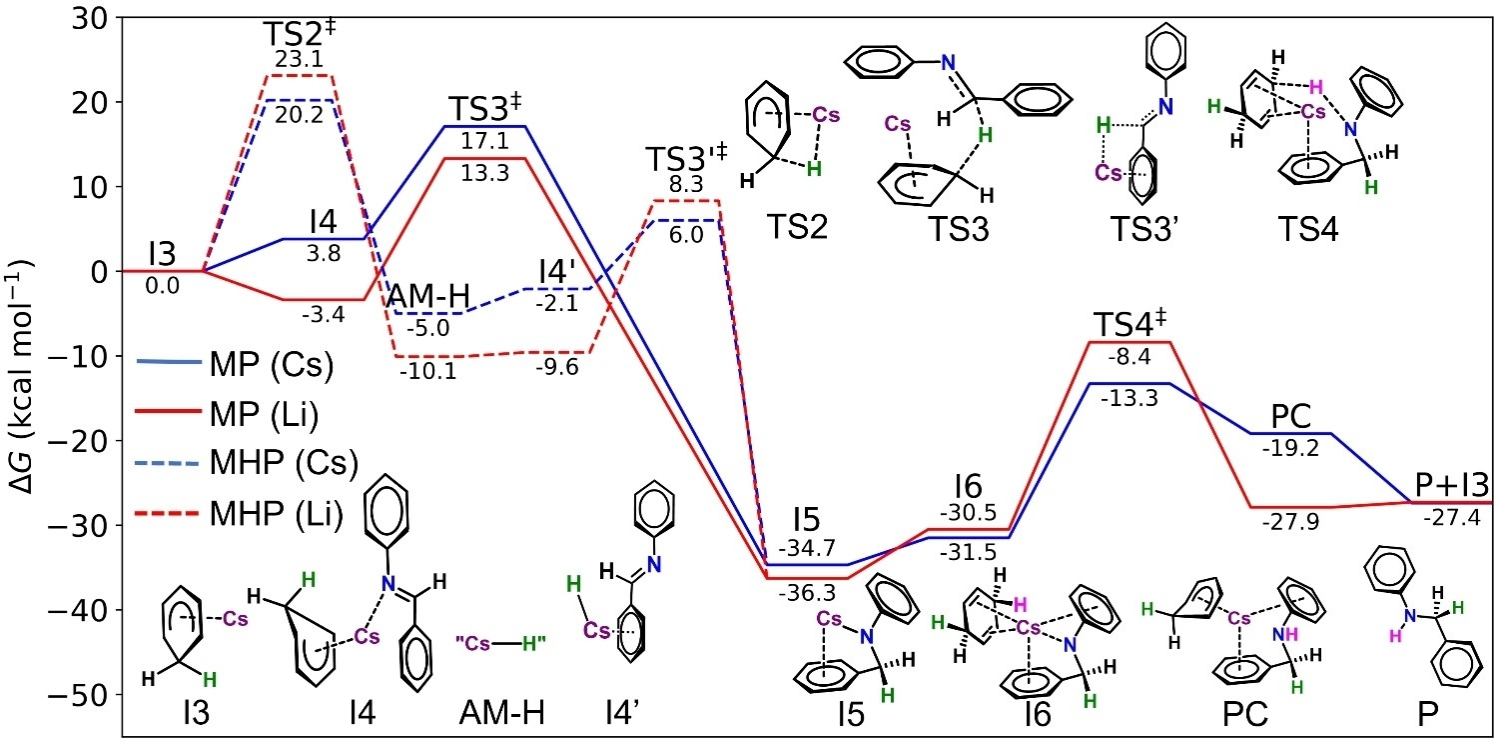
Gibbs free energy profile in kcal mol^−1^, relative to **I3**, for the catalytic cycle mechanisms, for caesium (blue) and lithium (red). The solid energy profile represents Meisenheimer Pathway (MP) and dashed energy profile represents the Metal Hydride Pathway (MHP). All intermediates and transition states shown are for Cs catalysis. For corresponding Li intermediates and transition states, see Supporting Information Figure S44.

The proposed pathways of the catalytic cycle, starting from the Meisenheimer complex **I3**, involve either conversion to alkali‐metal amide **I5** by concerted reduction of the imine via intermediate **I4** and **TS3** (Meisenheimer Pathway, MP, Figure 3) or elimination of benzene through hydride transfer via **TS2** to form metal hydride, **I4′** (Metal Hydride Pathway, MHP, Figure 3), which can then react directly with the imine to form **I5** (via **TS3′**) in a formally two‐step process. Our calculations favour the concerted MP over the two‐step MHP for both metals, with Cs and Li enjoying a 3.1 and 6.4 kcal mol^−1^ lower energy barrier respectively (i.e., **TS3** is lower than **TS2**). Overall, the conversion of **I3** to **I5** is strongly exergonic [Δ*G*(**I3**→**I5**)=−34.7/−36.3 kcal mol^−1^ for Cs/Li respectively, Figure 3]. As such, regardless of the induction pathway, both congeners are able to rapidly form the stable intermediate, **I5**, either directly (SHIP) or through the concerted MP as part of the catalytic cycle.

**I5** is able to complex with 1,4‐CHD to form **I6** and subsequently transfers a proton from the 1,4‐CHD to the substrate (via **TS4**, Figure 3) to reform **I3** and the product **P**. The barrier for this final proton transfer step in the case of Cs is 21.4 kcal mol^−1^ [Δ*G*(**I5**→**TS4**), Figure 3] and represents the rate limiting step in the Cs mediated cycle. In the case of Li, the pathway follows similar energetic trends but the activation barrier [Δ*G*(**I5**→**TS4**), Figure 3] is noticeably higher in comparison to that of Cs at 27.9 kcal mol^−1^, consistent with the superior performance of Cs catalysis versus Li catalysis.

## Conclusion

This study highlights the promise of caesium organometallic complexes as effective catalysts for transfer hydrogenation in the important conversion of imines to amines. It also establishes the versatility of alkali metal dihydropyridine (DHP) complexes in reactivity, here acting as Brønsted (C−H deprotonating) bases, where to date they have been mainly utilised as surrogate hydride sources in catalytic hydroboration, dehydrogenation and dehydrocoupling applications. Moreover, the major distinctions found here between Li(*t*BuDHP) and Cs(*t*BuDHP), emphasise that the alkali metals need to be treated as separate individuals not as a collective class when considering alkali‐metal‐mediated reactions. The development of caesium homogeneous catalysis is still at an embryonic stage as much work remains in for example applying it to other fundamentally important organic transformations, identifying the active catalysts/co‐catalysts involved especially where caesium has been introduced in salt form,^[30]^ unravelling other mechanistic pathways, and improving catalytic performance with respect to loading, temperature and reaction time etc. Future studies that reveal structurally well‐defined organocaesium complexes or the isolation and characterisation (in solution and solid phases) of caesium intermediates of organic substrates will be informative in progressing this development, at a minimum providing realistic starting structures for theoretical calculations probing possible catalytic cycles.

## Conflict of interest

The authors declare no conflict of interest.

1

## Supporting information

As a service to our authors and readers, this journal provides supporting information supplied by the authors. Such materials are peer reviewed and may be re‐organized for online delivery, but are not copy‐edited or typeset. Technical support issues arising from supporting information (other than missing files) should be addressed to the authors.

Supporting Information

Supporting Information

Supporting Information

## Data Availability

The data that support the findings of this study are openly available in Pureportal.strath.ac.uk at https://doi.org/10.15129/197577c8‐b28e‐43b4‐9ab4‐badd48c084f7, reference number 152719437.
