# Supplementary material for: Alkali Metal Dihydropyridines in Transfer Hydrogenation Catalysis of Imines: Amide Basicity versus Hydride Surrogacy
Source: Angew Chem Int Ed Engl. 2023 May 22;62(27):e202304966. doi: 10.1002/anie.202304966 (PMC10952797; doi:10.1002/anie.202304966)
Supplement: Supplementary file 1 — Supporting Information [file ANIE-62-0-s001.docx]

**checkCIF (basic structural check) running**

*Checking for embedded fcf data in CIF ...*
*Found embedded fcf data in CIF. Extracting fcf data from uploaded CIF, please wait***. . . . . . . . . .**

**checkCIF/PLATON (basic structural check)**

Structure factors have been supplied for datablock(s) csbenzintermediate23, csdhp_recol_smart, csdhppy, lidhp-xspyridine_auto, liytunknown_auto, rbdhp-recol_smart

THIS REPORT IS FOR GUIDANCE ONLY. IF USED AS PART OF A REVIEW PROCEDURE FOR PUBLICATION, IT SHOULD NOT REPLACE THE EXPERTISE OF AN EXPERIENCED CRYSTALLOGRAPHIC REFEREE.

No syntax errors found. [CIF dictionary](https://www.iucr.org/iucr-top/cif/cif_core/definitions/index.html)
Please wait while processing .... [Interpreting this report](https://journals.iucr.org/services/cif/checking/checkcifreport.html)

[Structure factor report](https://checkcif.iucr.org/ODxnpQbsZBM39/102422124910982811940/ckf.html)

**Datablock: LiYTunknown_auto**

| Bond precision: | C-C = 0.0018 A | Wavelength=1.54184 |
| --- | --- | --- |

| Cell: | a=5.6553(1) | b=12.0113(2) | c=14.7587(2) |
| --- | --- | --- | --- |
|  | alpha=90 | beta=94.540(2) | gamma=90 |
| Temperature: | 100 K |  |  |

|  | Calculated | Reported |
| --- | --- | --- |
| Volume | 999.38(3) | 999.38(3) |
| Space group | P 21/c | P 1 21/c 1 |
| Hall group | -P 2ybc | -P 2ybc |
| Moiety formula | C13 H12 Li N | C13 H12 Li N |
| Sum formula | C13 H12 Li N | C13 H12 Li N |
| Mr | 189.18 | 189.18 |
| Dx,g cm-3 | 1.257 | 1.257 |
| Z | 4 | 4 |
| Mu (mm-1) | 0.542 | 0.542 |
| F000 | 400.0 | 400.0 |
| F000' | 401.00 |  |
| h,k,lmax | 7,14,18 | 7,14,18 |
| Nref | 1987 | 1981 |
| Tmin,Tmax | 0.855,0.917 | 0.299,1.000 |
| Tmin' | 0.850 |  |

| Correction method= # Reported T Limits: Tmin=0.299 Tmax=1.000 AbsCorr = MULTI-SCAN |  |
| --- | --- |

| Data completeness= 0.997 | Theta(max)= 73.102 |
| --- | --- |

| R(reflections)= 0.0429( 1890) | wR2(reflections)= 0.1116( 1981) |
| --- | --- |
| \| S = 1.087 \| Npar= 184 \| \| --- \| --- \| |  |

The following ALERTS were generated. Each ALERT has the format

**test-name_ALERT_alert-type_alert-level**.

Click on the hyperlinks for more details of the test.


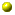
**Alert level C**

[PLAT906_ALERT_3_C](javascript:makeHelpWindow(%22PLAT906.html%22)) Large K Value in the Analysis of Variance ...... 3.414 Check


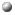
**Alert level G**

[PLAT004_ALERT_5_G](javascript:makeHelpWindow(%22PLAT004.html%22)) Polymeric Structure Found with Maximum Dimension 1 Info

[PLAT343_ALERT_2_G](javascript:makeHelpWindow(%22PLAT343.html%22)) Unusual Angle Range in Main Residue for C12 Check

[PLAT764_ALERT_4_G](javascript:makeHelpWindow(%22PLAT764.html%22)) Overcomplete CIF Bond List Detected (Rep/Expd) . 1.44 Ratio

[PLAT779_ALERT_4_G](javascript:makeHelpWindow(%22PLAT779.html%22)) Suspect or Irrelevant (Bond) Angle(s) in CIF ... 43.65 Deg.

N1 -C8 -LI1 1_555 1_555 1_555 ...... # 52 Check

[PLAT912_ALERT_4_G](javascript:makeHelpWindow(%22PLAT912.html%22)) Missing # of FCF Reflections Above STh/L= 0.600 6 Note

[PLAT978_ALERT_2_G](javascript:makeHelpWindow(%22PLAT978.html%22)) Number C-C Bonds with Positive Residual Density. 9 Info

0 **ALERT level A** = Most likely a serious problem - resolve or explain

0 **ALERT level B** = A potentially serious problem, consider carefully

1 **ALERT level C** = Check. Ensure it is not caused by an omission or oversight

6 **ALERT level G** = General information/check it is not something unexpected

0 ALERT type 1 CIF construction/syntax error, inconsistent or missing data

2 ALERT type 2 Indicator that the structure model may be wrong or deficient

1 ALERT type 3 Indicator that the structure quality may be low

3 ALERT type 4 Improvement, methodology, query or suggestion

1 ALERT type 5 Informative message, check

**Datablock: Lidhp-xspyridine_auto**

| Bond precision: | C-C = 0.0022 A | Wavelength=1.54184 |
| --- | --- | --- |

| Cell: | a=9.3266(2) | b=9.7255(3) | c=13.0828(3) |
| --- | --- | --- | --- |
|  | alpha=76.604(2) | beta=84.0415(19) | gamma=86.188(2) |
| Temperature: | 100 K |  |  |

|  | Calculated | Reported |
| --- | --- | --- |
| Volume | 1147.06(5) | 1147.07(5) |
| Space group | P -1 | P -1 |
| Hall group | -P 1 | -P 1 |
| Moiety formula | C24 H29 Li N4 | C24 H29 Li N4 |
| Sum formula | C24 H29 Li N4 | C24 H29 Li N4 |
| Mr | 380.45 | 380.45 |
| Dx,g cm-3 | 1.102 | 1.102 |
| Z | 2 | 2 |
| Mu (mm-1) | 0.501 | 0.501 |
| F000 | 408.0 | 408.0 |
| F000' | 409.03 |  |
| h,k,lmax | 11,12,16 | 11,12,16 |
| Nref | 4591 | 4573 |
| Tmin,Tmax | 0.905,0.905 | 0.523,1.000 |
| Tmin' | 0.905 |  |

| Correction method= # Reported T Limits: Tmin=0.523 Tmax=1.000 AbsCorr = MULTI-SCAN |  |
| --- | --- |

| Data completeness= 0.996 | Theta(max)= 73.271 |
| --- | --- |

| R(reflections)= 0.0476( 4153) | wR2(reflections)= 0.1300( 4573) |
| --- | --- |
| \| S = 1.047 \| Npar= 285 \| \| --- \| --- \| |  |

The following ALERTS were generated. Each ALERT has the format

**test-name_ALERT_alert-type_alert-level**.

Click on the hyperlinks for more details of the test.


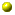
**Alert level C**

[PLAT230_ALERT_2_C](javascript:makeHelpWindow(%22PLAT230.html%22)) Hirshfeld Test Diff for C1 --C4 . 6.5 s.u.


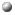
**Alert level G**

[PLAT793_ALERT_4_G](javascript:makeHelpWindow(%22PLAT793.html%22)) Model has Chirality at C5 (Centro SPGR) R Verify

[PLAT912_ALERT_4_G](javascript:makeHelpWindow(%22PLAT912.html%22)) Missing # of FCF Reflections Above STh/L= 0.600 19 Note

[PLAT978_ALERT_2_G](javascript:makeHelpWindow(%22PLAT978.html%22)) Number C-C Bonds with Positive Residual Density. 3 Info

[PLAT992_ALERT_5_G](javascript:makeHelpWindow(%22PLAT992.html%22)) Repd & Actual _reflns_number_gt Values Differ by 3 Check

0 **ALERT level A** = Most likely a serious problem - resolve or explain

0 **ALERT level B** = A potentially serious problem, consider carefully

1 **ALERT level C** = Check. Ensure it is not caused by an omission or oversight

4 **ALERT level G** = General information/check it is not something unexpected

0 ALERT type 1 CIF construction/syntax error, inconsistent or missing data

2 ALERT type 2 Indicator that the structure model may be wrong or deficient

0 ALERT type 3 Indicator that the structure quality may be low

2 ALERT type 4 Improvement, methodology, query or suggestion

1 ALERT type 5 Informative message, check

**Datablock: CsBenzIntermediate23**

| Bond precision: | C-C = 0.0032 A | Wavelength=0.71073 |
| --- | --- | --- |

| Cell: | a=19.0206(3) | b=6.6473(1) | c=20.1483(3) |
| --- | --- | --- | --- |
|  | alpha=90 | beta=109.236(2) | gamma=90 |
| Temperature: | 153 K |  |  |

|  | Calculated | Reported |
| --- | --- | --- |
| Volume | 2405.24(7) | 2405.24(7) |
| Space group | C 2/c | C 1 2/c 1 |
| Hall group | -C 2yc | -C 2yc |
| Moiety formula | C13 H12 Cs N | C13 H12 Cs N |
| Sum formula | C13 H12 Cs N | C13 H12 Cs N |
| Mr | 315.15 | 315.15 |
| Dx,g cm-3 | 1.741 | 1.741 |
| Z | 8 | 8 |
| Mu (mm-1) | 3.046 | 3.046 |
| F000 | 1216.0 | 1216.0 |
| F000' | 1213.34 |  |
| h,k,lmax | 26,9,27 | 25,9,27 |
| Nref | 3299 | 3067 |
| Tmin,Tmax | 0.501,0.614 | 0.692,1.000 |
| Tmin' | 0.491 |  |

| Correction method= # Reported T Limits: Tmin=0.692 Tmax=1.000 AbsCorr = MULTI-SCAN |  |
| --- | --- |

| Data completeness= 0.930 | Theta(max)= 29.369 |
| --- | --- |

| R(reflections)= 0.0199( 2770) | wR2(reflections)= 0.0431( 3067) |
| --- | --- |
| \| S = 1.055 \| Npar= 136 \| \| --- \| --- \| |  |

The following ALERTS were generated. Each ALERT has the format

**test-name_ALERT_alert-type_alert-level**.

Click on the hyperlinks for more details of the test.


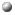
**Alert level G**

[PLAT004_ALERT_5_G](javascript:makeHelpWindow(%22PLAT004.html%22)) Polymeric Structure Found with Maximum Dimension 1 Info

[PLAT343_ALERT_2_G](javascript:makeHelpWindow(%22PLAT343.html%22)) Unusual Angle Range in Main Residue for C8 Check

[PLAT343_ALERT_2_G](javascript:makeHelpWindow(%22PLAT343.html%22)) Unusual Angle Range in Main Residue for C9 Check

[PLAT764_ALERT_4_G](javascript:makeHelpWindow(%22PLAT764.html%22)) Overcomplete CIF Bond List Detected (Rep/Expd) . 1.80 Ratio

[PLAT910_ALERT_3_G](javascript:makeHelpWindow(%22PLAT910.html%22)) Missing # of FCF Reflection(s) Below Theta(Min). 3 Note

[PLAT912_ALERT_4_G](javascript:makeHelpWindow(%22PLAT912.html%22)) Missing # of FCF Reflections Above STh/L= 0.600 226 Note

[PLAT978_ALERT_2_G](javascript:makeHelpWindow(%22PLAT978.html%22)) Number C-C Bonds with Positive Residual Density. 9 Info

0 **ALERT level A** = Most likely a serious problem - resolve or explain

0 **ALERT level B** = A potentially serious problem, consider carefully

0 **ALERT level C** = Check. Ensure it is not caused by an omission or oversight

7 **ALERT level G** = General information/check it is not something unexpected

0 ALERT type 1 CIF construction/syntax error, inconsistent or missing data

3 ALERT type 2 Indicator that the structure model may be wrong or deficient

1 ALERT type 3 Indicator that the structure quality may be low

2 ALERT type 4 Improvement, methodology, query or suggestion

1 ALERT type 5 Informative message, check

**Datablock: CsDHP.py**

| Bond precision: | C-C = 0.0083 A | Wavelength=1.54184 |
| --- | --- | --- |

| Cell: | a=13.9043(1) | b=10.0549(1) | c=20.7075(2) |
| --- | --- | --- | --- |
|  | alpha=90 | beta=105.309(1) | gamma=90 |
| Temperature: | 100 K |  |  |

|  | Calculated | Reported |
| --- | --- | --- |
| Volume | 2792.31(5) | 2792.31(5) |
| Space group | P 21/c | P 21/c |
| Hall group | -P 2ybc | -P 2ybc |
| Moiety formula | 4(C23 H33 Cs2 N3), C5 H5 N | ? |
| Sum formula | C97 H137 Cs8 N13 | C24.25 H34.25 Cs2 N3.25 |
| Mr | 2548.48 | 637.12 |
| Dx,g cm-3 | 1.516 | 1.516 |
| Z | 1 | 4 |
| Mu (mm-1) | 20.451 | 20.451 |
| F000 | 1250.0 | 1250.0 |
| F000' | 1247.60 |  |
| h,k,lmax | 17,12,25 | 17,12,25 |
| Nref | 5619 | 5607 |
| Tmin,Tmax | 0.129,0.441 | 0.143,1.000 |
| Tmin' | 0.011 |  |

| Correction method= # Reported T Limits: Tmin=0.143 Tmax=1.000 AbsCorr = MULTI-SCAN |  |
| --- | --- |

| Data completeness= 0.998 | Theta(max)= 73.287 |
| --- | --- |

| R(reflections)= 0.0392( 5356) | wR2(reflections)= 0.1109( 5607) |
| --- | --- |
| \| S = 1.113 \| Npar= 347 \| \| --- \| --- \| |  |

The following ALERTS were generated. Each ALERT has the format

**test-name_ALERT_alert-type_alert-level**.

Click on the hyperlinks for more details of the test.


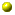
**Alert level C**

[CRYSC01_ALERT_1_C](javascript:makeHelpWindow(%22CRYSC_01.html%22)) The word below has not been recognised as a standard

identifier.

brownish

[CRYSC01_ALERT_1_C](javascript:makeHelpWindow(%22CRYSC_01.html%22)) No recognised colour has been given for crystal colour.

[PLAT245_ALERT_2_C](javascript:makeHelpWindow(%22PLAT245.html%22)) U(iso) H1 Smaller than U(eq) C1 by 0.015 Ang**2

[PLAT245_ALERT_2_C](javascript:makeHelpWindow(%22PLAT245.html%22)) U(iso) H3 Smaller than U(eq) C3 by 0.012 Ang**2

[PLAT342_ALERT_3_C](javascript:makeHelpWindow(%22PLAT342.html%22)) Low Bond Precision on C-C Bonds ............... 0.00831 Ang.

[PLAT350_ALERT_3_C](javascript:makeHelpWindow(%22PLAT350.html%22)) Short C-H (X0.96,N1.08A) C4 - H4 . 0.79 Ang.

[PLAT906_ALERT_3_C](javascript:makeHelpWindow(%22PLAT906.html%22)) Large K Value in the Analysis of Variance ...... 3.098 Check

[PLAT971_ALERT_2_C](javascript:makeHelpWindow(%22PLAT971.html%22)) Check Calcd Resid. Dens. 0.83Ang From Cs2 1.89 eA-3

[PLAT971_ALERT_2_C](javascript:makeHelpWindow(%22PLAT971.html%22)) Check Calcd Resid. Dens. 0.91Ang From Cs1 1.51 eA-3


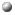
**Alert level G**

[PLAT003_ALERT_2_G](javascript:makeHelpWindow(%22PLAT003.html%22)) Number of Uiso or Uij Restrained non-H Atoms ... 17 Report

[PLAT004_ALERT_5_G](javascript:makeHelpWindow(%22PLAT004.html%22)) Polymeric Structure Found with Maximum Dimension 2 Info

[PLAT045_ALERT_1_G](javascript:makeHelpWindow(%22PLAT045.html%22)) Calculated and Reported Z Differ by a Factor ... 0.250 Check

[PLAT083_ALERT_2_G](javascript:makeHelpWindow(%22PLAT083.html%22)) SHELXL Second Parameter in WGHT Unusually Large 6.72 Why ?

[PLAT142_ALERT_4_G](javascript:makeHelpWindow(%22PLAT142.html%22)) s.u. on b - Axis Small or Missing .............. 0.00010 Ang.

[PLAT143_ALERT_4_G](javascript:makeHelpWindow(%22PLAT143.html%22)) s.u. on c - Axis Small or Missing .............. 0.00020 Ang.

[PLAT164_ALERT_4_G](javascript:makeHelpWindow(%22PLAT164.html%22)) Nr. of Refined C-H H-Atoms in Heavy-Atom Struct. 10 Note

[PLAT171_ALERT_4_G](javascript:makeHelpWindow(%22PLAT171.html%22)) The CIF-Embedded .res File Contains EADP Records 6 Report

[PLAT177_ALERT_4_G](javascript:makeHelpWindow(%22PLAT177.html%22)) The CIF-Embedded .res File Contains DELU Records 3 Report

[PLAT178_ALERT_4_G](javascript:makeHelpWindow(%22PLAT178.html%22)) The CIF-Embedded .res File Contains SIMU Records 3 Report

[PLAT186_ALERT_4_G](javascript:makeHelpWindow(%22PLAT186.html%22)) The CIF-Embedded .res File Contains ISOR Records 1 Report

[PLAT300_ALERT_4_G](javascript:makeHelpWindow(%22PLAT300.html%22)) Atom Site Occupancy of N3 Constrained at 0.5 Check

**And 32 other PLAT300 Alerts**

More ...

[PLAT301_ALERT_3_G](javascript:makeHelpWindow(%22PLAT301.html%22)) Main Residue Disorder ..............(Resd 1 ) 21% Note

[PLAT302_ALERT_4_G](javascript:makeHelpWindow(%22PLAT302.html%22)) Anion/Solvent/Minor-Residue Disorder (Resd 2 ) 100% Note

[PLAT343_ALERT_2_G](javascript:makeHelpWindow(%22PLAT343.html%22)) Unusual Angle Range in Main Residue for C5 Check

**And 3 other PLAT343 Alerts**

More ...

[PLAT410_ALERT_2_G](javascript:makeHelpWindow(%22PLAT410.html%22)) Short Intra H...H Contact H13 ..H24 . 2.14 Ang.

x,y,z = 1_555 Check

[PLAT764_ALERT_4_G](javascript:makeHelpWindow(%22PLAT764.html%22)) Overcomplete CIF Bond List Detected (Rep/Expd) . 1.15 Ratio

[PLAT774_ALERT_1_G](javascript:makeHelpWindow(%22PLAT774.html%22)) Check X-Y Bond in CIF: Cs1 --Cs2 .. 4.54 Ang.

**And 2 other PLAT774 Alerts**

More ...

[PLAT789_ALERT_4_G](javascript:makeHelpWindow(%22PLAT789.html%22)) Atoms with Negative _atom_site_disorder_group # 11 Check

[PLAT811_ALERT_5_G](javascript:makeHelpWindow(%22PLAT811.html%22)) No ADDSYM Analysis: Too Many Excluded Atoms .... ! Info

[PLAT860_ALERT_3_G](javascript:makeHelpWindow(%22PLAT860.html%22)) Number of Least-Squares Restraints ............. 175 Note

[PLAT883_ALERT_1_G](javascript:makeHelpWindow(%22PLAT883.html%22)) No Info/Value for _atom_sites_solution_primary . Please Do !

[PLAT912_ALERT_4_G](javascript:makeHelpWindow(%22PLAT912.html%22)) Missing # of FCF Reflections Above STh/L= 0.600 11 Note

[PLAT933_ALERT_2_G](javascript:makeHelpWindow(%22PLAT933.html%22)) Number of HKL-OMIT Records in Embedded .res File 1 Note

[PLAT978_ALERT_2_G](javascript:makeHelpWindow(%22PLAT978.html%22)) Number C-C Bonds with Positive Residual Density. 2 Info

0 **ALERT level A** = Most likely a serious problem - resolve or explain

0 **ALERT level B** = A potentially serious problem, consider carefully

9 **ALERT level C** = Check. Ensure it is not caused by an omission or oversight

62 **ALERT level G** = General information/check it is not something unexpected

7 ALERT type 1 CIF construction/syntax error, inconsistent or missing data

13 ALERT type 2 Indicator that the structure model may be wrong or deficient

5 ALERT type 3 Indicator that the structure quality may be low

44 ALERT type 4 Improvement, methodology, query or suggestion

2 ALERT type 5 Informative message, check

| It is advisable to attempt to resolve as many as possible of the alerts in all categories. Often the minor alerts point to easily fixed oversights, errors and omissions in your CIF or refinement strategy, so attention to these fine details can be worthwhile. In order to resolve some of the more serious problems it may be necessary to carry out additional measurements or structure refinements. However, the purpose of your study may justify the reported deviations and the more serious of these should normally be commented upon in the discussion or experimental section of a paper or in the "special_details" fields of the CIF. checkCIF was carefully designed to identify outliers and unusual parameters, but every test has its limitations and alerts that are not important in a particular case may appear. Conversely, the absence of alerts does not guarantee there are no aspects of the results needing attention. It is up to the individual to critically assess their own results and, if necessary, seek expert advice.  **Publication of your CIF in IUCr journals**  A basic structural check has been run on your CIF. These basic checks will be run on all CIFs submitted for publication in IUCr journals (*Acta Crystallographica*, *Journal of Applied Crystallography*, *Journal of Synchrotron Radiation*); however, if you intend to submit to *Acta Crystallographica Section C* or *E* or *IUCrData*, you should make sure that [full publication checks](http://journals.iucr.org/services/cif/checking/checkform.html) are run on the final version of your CIF prior to submission.  **Publication of your CIF in other journals**  Please refer to the *Notes for Authors* of the relevant journal for any special instructions relating to CIF submission. |
| --- |
