# Supplementary material for: Alkali Metal Dihydropyridines in Transfer Hydrogenation Catalysis of Imines: Amide Basicity versus Hydride Surrogacy
Source: Angew Chem Int Ed Engl. 2023 May 22;62(27):e202304966. doi: 10.1002/anie.202304966 (PMC10952797; doi:10.1002/anie.202304966)
Supplement: Supplementary file 3 — Supporting Information [file ANIE-62-0-s003.pdf]

Angewandte Chemie
Eine Zeitschrift der Gesellschaft Deutscher Chemiker

Supporting Information

Alkali Metal Dihydropyridines in Transfer Hydrogenation Catalysis of Imines: Amide Basicity versus Hydride Surrogacy

P. A. Macdonald, S. Banerjee, A. R. Kennedy, A. van Teijlingen, S. D. Robertson, T. Tuttle*, R. E. Mulvey**

Supporting Information

1. Transfer Hydrogenation Experiments
2. Catalyst Activity
3. Stoichiometric Reactions
4. Crystal Structures of $[PhCH_2N(Li)Ph]_\infty$ (**1**) and $[PhCH_2N(Cs)Ph]_\infty$ (**2**)
5. Investigation of Mechanism
6. $\{[Cs(tBuDHP)]_2\cdot py\}_\infty$ (**3**): Solid-state structure
7. $[Li(tBuDHP)\cdot(py)_3]$ (**4**): Solid-state structure
8. Experiments supporting proposed mechanistic cycle
9. Base mediated initiation pathway (BMIP) & surrogate hydride initiation pathway (SHIP) using potassium and lithium
10. Parallel H_2 pathway
11. Deep neural network optimiser
12. General experimental procedures
13. Synthetic procedures
14. References

1. Transfer hydrogenation experiments

1.1 *N*-Benzylideneaniline Catalysis

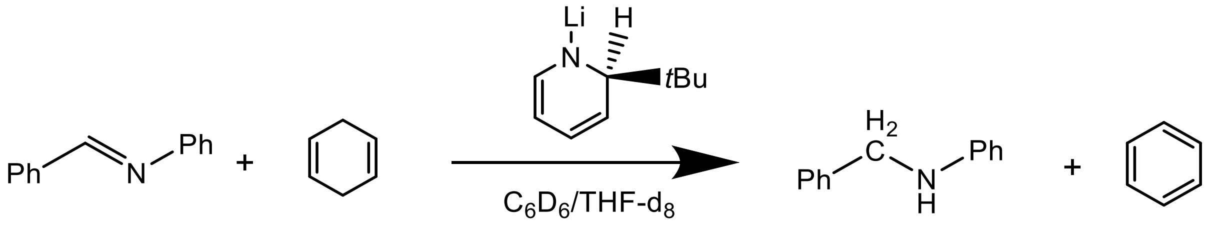

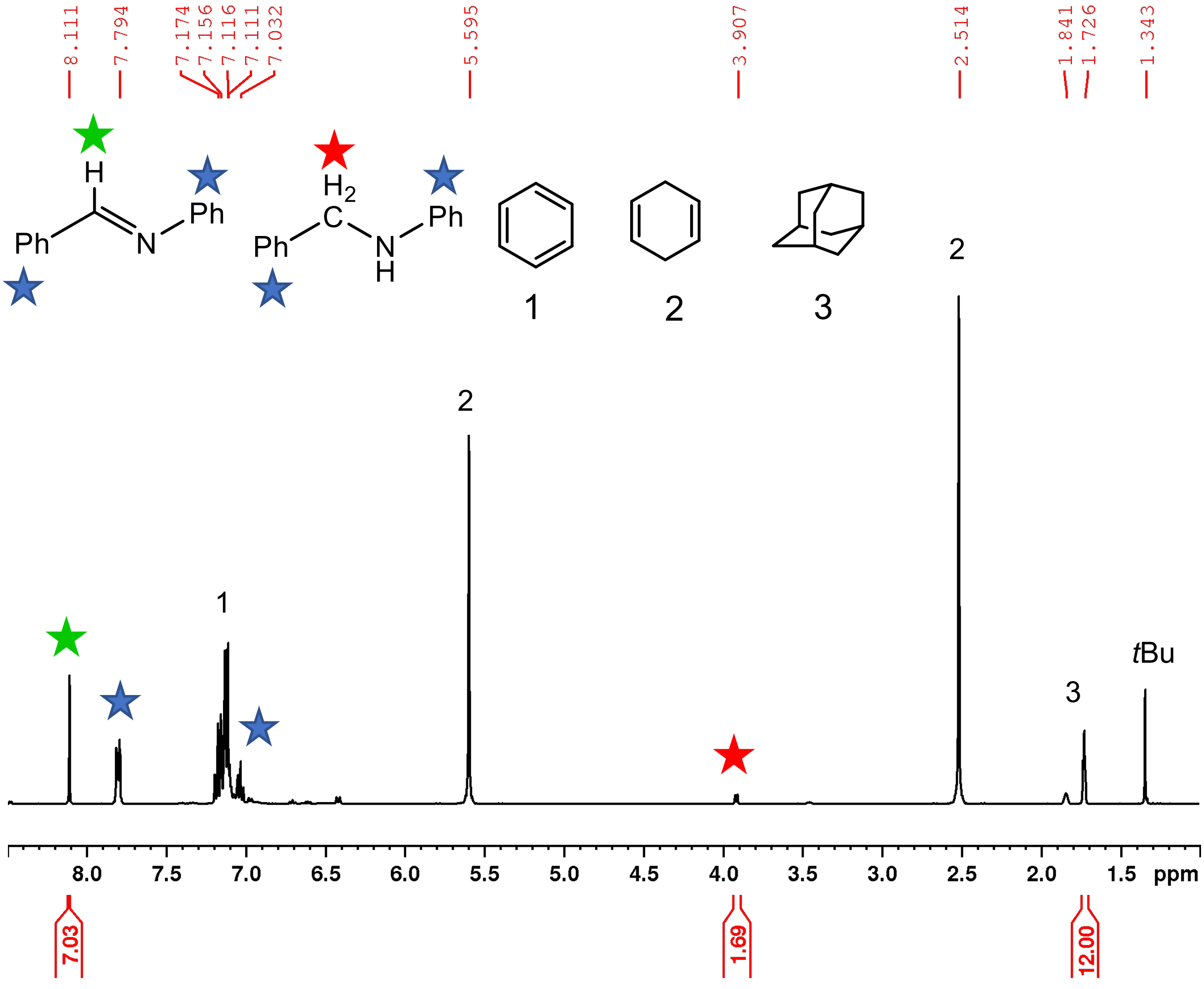

Figure S1 ^{1}H NMR spectrum of the completed transfer hydrogenation reaction between *N*-benzylideneaniline (0.3 mmol) and Li(*t*BuDHP) (10 mol%) as catalyst using 1.5 equiv. of 1,4-cyclohexadiene in C_6D_6 showing the partial formation of the amine product after heating for 24 hours at 70 °C. Adamantane standard was used (0.0375 mmol) to calculate the percentage yield. (Entry 1, Yield 21%)

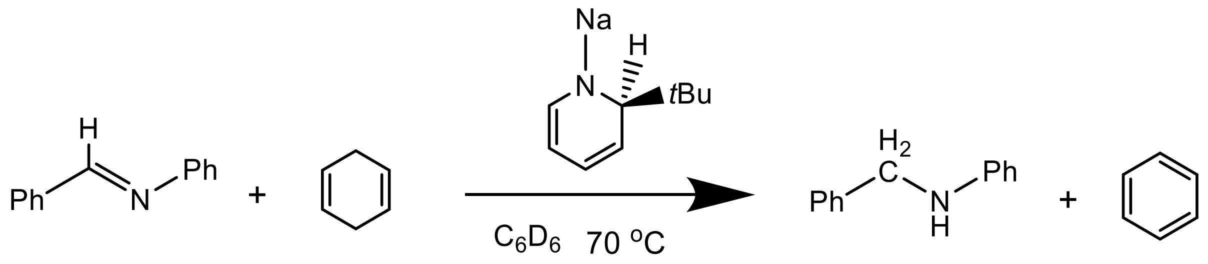

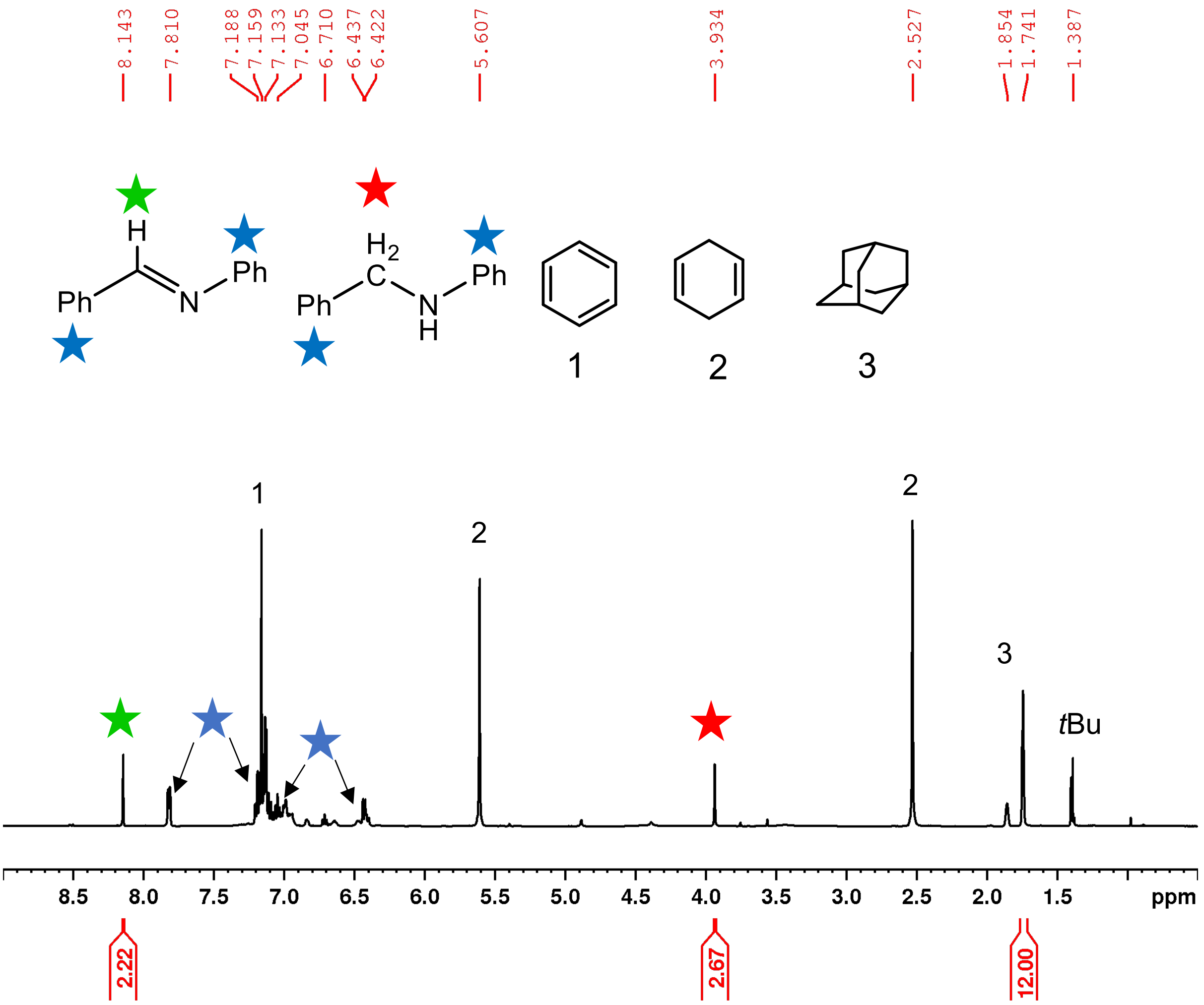

Figure S2 ^{1}H NMR spectrum of the completed transfer hydrogenation reaction between *N*-benzylideneaniline (0.3 mmol) and 1.5 equiv. of 1,4-cyclohexadiene using Na(*t*BuDHP) (10 mol%) as catalyst in C_6D_6 showing the partial formation of the amine product after heating for 24 hours at 70 °C. Adamantane standard was used (0.065 mmol) to calculate the percentage yield. (Entry 2, Yield 57%)

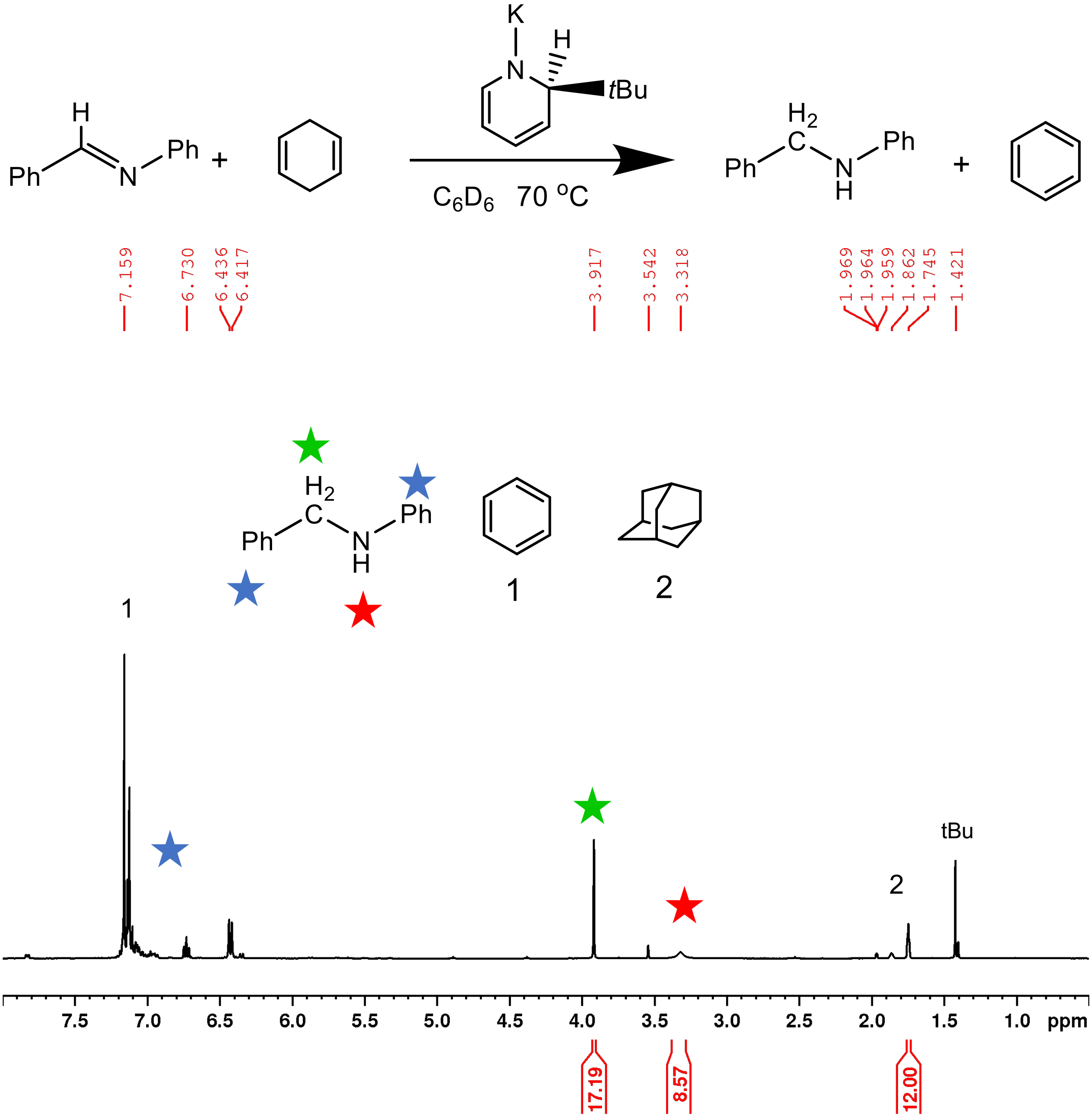

Figure S3 ^{1}H NMR spectrum of the completed transfer hydrogenation reaction between *N*-benzylideneaniline (0.3 mmol) and 1.5 equiv. of 1,4-cyclohexadiene using K(*t*BuDHP) (10 mol%) as catalyst in C_6D_6 showing the formation of the amine product after heating for 16 hours at 70 °C. Adamantane standard was used (0.03 mmol) to calculate the percentage yield. (Entry 3, Yield 85%)

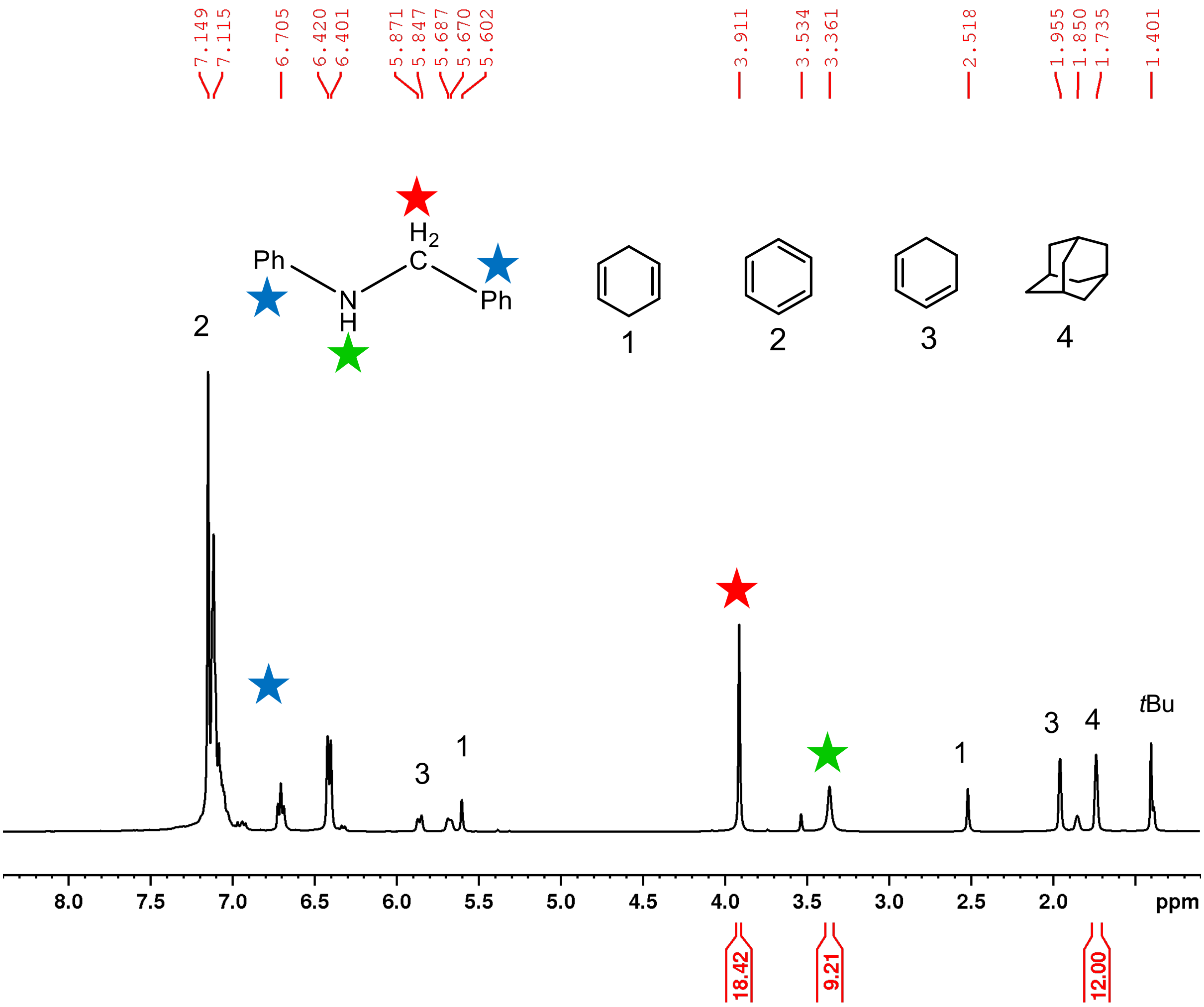

Figure S4 ^{1}H NMR spectrum of the completed transfer hydrogenation reaction between *N*-benzylideneaniline (0.3 mmol) and 1.5 equiv. of 1,4-cyclohexadiene using Rb(*t*BuDHP) (10 mol%) as catalyst in C_6D_6 showing the formation of the amine product after heating for 10 hours at 70 °C. Adamantane standard was used (0.03 mmol) to calculate the percentage yield. (Entry 4, Yield 92%)

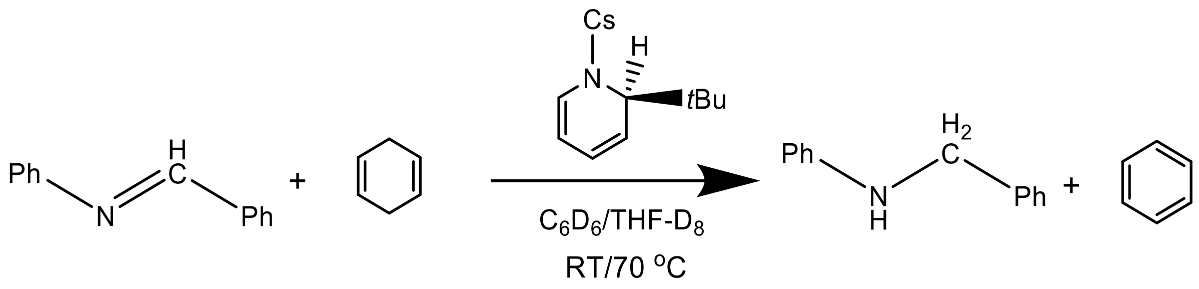

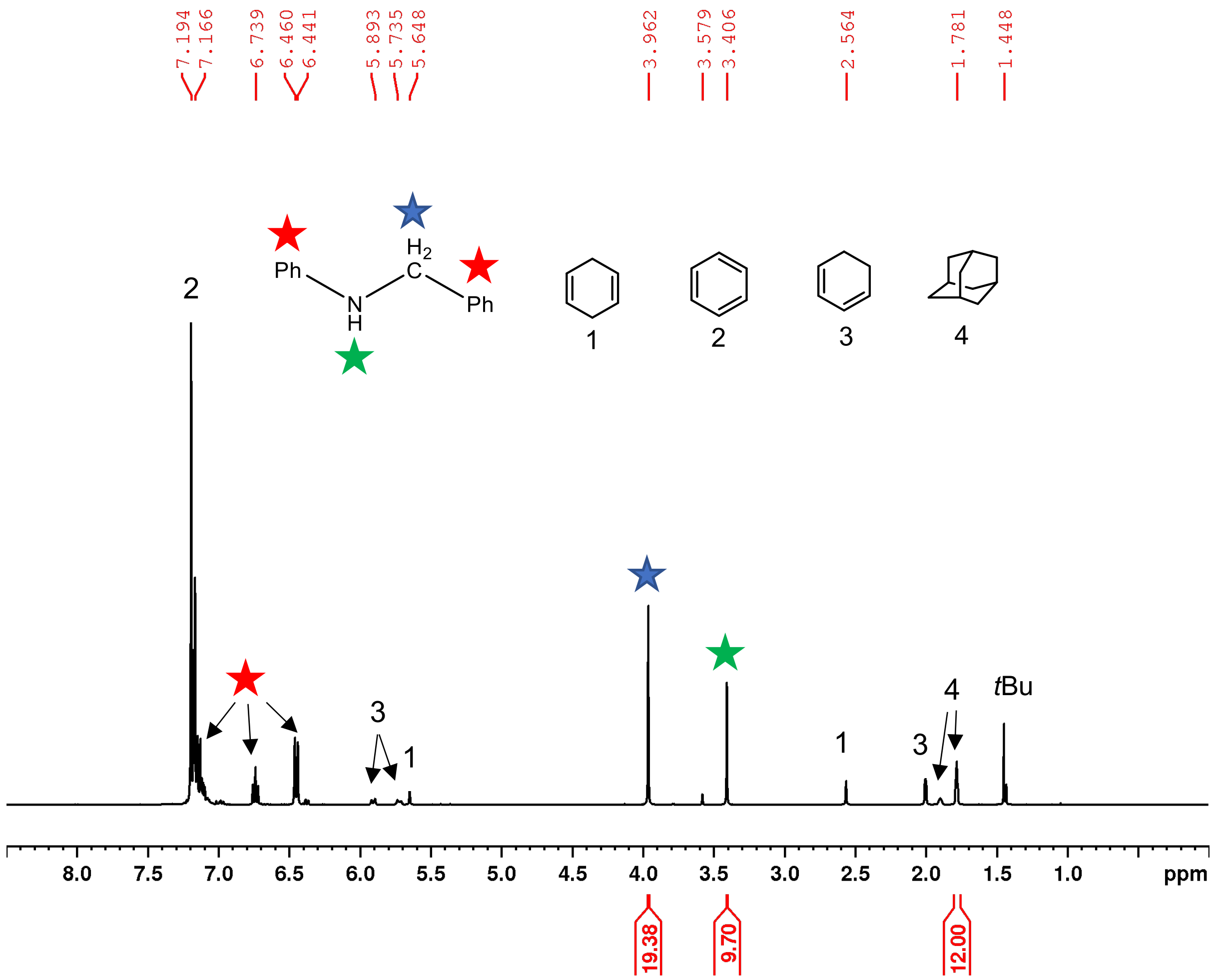

Figure S5 ^{1}H NMR spectrum of the completed transfer hydrogenation reaction between *N*-benzylideneaniline (0.3 mmol) and 1.5 equiv. of 1,4-cyclohexadiene using Cs(*t*BuDHP) (10 mol%) as catalyst in C_6D_6 showing the formation of the amine product after heating for 1 hour at 70 °C. Isomer products for the formation of 1,3-cyclohexadiene have also been shown. Adamantane (0.03 mmol) standard has been used to calculate the percentage yield. (Entry 5, Yield 97%)

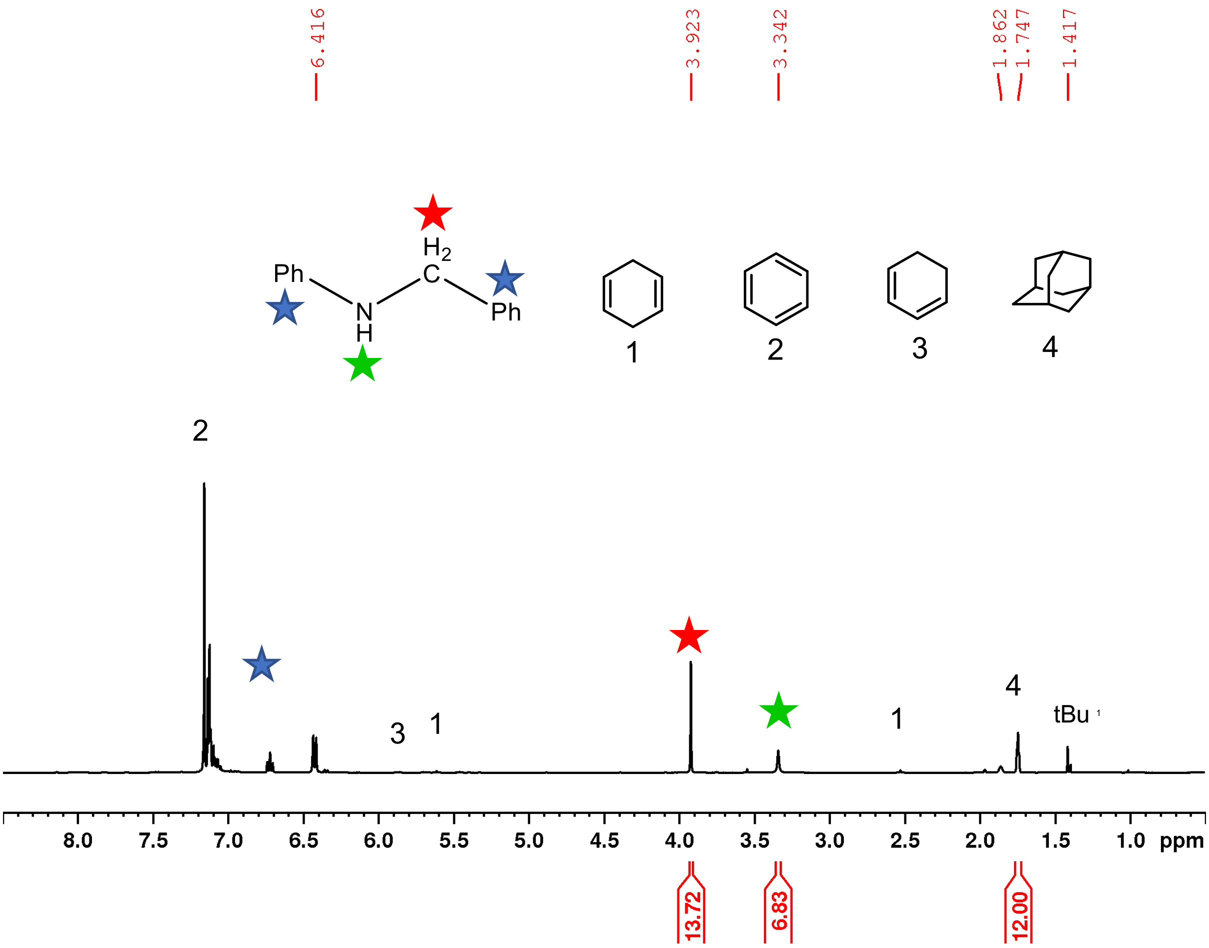

Figure S6 ^{1}H NMR spectrum of the completed transfer hydrogenation reaction between *N*-benzylideneaniline (0.3 mmol) and 1.5 equiv. of 1,4-cyclohexadiene using Cs(*t*BuDHP) (5 mol%) as catalyst in C_6D_6 showing the formation of the amine product after heating for 1 hour at 70 °C. Adamantane (0.042 mmol) standard has been used to calculate the percentage yield (Entry 6, Yield 96%).

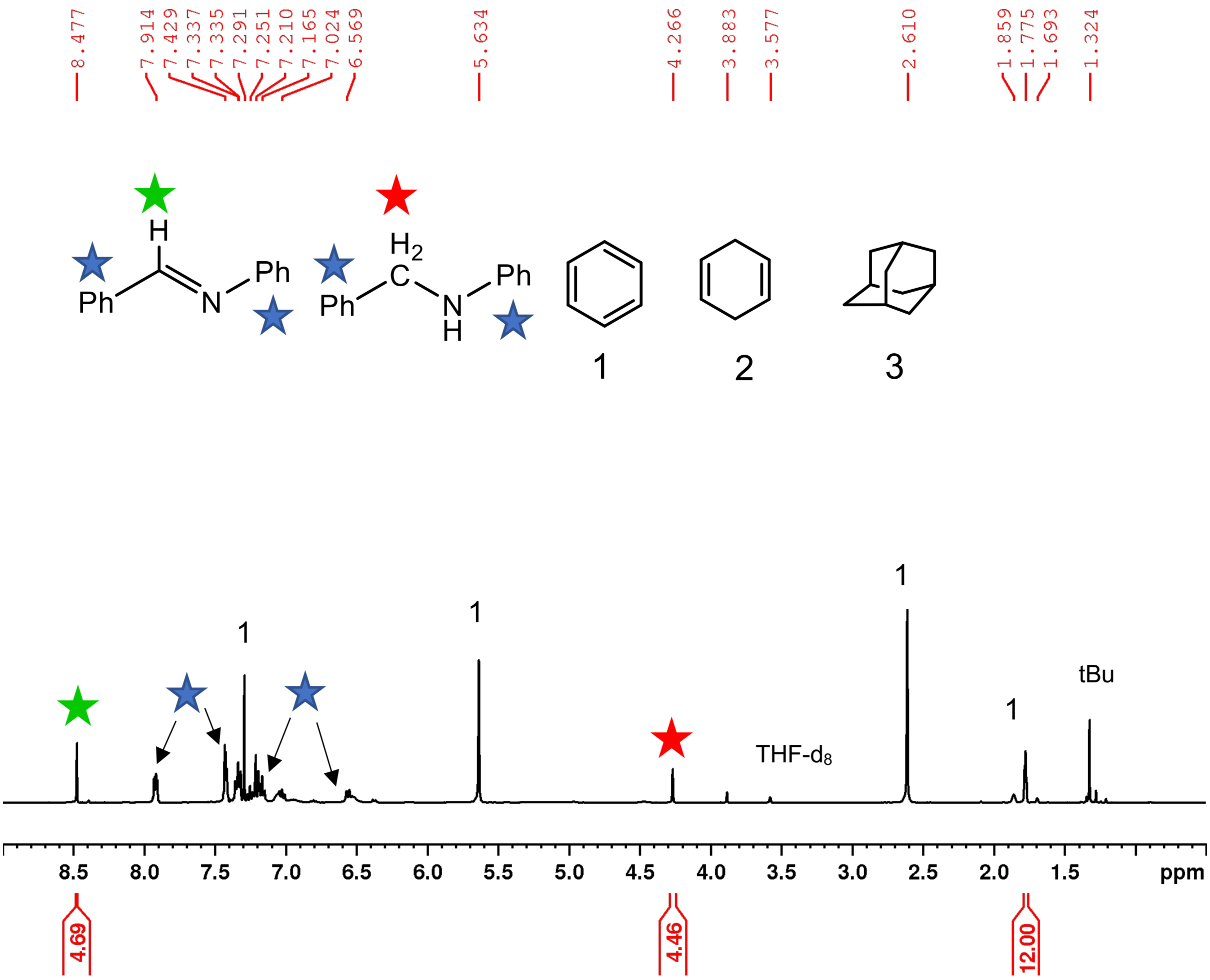

Figure S7 ^{1}H NMR spectrum of the completed transfer hydrogenation reaction between *N*-benzylideneaniline (0.3 mmol) and Li(*t*BuDHP) (10 mol%) as catalyst using 1.5 equiv. of 1,4-cyclohexadiene in THF-d_8 showing the partial formation of the amine product after heating for 24 hours at 70 °C. Adamantane standard was used (0.0375 mmol) to calculate the percentage yield. (Entry 7, Yield 56%)

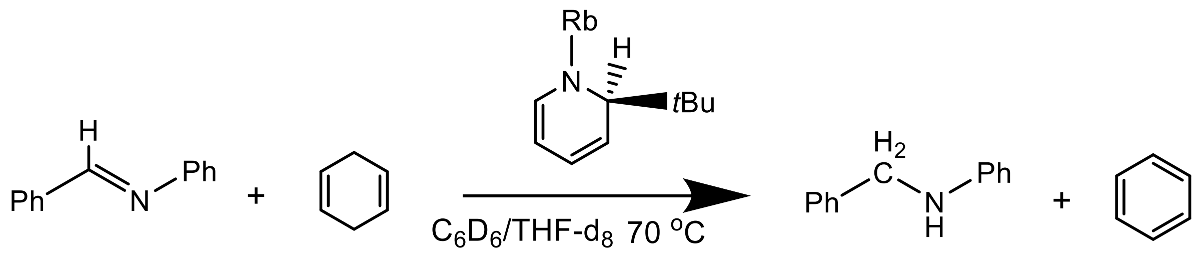

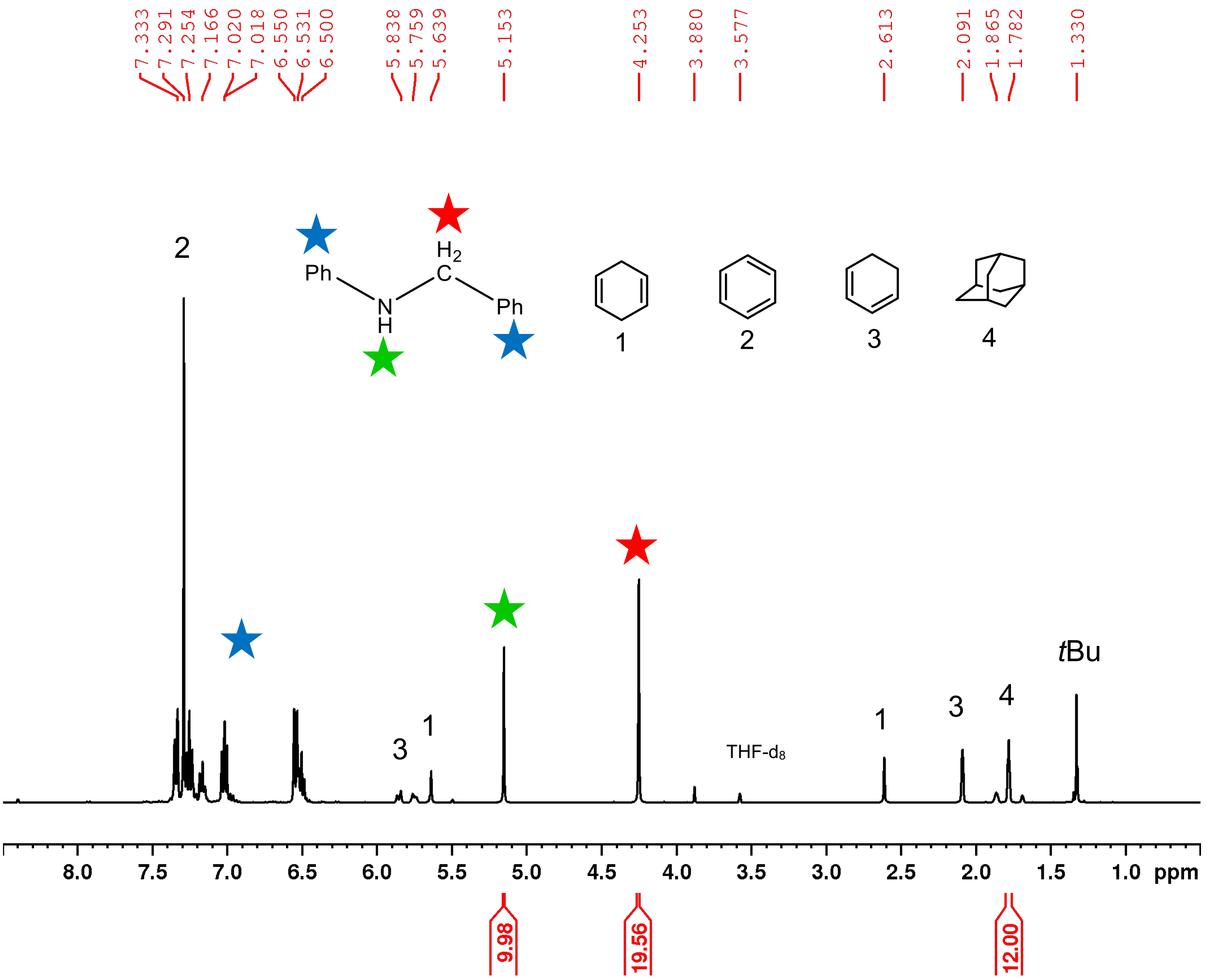

Figure S8 ^{1}H NMR spectrum of the completed transfer hydrogenation reaction between *N*-benzylideneaniline (0.3 mmol) and 1.5 equiv. of 1,4-cyclohexadiene using Rb(*t*BuDHP) (10 mol%) as catalyst in THF-d_8 showing the formation of the amine product after heating for 2.5 hours at 70 °C. Adamantane standard was used (0.03 mmol) to calculate the percentage yield. (Entry 8, Yield 98%)

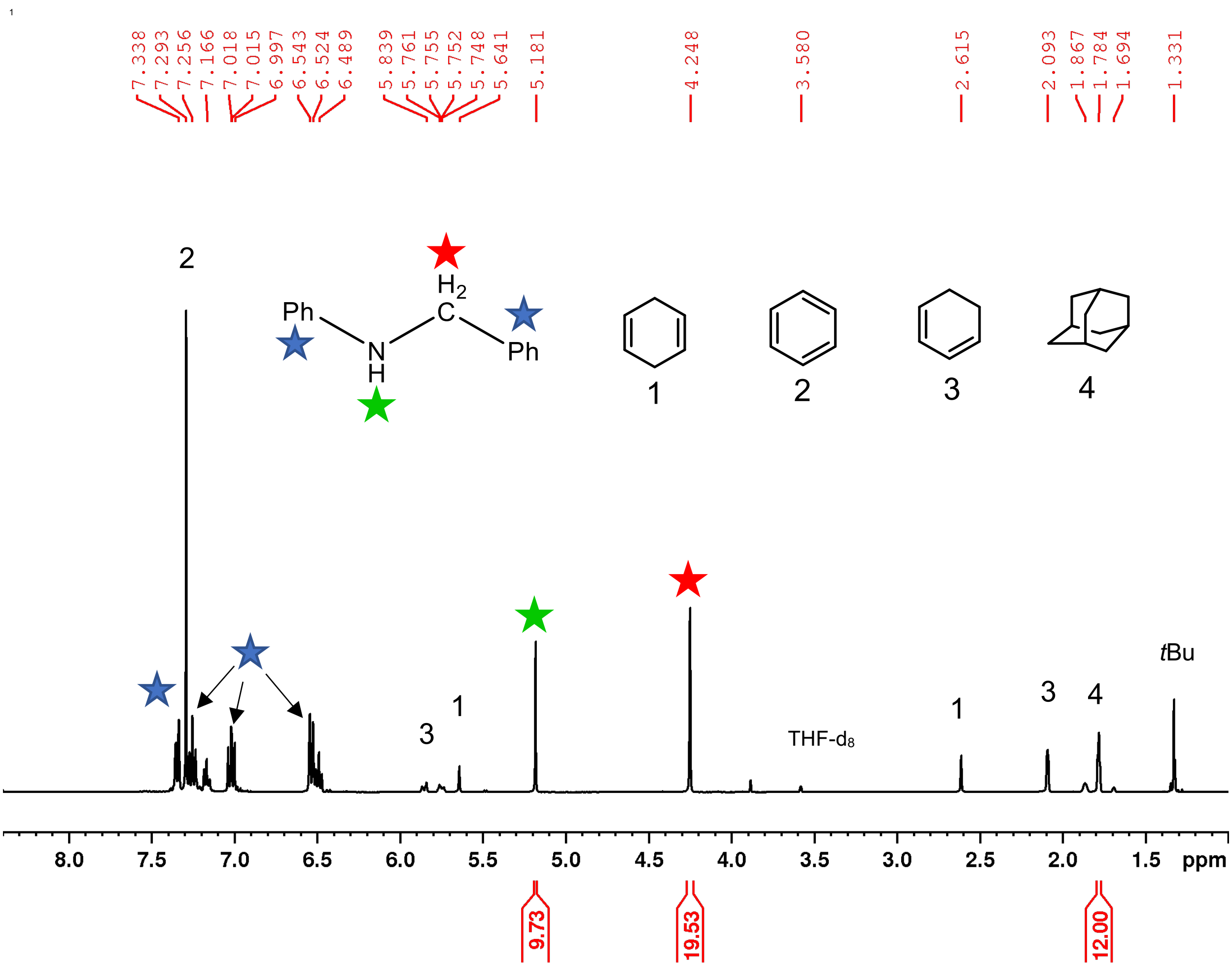

Figure S9 ^{1}H NMR spectrum of the completed transfer hydrogenation reaction between *N*-benzylideneaniline (0.3 mmol) and 1.5 equiv. of 1,4-cyclohexadiene using Cs(*t*BuDHP) (10 mol%) as catalyst in THF-d_8 showing the formation of the amine product after heating for 0.25 hours at 70 °C. Resonances of isomeric 1,3-cyclohexadiene have also been shown. Adamantane (0.03 mmol) standard has been used to calculate the percentage yield (Entry 9, Yield 97%)

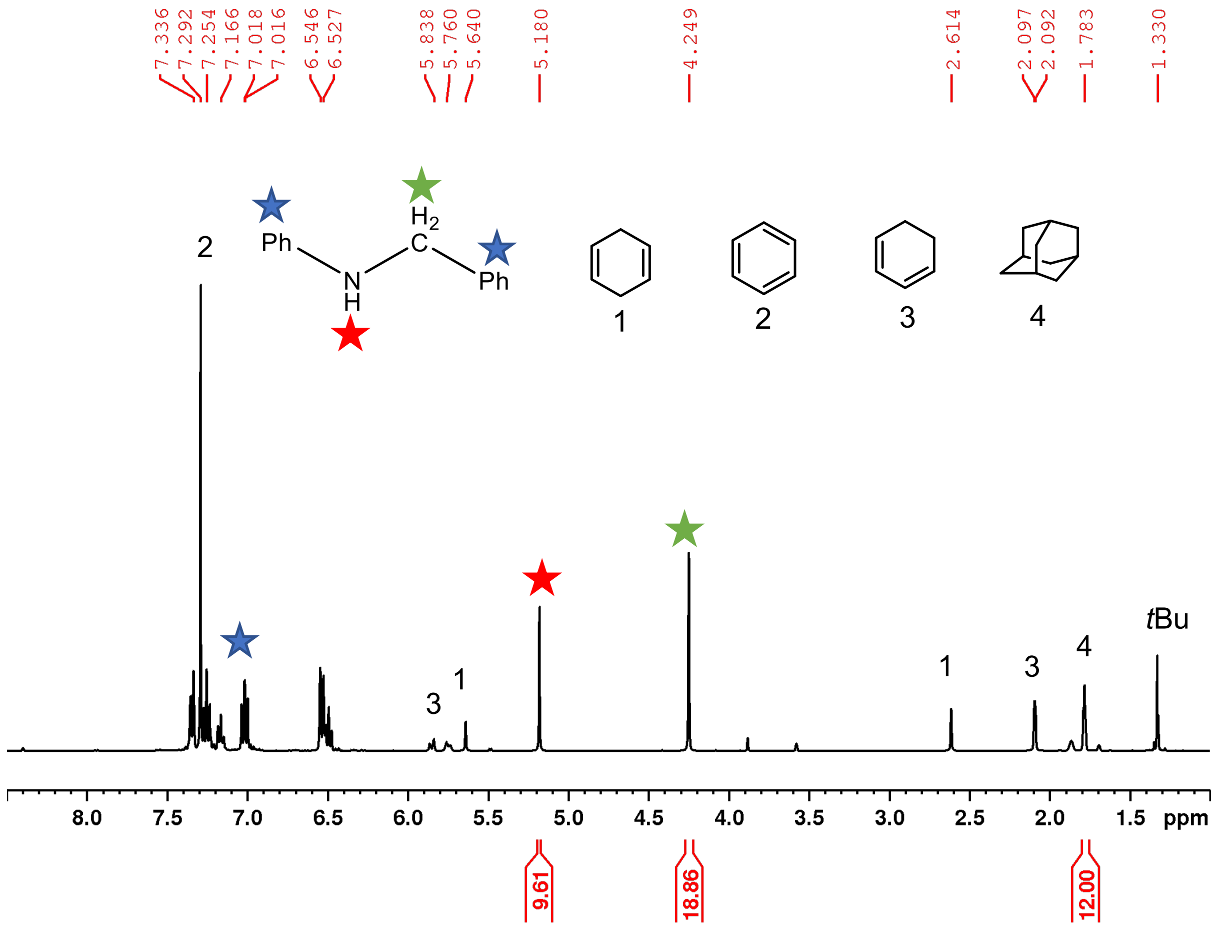

Figure S10 ^{1}H NMR spectrum of the completed transfer hydrogenation reaction between *N*-benzylideneaniline (0.3 mmol) and 1.5 equiv. of 1,4-cyclohexadiene using Cs(*t*BuDHP) (10 mol%) as catalyst in THF-d_8 showing the formation of the amine product after heating for 1 hours at room temperature. Resonances of isomeric 1,3-cyclohexadiene have also been shown. Adamantane (0.03 mmol) standard has been used to calculate the percentage yield (Entry 10, Yield 96%)

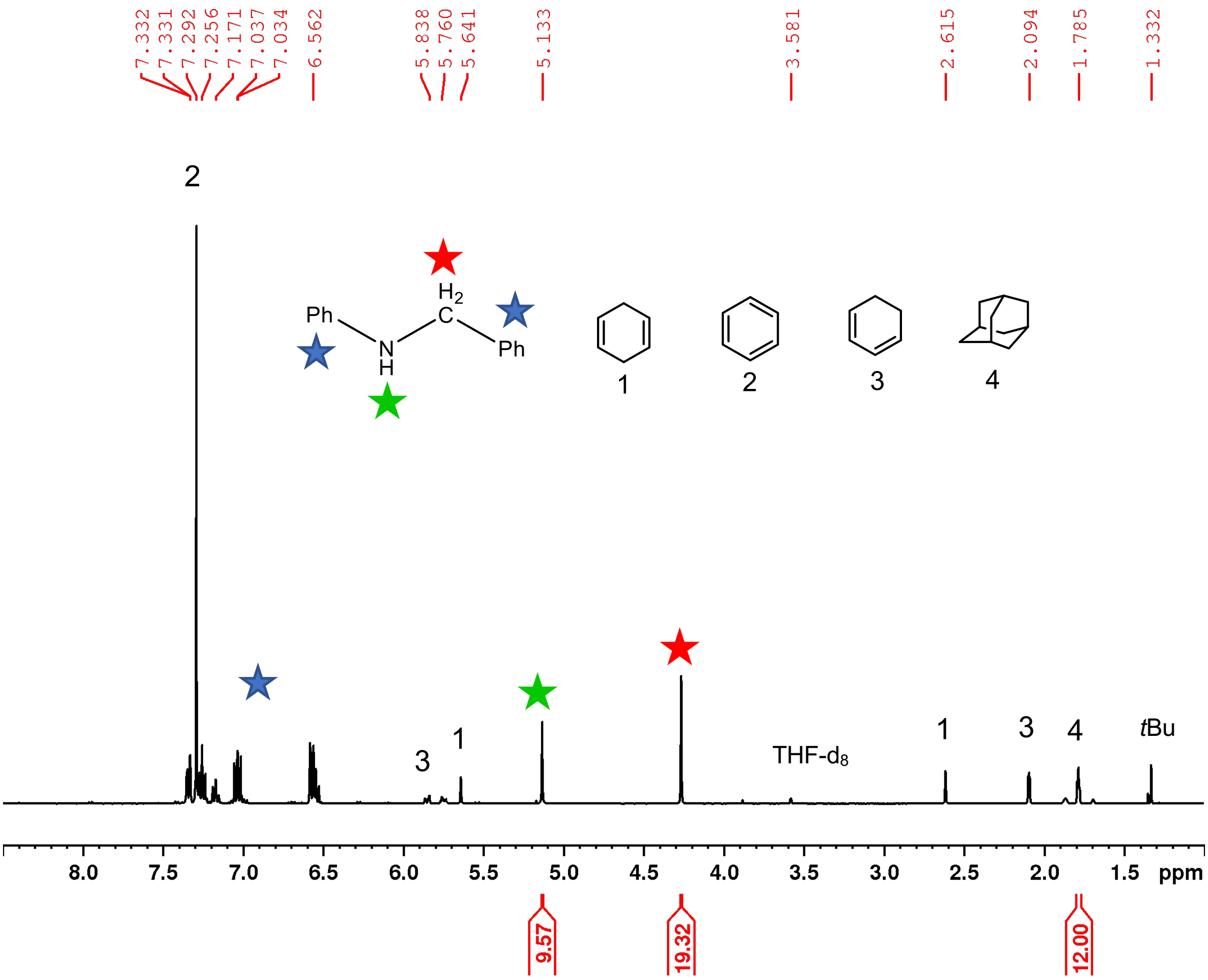

Figure S11 ^{1}H NMR spectrum of the completed transfer hydrogenation reaction between *N*-benzylideneaniline (0.3 mmol) and 1.5 equiv. of 1,4-cyclohexadiene using Cs(*t*BuDHP) (5 mol%) as catalyst in THF-d_8 showing the formation of the amine product after heating for 0.25 hours at 70 °C. Resonances of isomeric 1,3-cyclohexadiene have also been shown. Adamantane (0.03 mmol) standard has been used to calculate the percentage yield (Entry 11, Yield 96%)

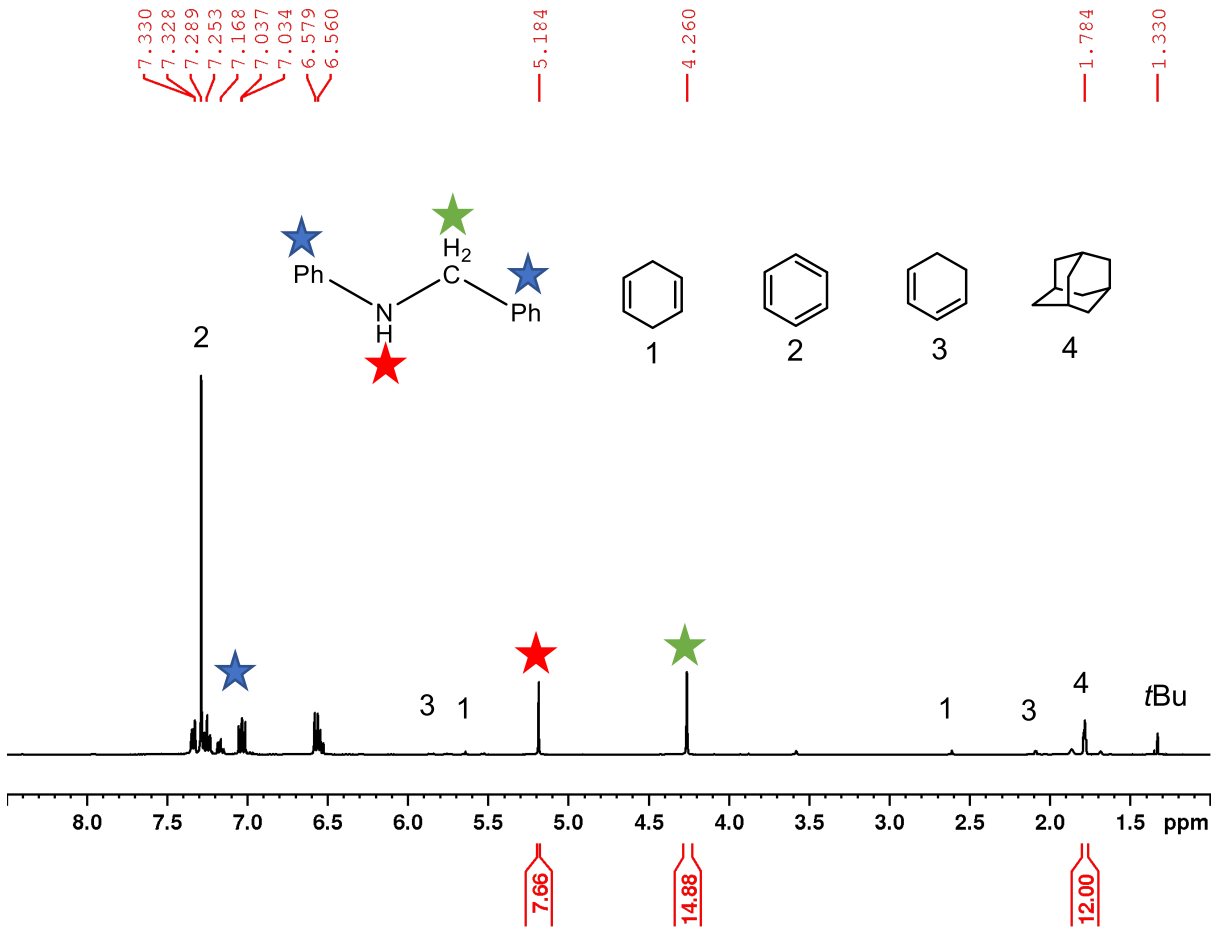

Figure S12 ^{1}H NMR spectrum of the completed transfer hydrogenation reaction between *N*-benzylideneaniline (0.3 mmol) and 1.5 equiv. of 1,4-cyclohexadiene using Cs(*t*BuDHP) (2.5 mol%) as catalyst in THF-d_8 showing the formation of the amine product after heating for 1.5 hours at 70 ^{o}C. Resonances of isomeric 1,3-cyclohexadiene have also been shown. Adamantane (0.0375 mmol) standard has been used to calculate the percentage yield (Entry 12, Yield 96%)

1.2 *N*-benzylidene-*tert*-Butylamine

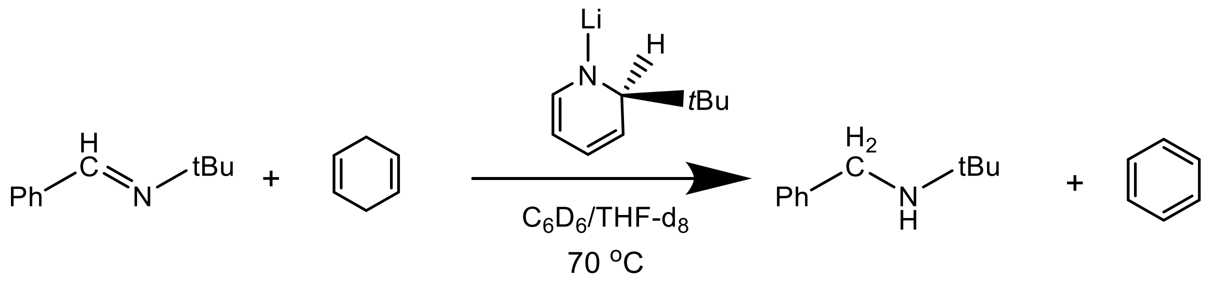

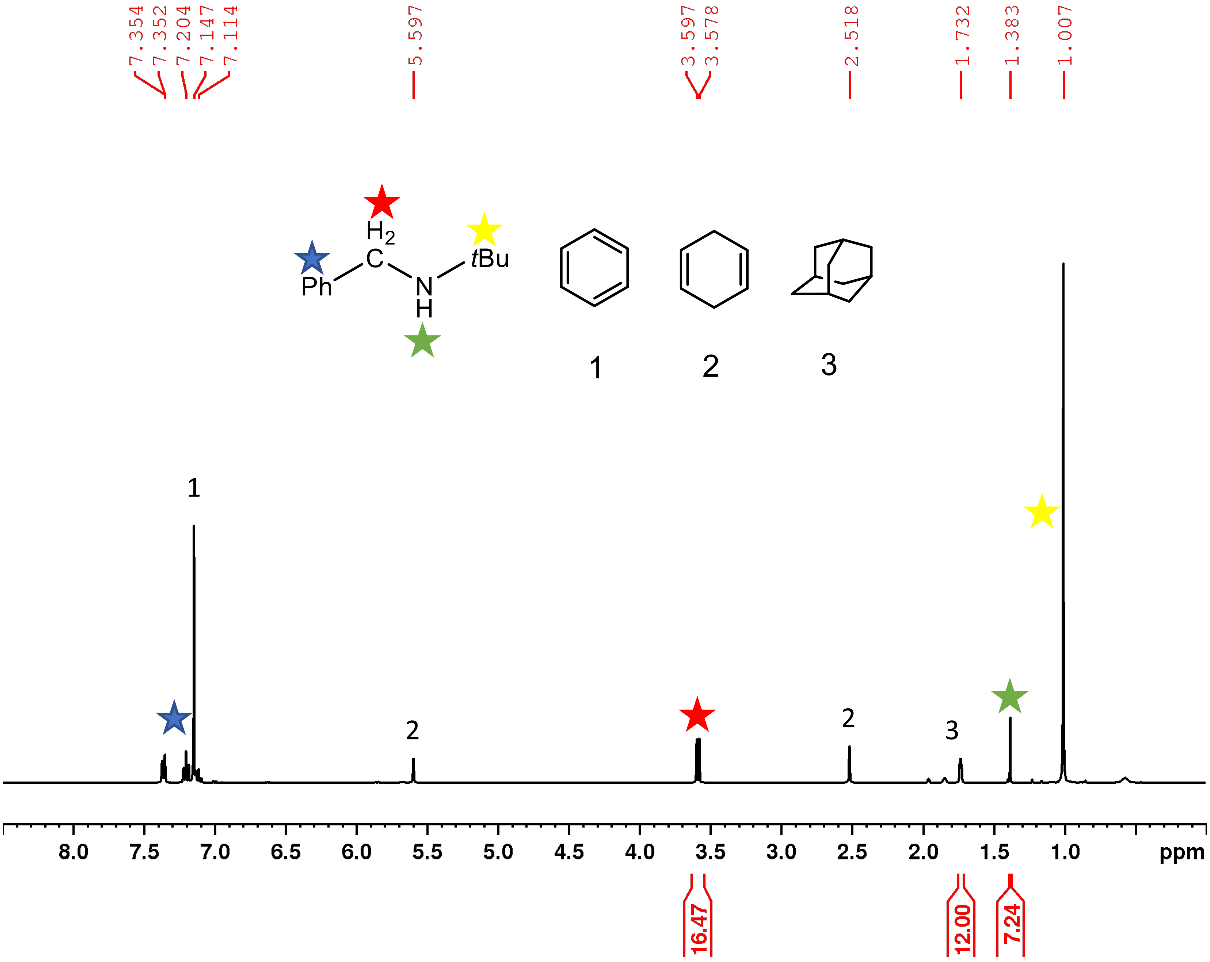

Figure S13 ^{1}H NMR spectrum of the completed transfer hydrogenation reaction between *N*-benzylidene-*tert*-butylamine (0.3 mmol) and Li(*t*BuDHP) (5 mol%) as catalyst using 1.5 equiv. of 1,4-cyclohexadiene in C_6D_6 showing the partial formation of the amine product after heating for 24 hours at 70 °C. Adamantane standard was used (0.033 mmol) to calculate the percentage yield. (Entry 13, Yield 90%)

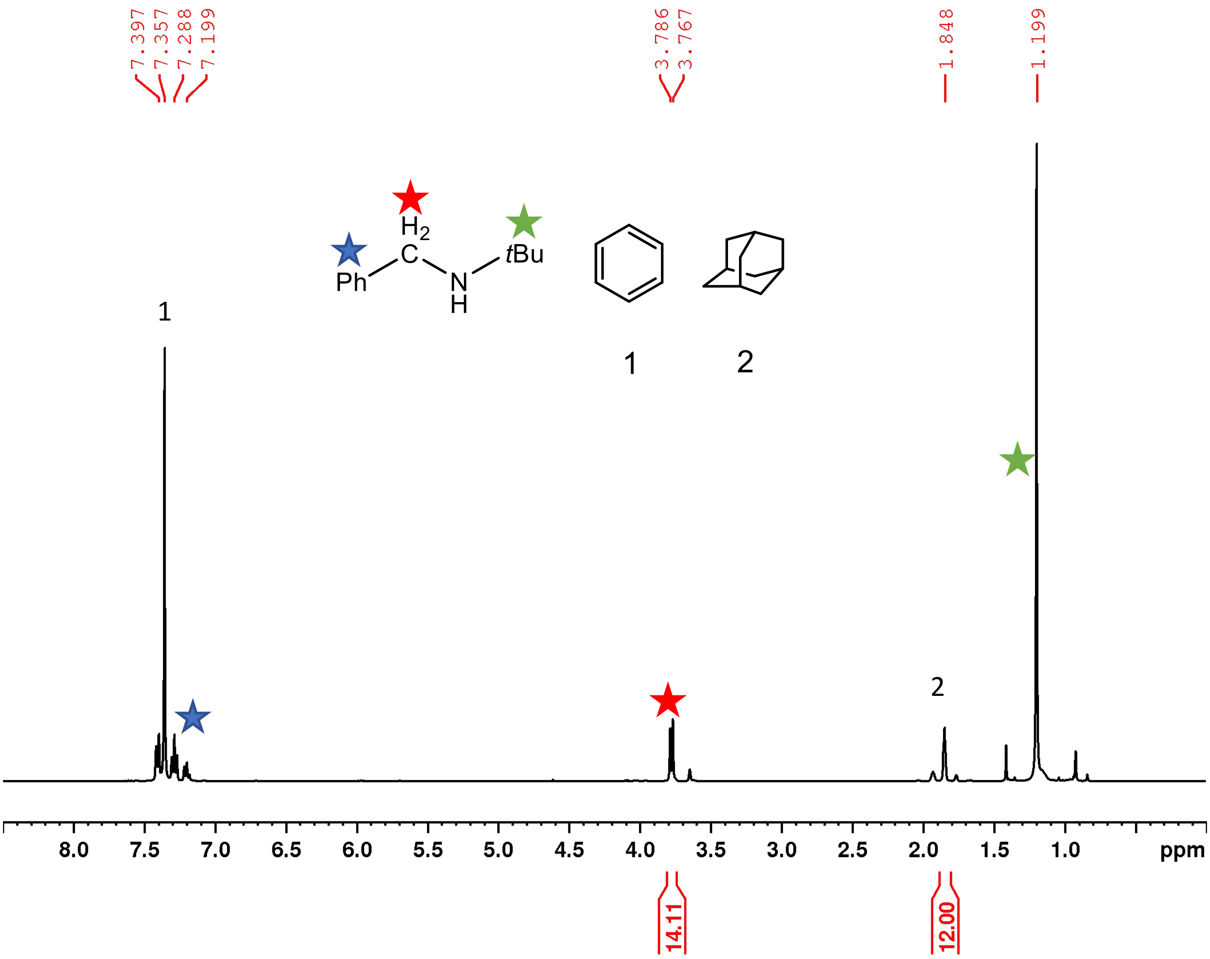

Figure S14 ^{1}H NMR spectrum of the completed transfer hydrogenation reaction between *N*-benzylidene-*tert*-butylamine (0.3 mmol) and Li(*t*BuDHP) (5 mol%) as catalyst using 1.5 equiv. of 1,4-cyclohexadiene in THF-d_8 showing the partial formation of the amine product after heating for 18 hours at 70 °C. Adamantane standard was used (0.0395 mmol) to calculate the percentage yield. (Entry 14, Yield 93%)

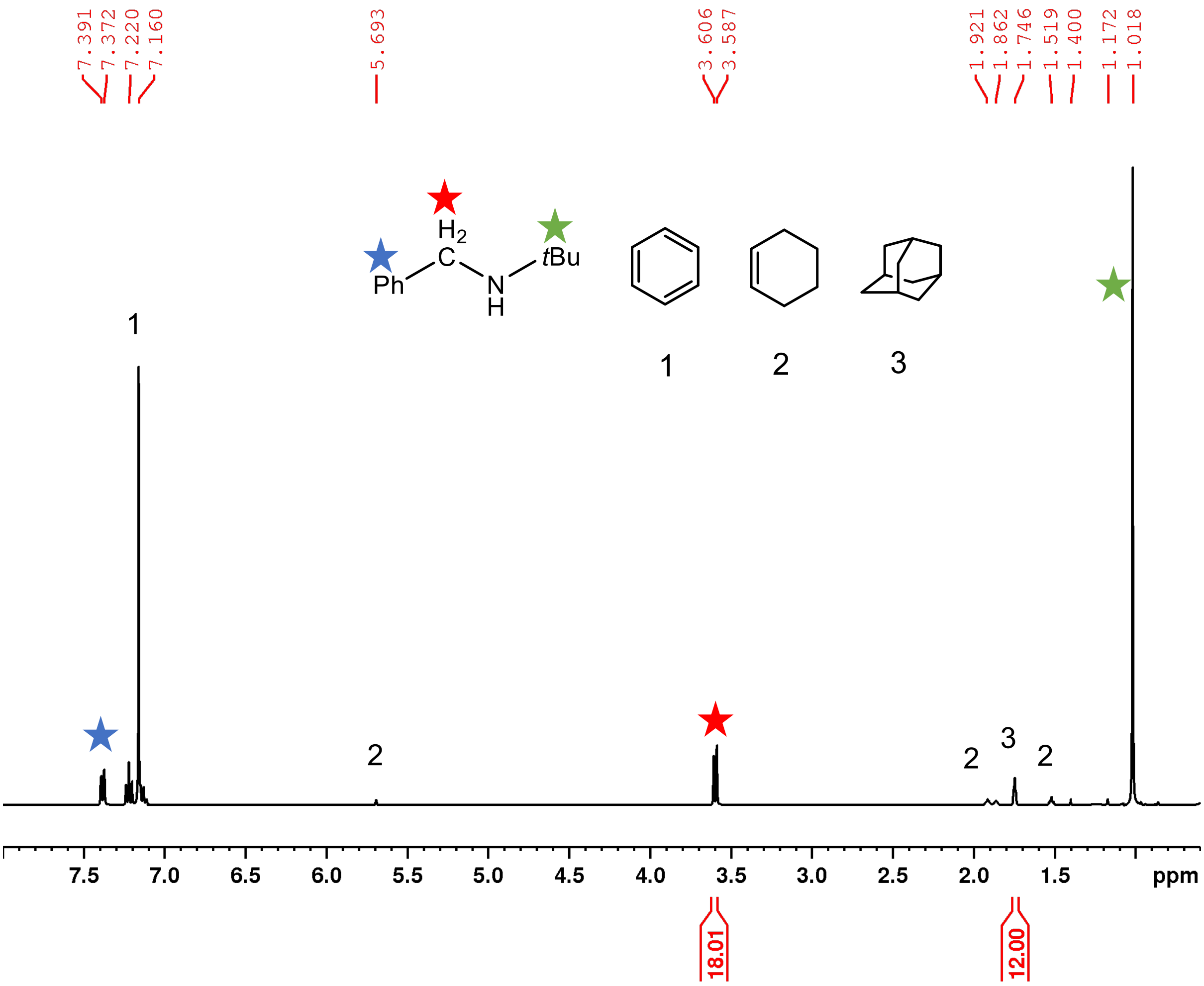

Figure S15 ^{1}H NMR spectrum of the completed transfer hydrogenation reaction between *N*-benzylidene-*tert*-butylamine (0.3 mmol) and 1.5 equiv. of 1,4-cyclohexadiene using Cs(*t*BuDHP) (5 mol%) as catalyst in C_6D_6 showing the formation of the amine product after heating for 3 hours at 70 °C. Adamantane (0.03 mmol) standard has been used to calculate the percentage yield (Entry 15, Yield 91%)

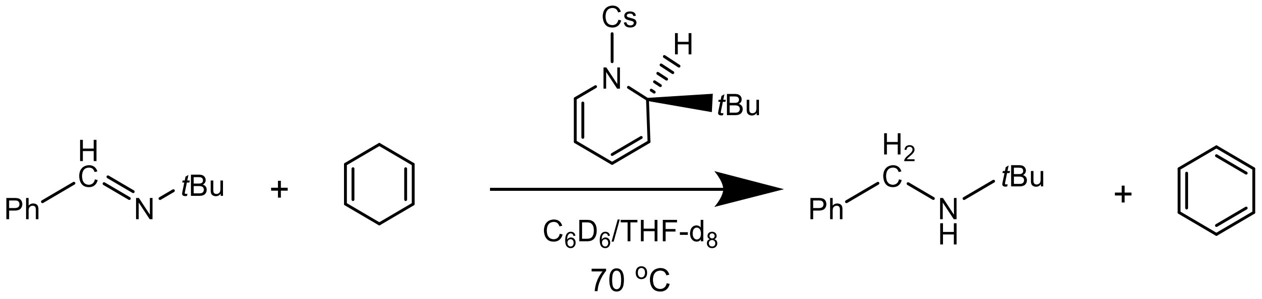

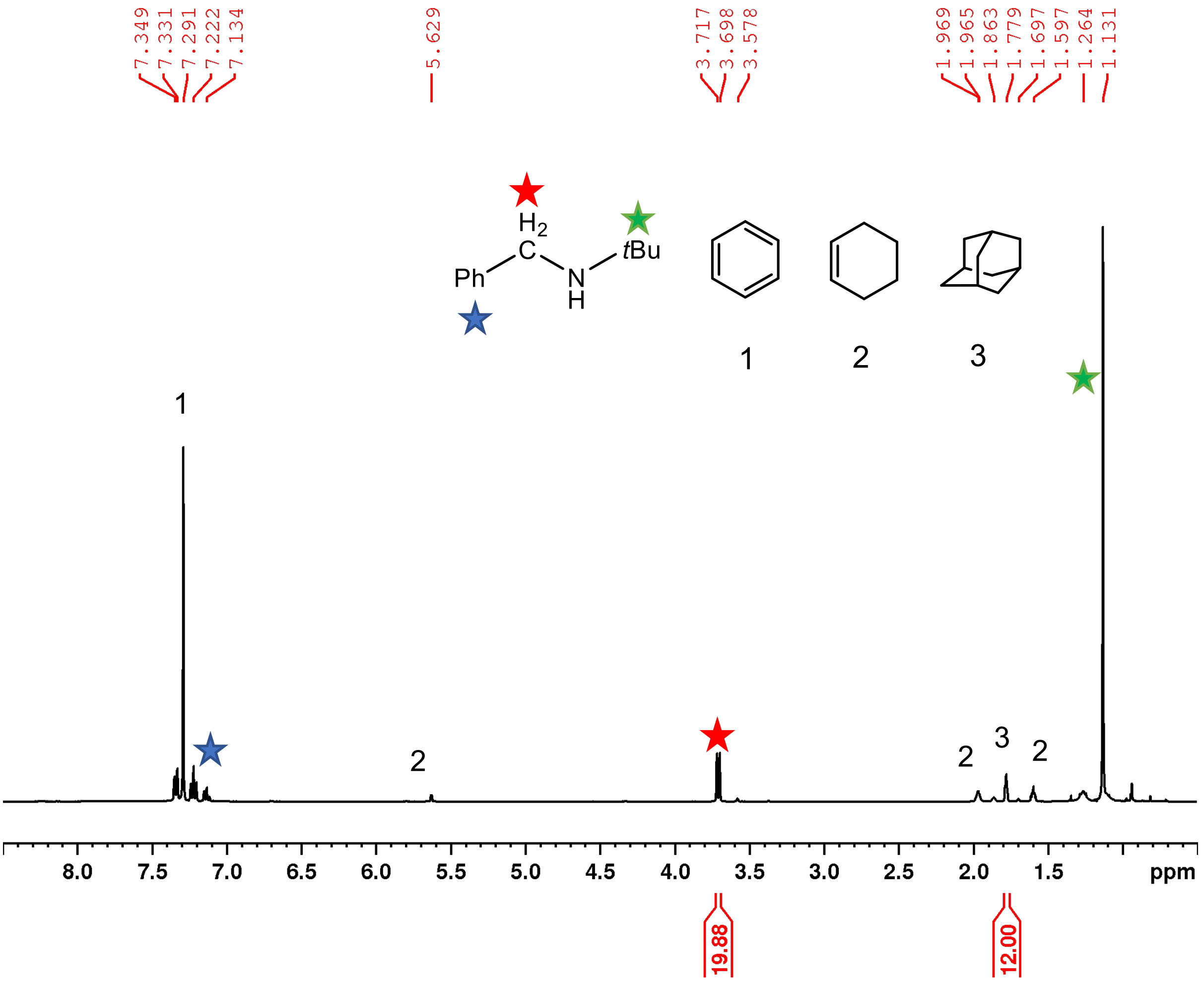

Figure S16 ^{1}H NMR spectrum of the completed transfer hydrogenation reaction between *N*-benzylidene-*tert*-butylamine (0.3 mmol) and 1.5 equiv. of 1,4-cyclohexadiene using Cs(*t*BuDHP) (5 mol%) as catalyst in THF-d8 showing the formation of the amine product after heating for 1 hour at 70 °C. Adamantane (0.03 mmol) standard has been used to calculate the percentage yield (Entry 16, Yield 99%)

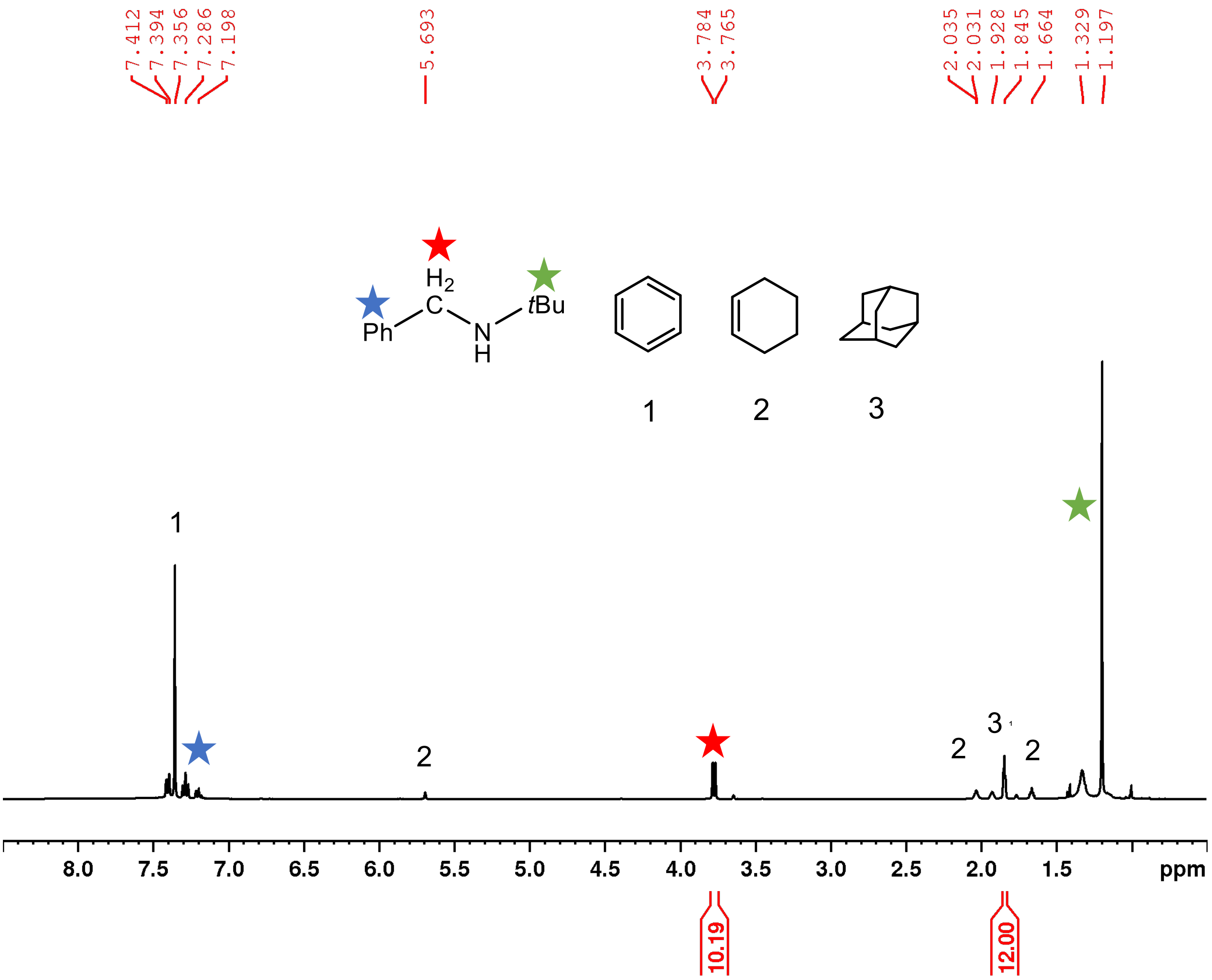

Figure S17 ^{1}H NMR spectrum of the completed transfer hydrogenation reaction between *N*-benzylidene-*tert*-butylamine (0.3 mmol) and 1.5 equiv. of 1,4-cyclohexadiene using Cs(*t*BuDHP) (2.5 mol%) as catalyst in THF-d_8 showing the formation of the amine product after heating for 3 hours at 70 °C. Adamantane (0.0565 mmol) standard has been used to calculate the percentage yield (Entry 17, Yield 95%)

1.3 Benzophenone Imine Catalysis

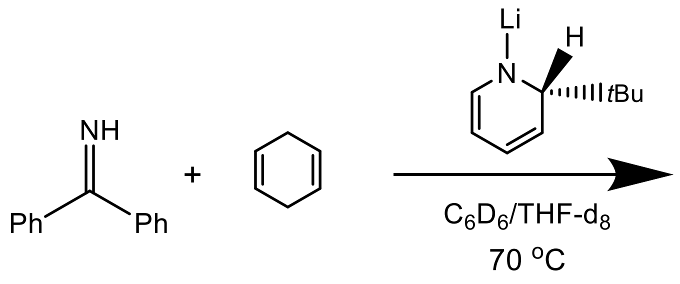

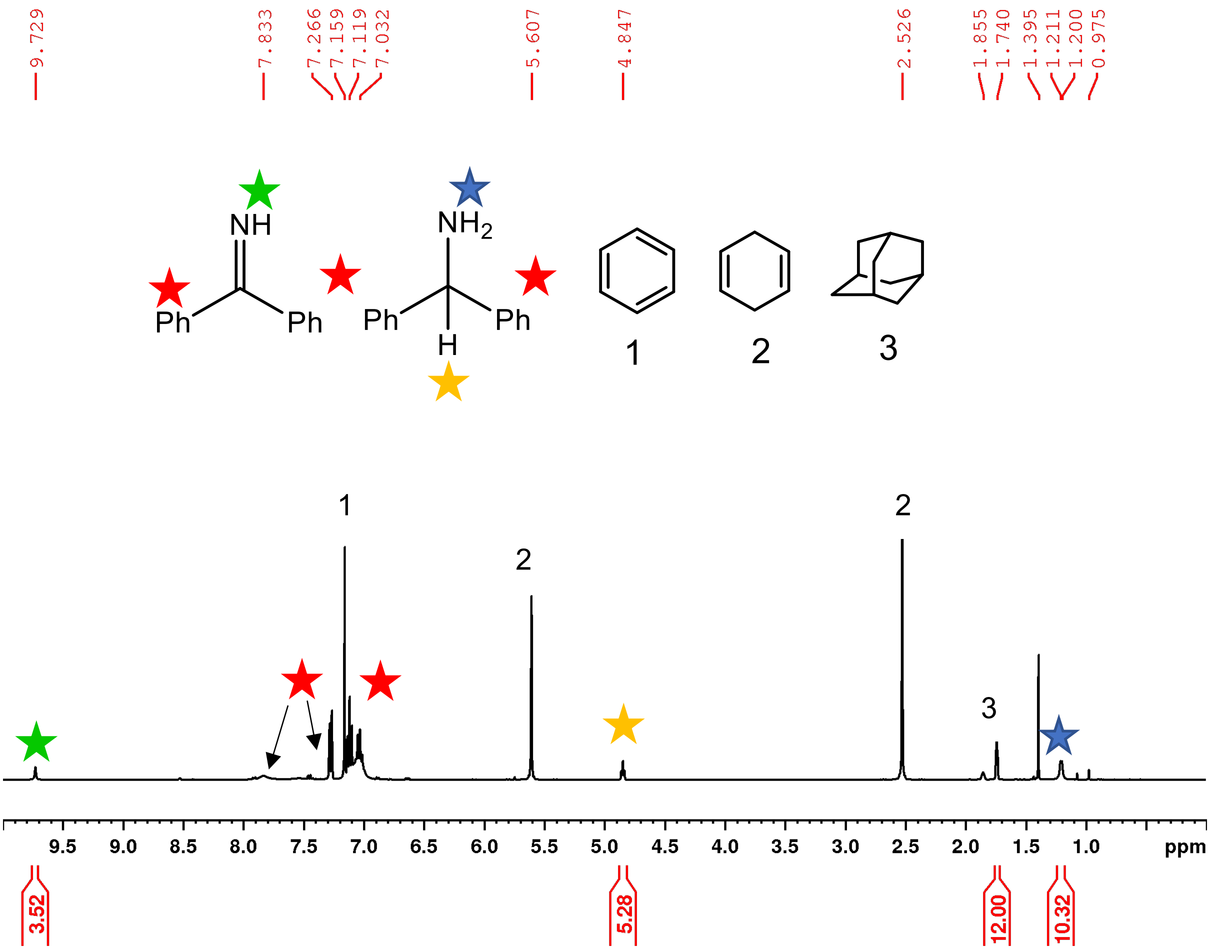

Figure S18 ^{1}H NMR spectrum of the completed transfer hydrogenation reaction between benzophenone imine (0.3 mmol) and Li(*t*BuDHP) (10 mol%) as catalyst using 1.5 equiv. of 1,4-cyclohexadiene in C_6D_6 showing the partial formation of the amine product after heating for 24 hours at 70 °C. Adamantane standard was used (0.033 mmol) to calculate the percentage yield. (Entry 18, Yield 60%)

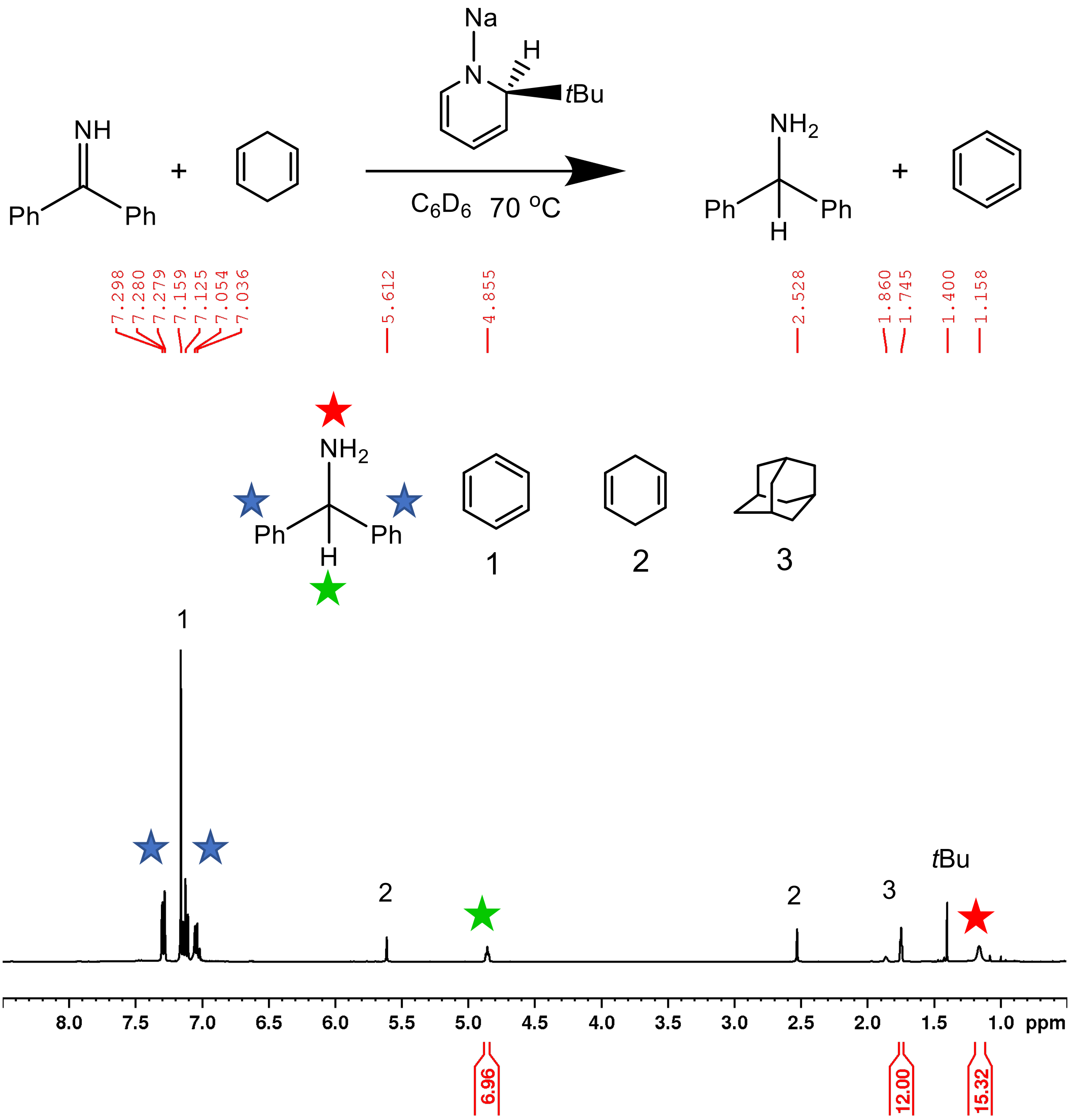

Figure S19 ^{1}H NMR spectrum of the completed transfer hydrogenation reaction between benzophenone imine (0.3 mmol) and 1.5 equiv. of 1,4-cyclohexadiene using Na(*t*BuDHP) (10 mol%) as catalyst in C_6D_6 showing the formation of the amine product after heating for 20 hours at 70 °C. Resonances of isomeric 1,3-cyclohexadiene have also been shown. Adamantane standard was used (0.0375 mmol) to calculate the percentage yield. (Entry 19, Yield 87%)

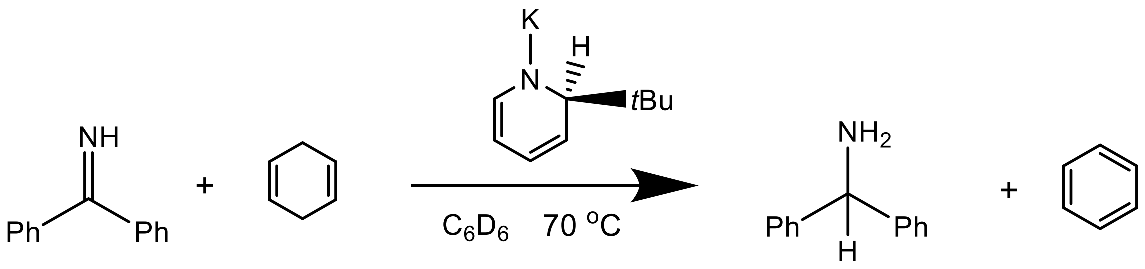

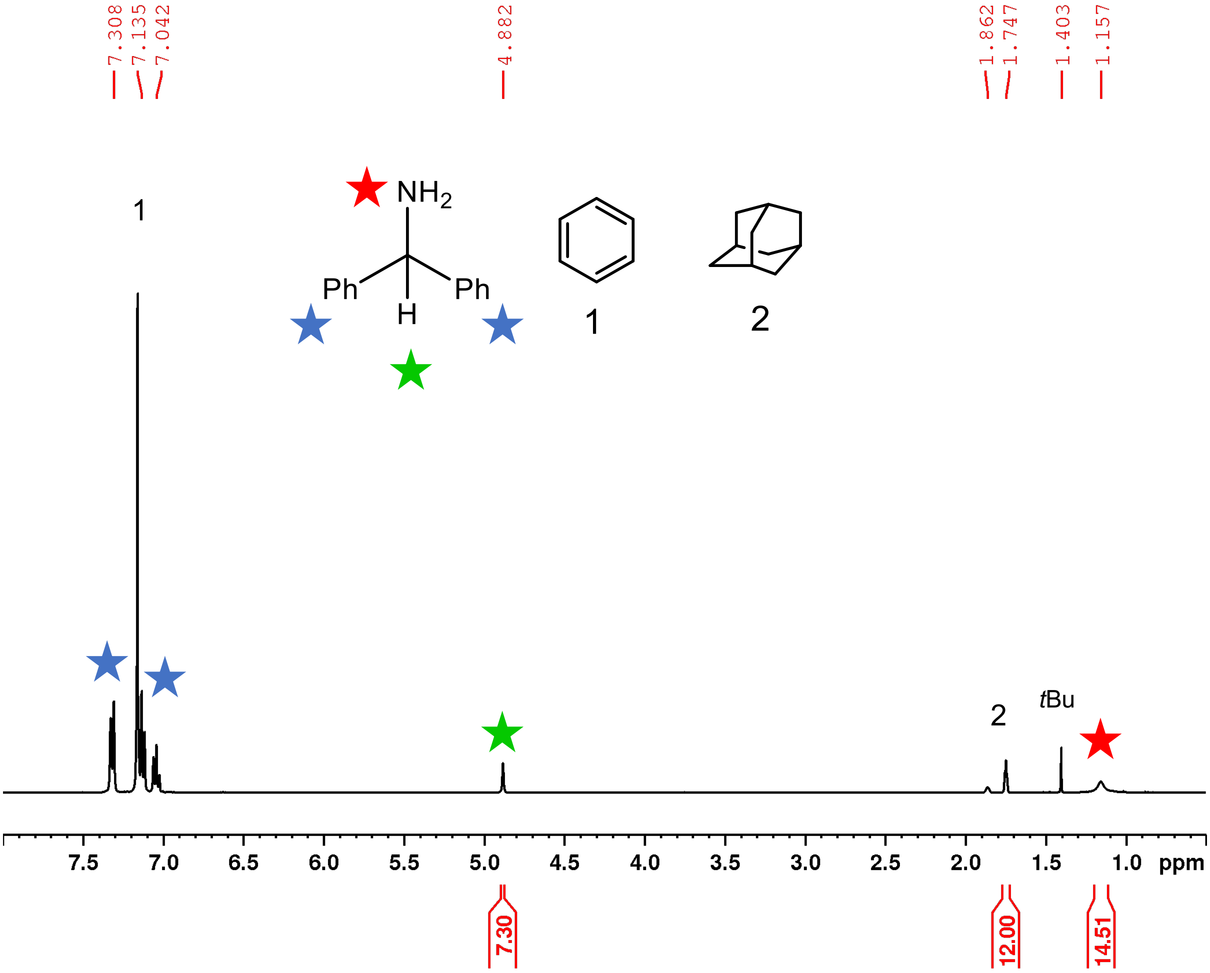

Figure S20 ^{1}H NMR spectrum of the completed transfer hydrogenation reaction between benzophenone imine (0.3 mmol) and 1.5 equiv. of 1,4-cyclohexadiene using K(*t*BuDHP) (10 mol%) as catalyst in C_6D_6 showing the formation of the amine product after heating for 4 hours at 70 °C. Adamantane standard was used (0.0375 mmol) to calculate the percentage yield. (Entry 20, Yield 90%)

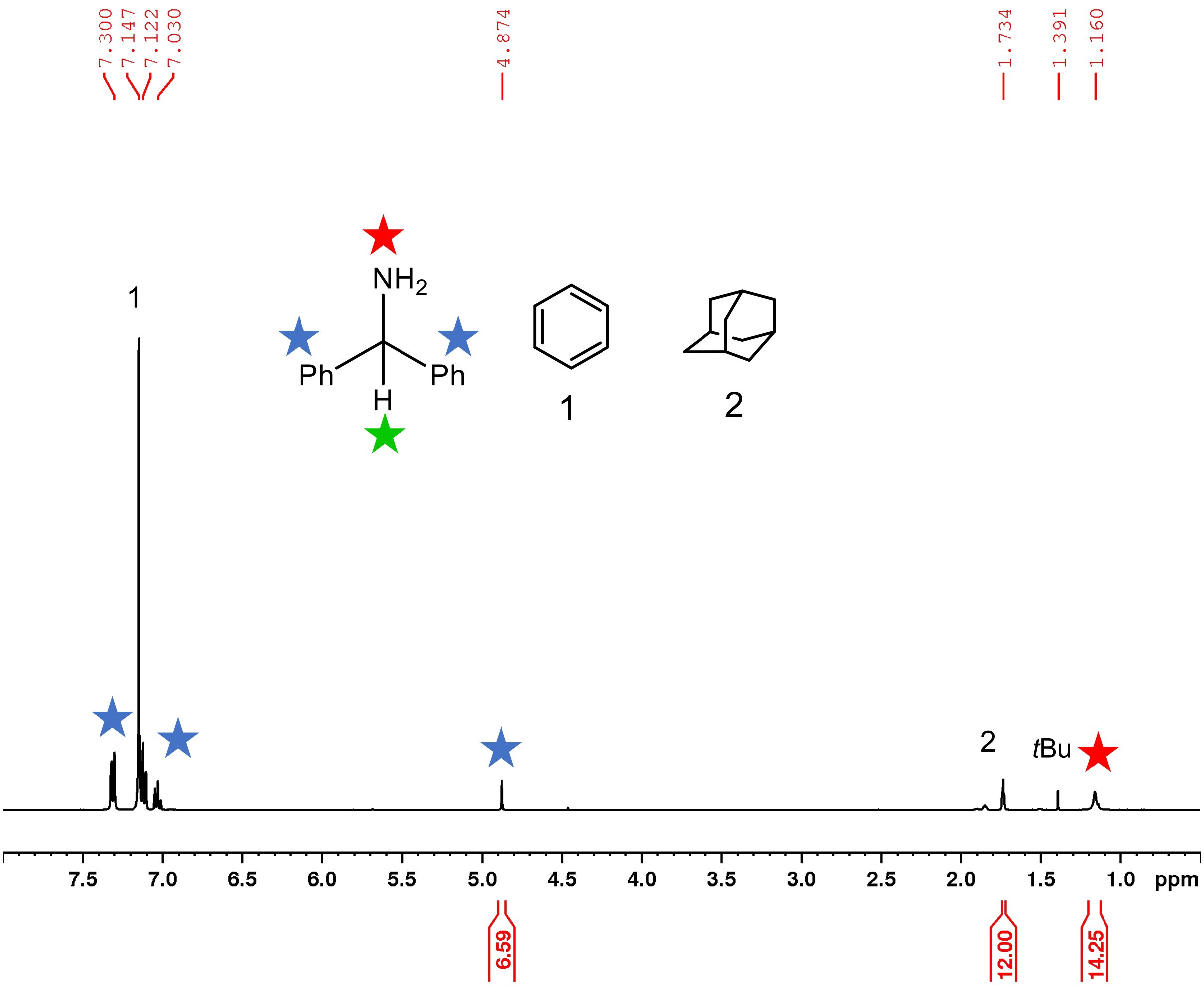

Figure S21 ^{1}H NMR spectrum of the completed transfer hydrogenation reaction between benzophenone imine (0.3 mmol) and 1.5 equiv. of 1,4-cyclohexadiene using Rb(*t*BuDHP) (10 mol%) as catalyst in C_6D_6 showing the formation of the amine product after heating for 1 hour at 70 °C. Adamantane standard was used (0.0425 mmol) to calculate the percentage yield. (Entry 21, Yield 94%)

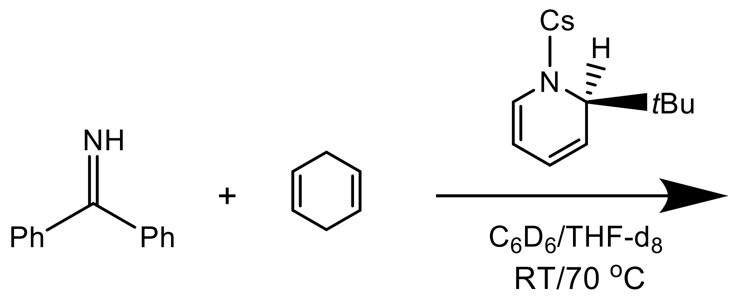

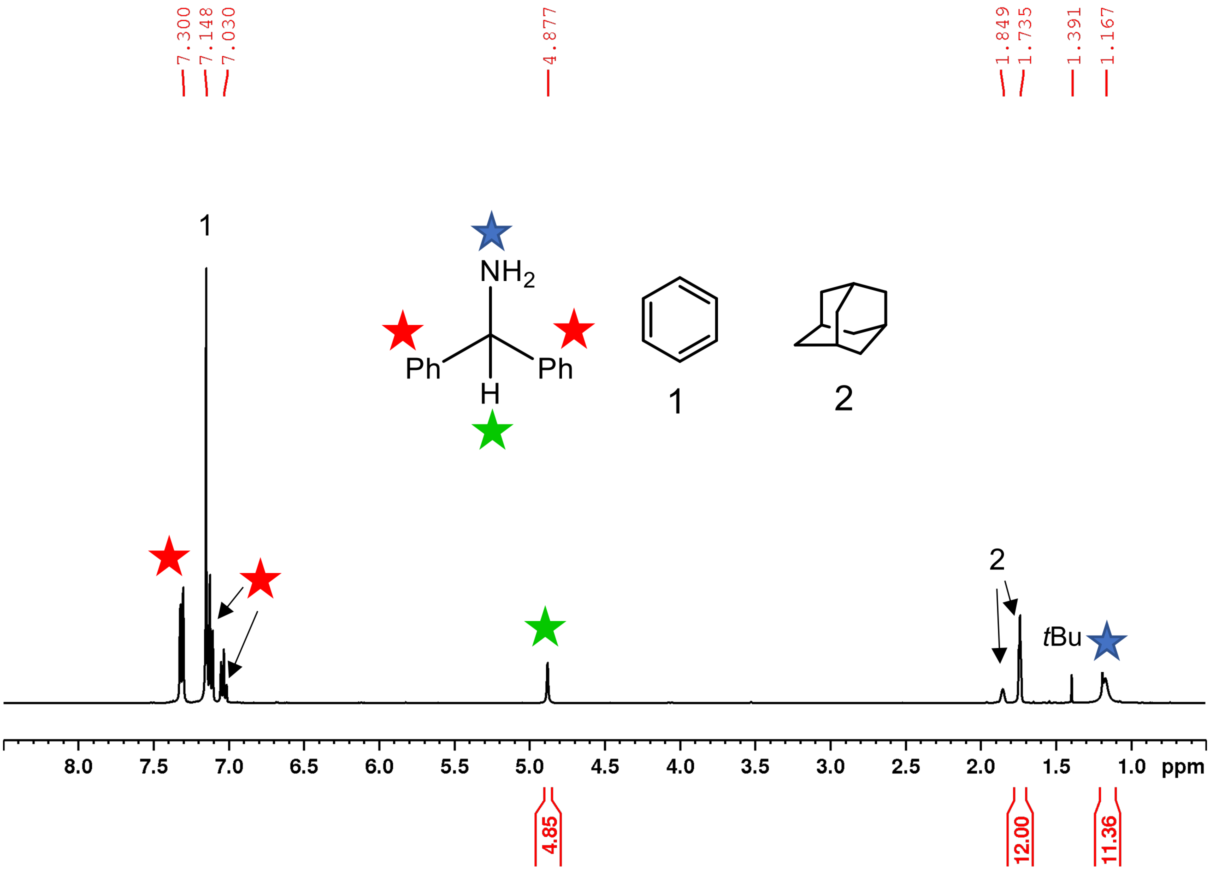

Figure S22 ^{1}H NMR spectrum of the completed transfer hydrogenation reaction between benzophenone imine (0.3 mmol) and 1.5 equiv. of 1,4-cyclohexadiene using Cs(*t*BuDHP) (10 mol%) as catalyst in C_6D_6 showing the formation of the amine product after heating for 0.25 hours at 70 °C. Adamantane standard was used (0.061 mmol) to calculate the percentage yield. (Entry 22, Yield 99%)

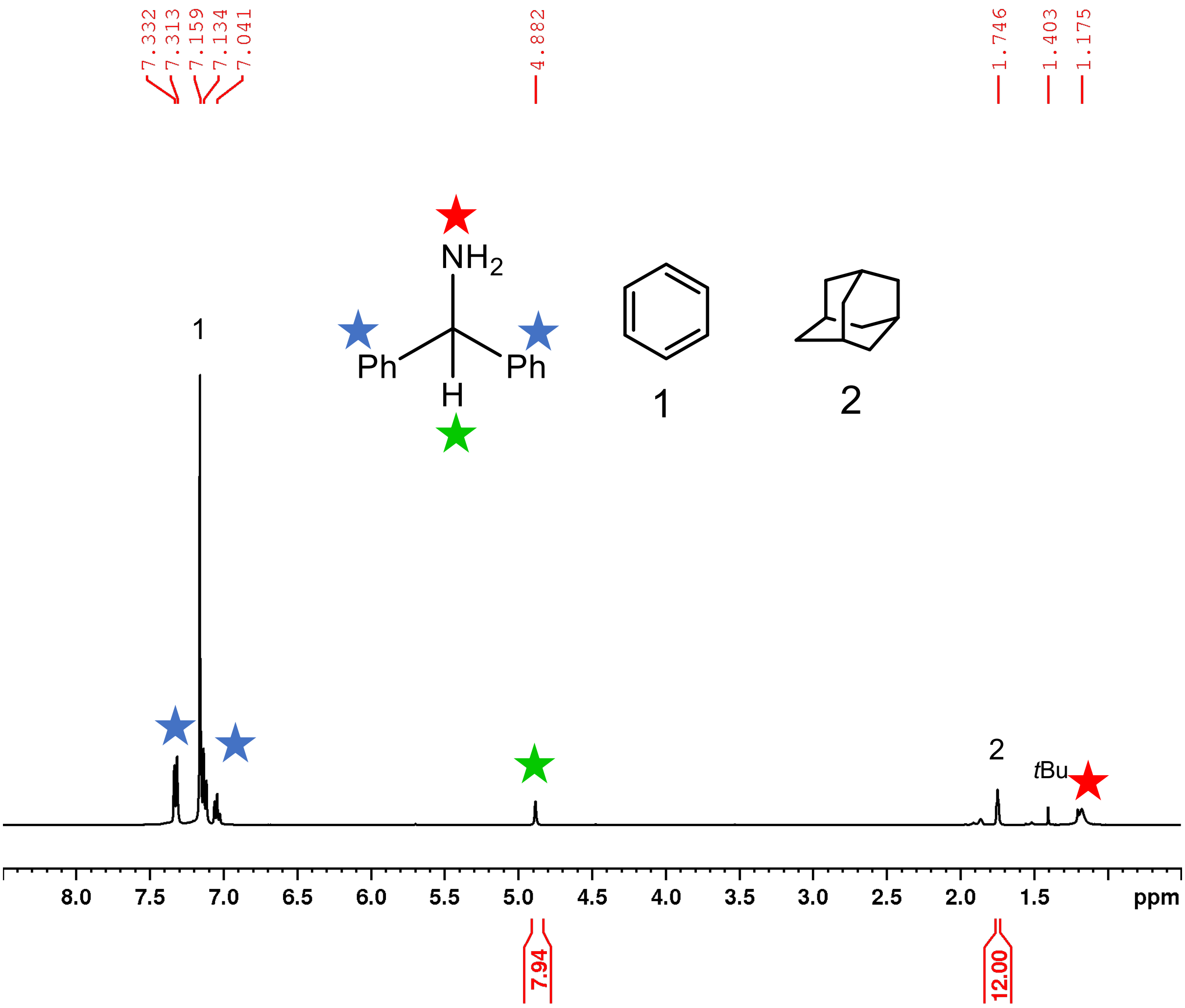

Figure S23 ^{1}H NMR spectrum of the completed transfer hydrogenation reaction between benzophenone imine (0.3 mmol) and 1.5 equiv. of 1,4-cyclohexadiene using Cs(*t*BuDHP) (5 mol%) as catalyst in C_6D_6 showing the formation of the amine product after heating for 0.5 hours at 70 °C. Adamantane (0.0375 mmol) standard has been used to calculate the percentage yield. (Entry 23, Yield = 99%)

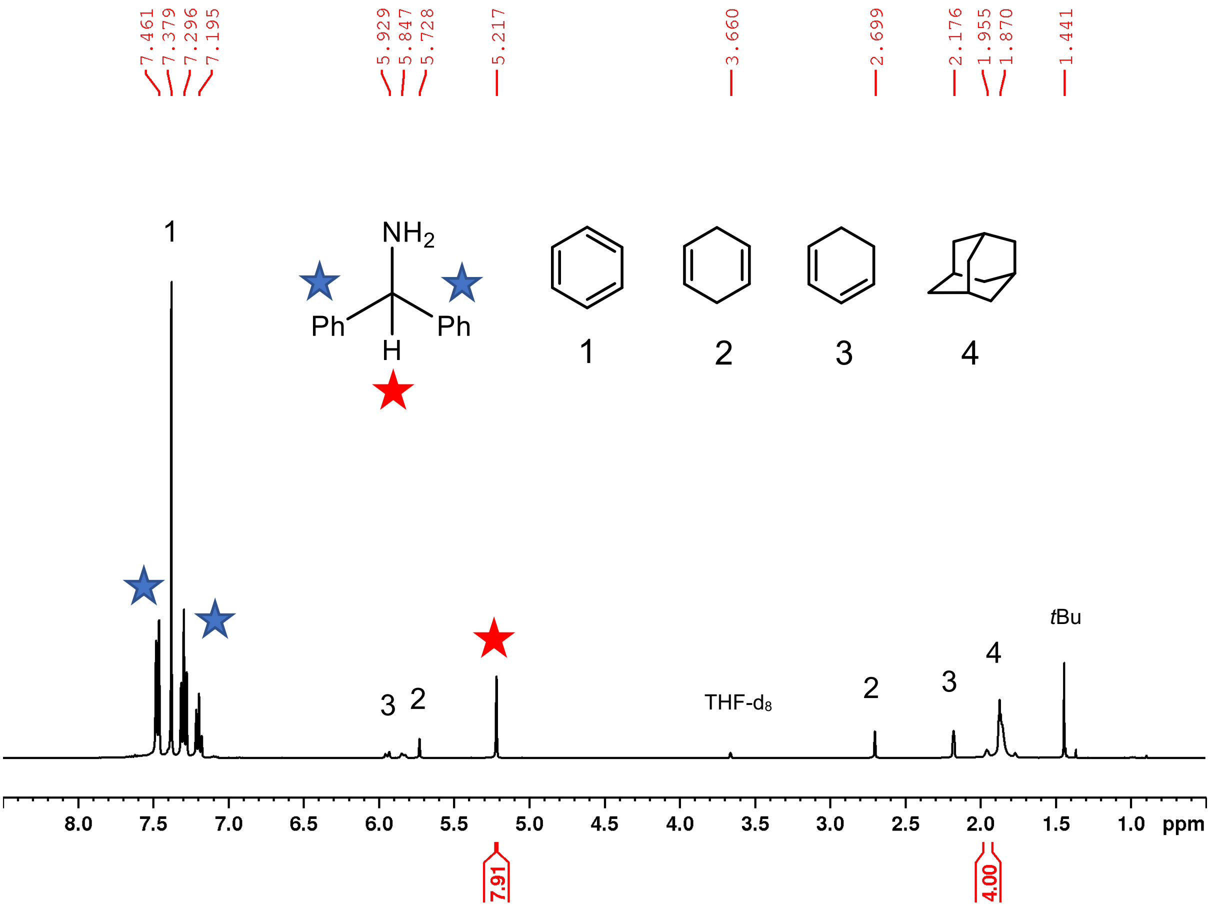

Figure S24 ^{1}H NMR spectrum of the completed transfer hydrogenation reaction between benzophenone imine (0.3 mmol) and Li(*t*BuDHP) (10 mol%) as catalyst using 1.5 equiv. of 1,4-cyclohexadiene in THF-d_8 showing the partial formation of the amine product after heating for 14 hours at 70 °C. Resonances of isomeric 1,3-cyclohexadiene have also been shown 1,3-cyclohexadiene have also been shown. Adamantane standard was used (0.03 mmol) to calculate the percentage yield with the smaller peak being integrated as 4 due to the overlap of the larger adamantane peak with that of the NH_2 product peak. (Entry 24, Yield 79%)

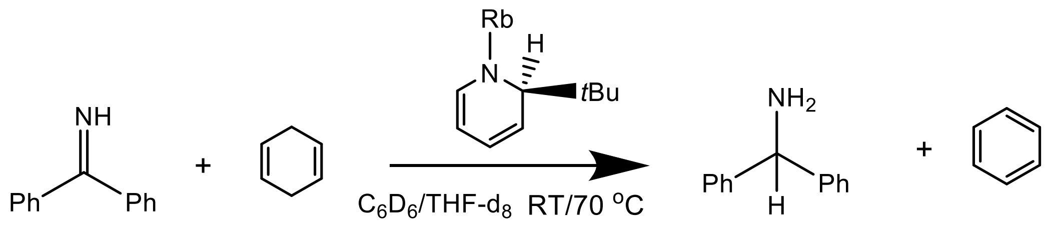

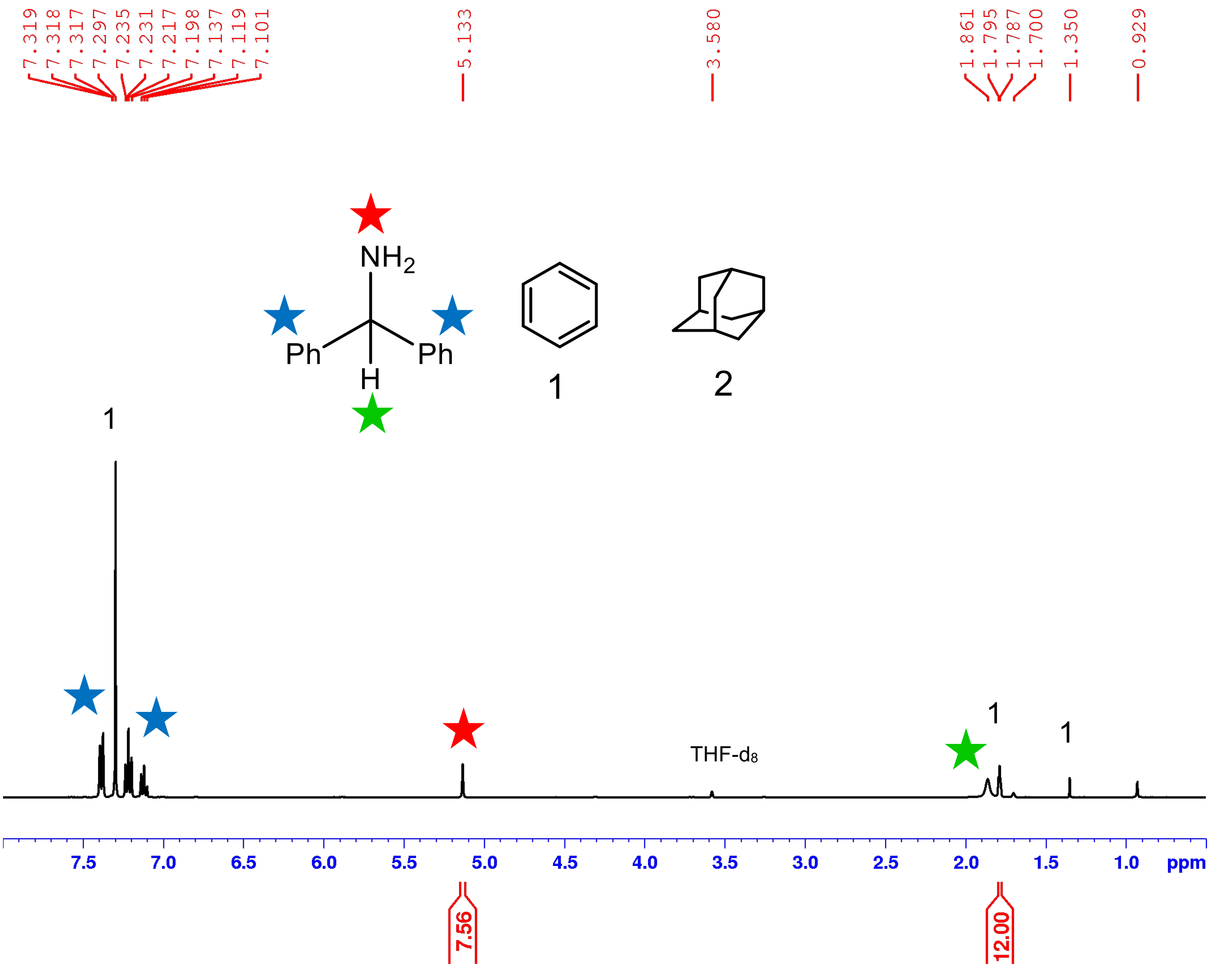

Figure S25 ^{1}H NMR spectrum of the completed transfer hydrogenation reaction between benzophenone imine (0.3 mmol) and 1.5 equiv. of 1,4-cyclohexadiene using Rb(*t*BuDHP) (10 mol%) as catalyst in THF-d_8 showing the formation of the amine product after heating for 0.5 hours at 70 °C. Adamantane standard was used (0.0375 mmol) to calculate the percentage yield. (Entry 25, Yield 95%)

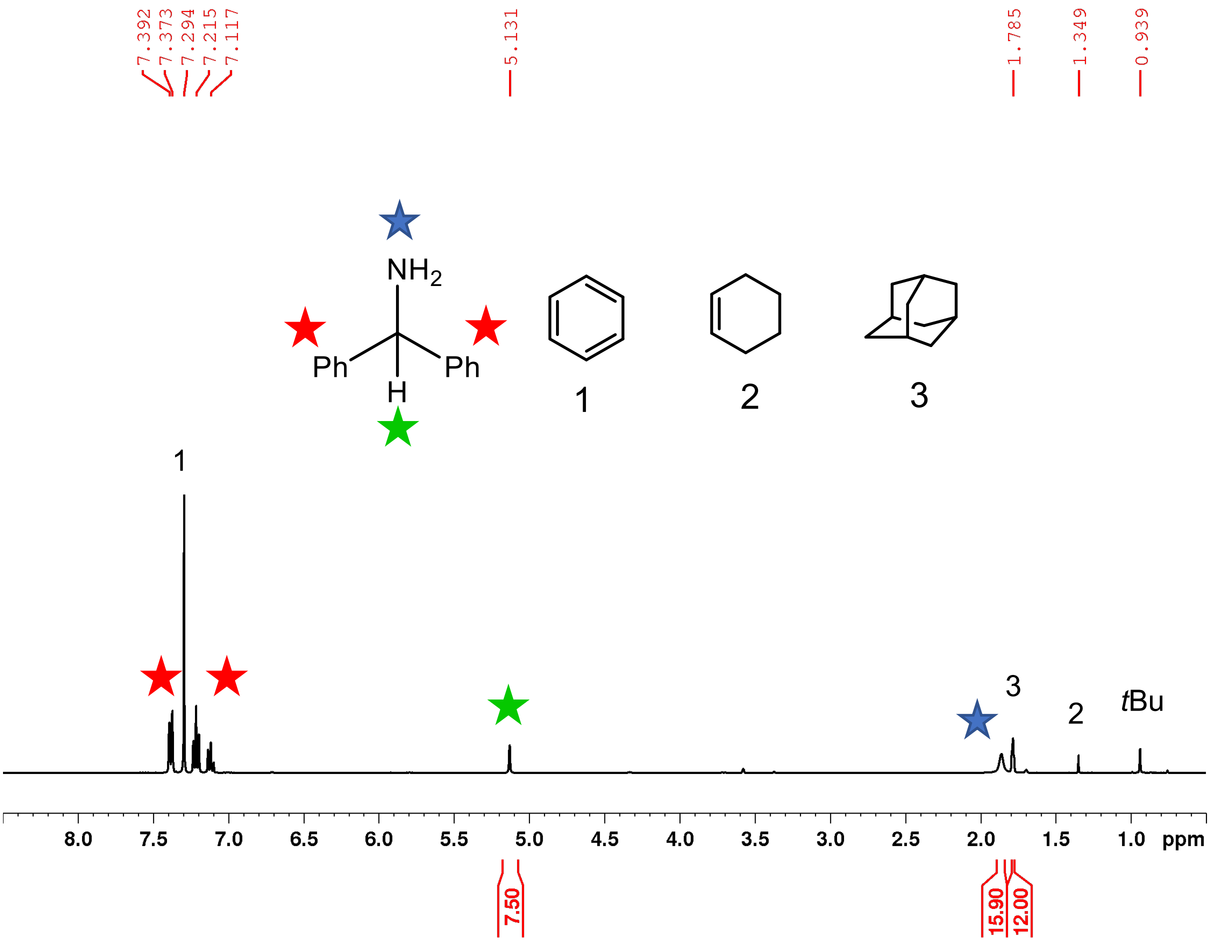

Figure S26 ^{1}H NMR spectrum of the completed transfer hydrogenation reaction between benzophenone imine (0.3 mmol) and 1.5 equiv. of 1,4-cyclohexadiene using Cs(*t*BuDHP) (10 mol%) as catalyst in THF-d_8 showing the formation of the amine product after 0.25 hours at room temperature. Resonances of cyclohexene have also been shown. Adamantane (0.0375 mmol) standard was used to calculate the percentage yield. (Entry 26, Yield 97%)

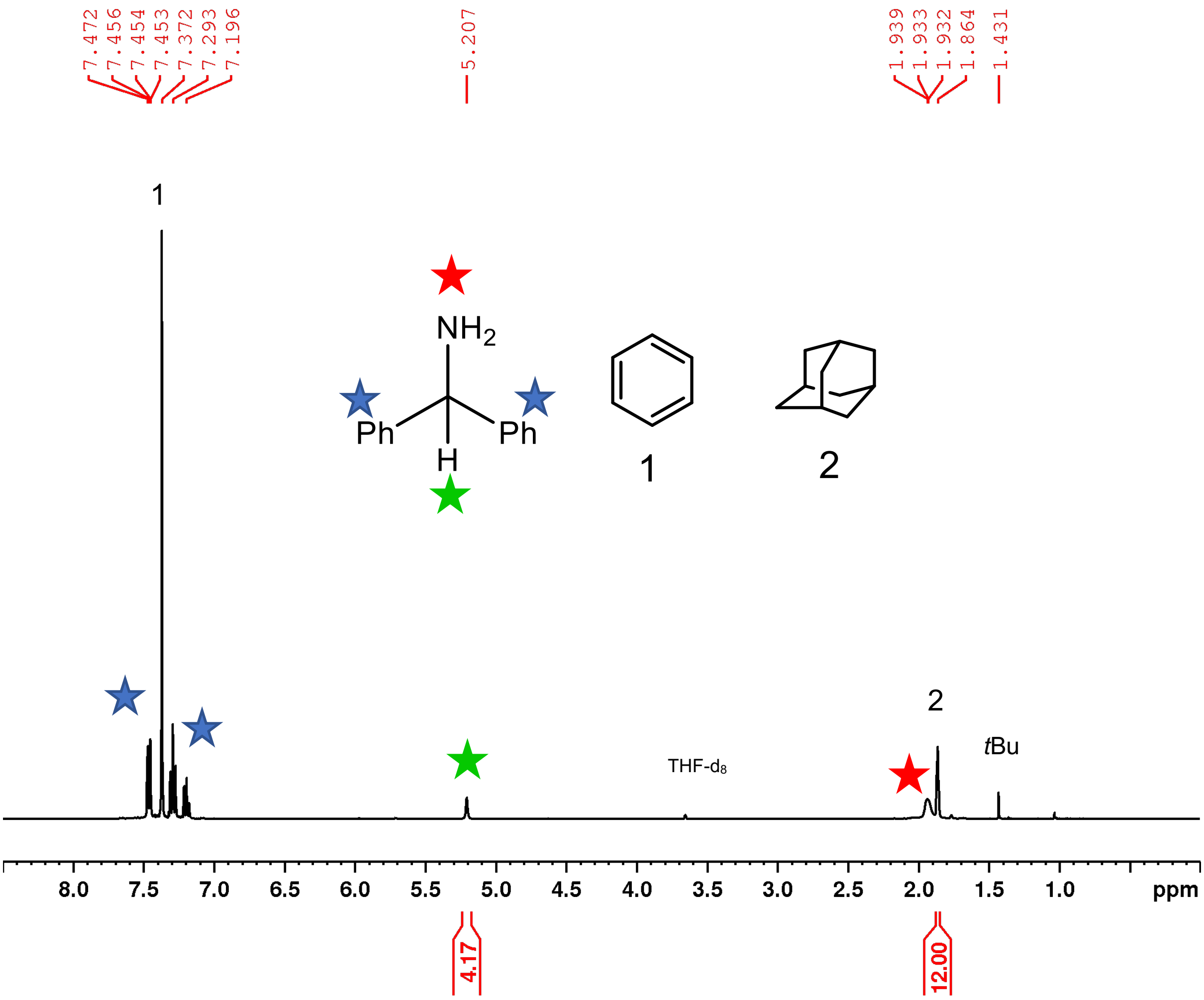

Figure S27 ^{1}H NMR spectrum of the completed transfer hydrogenation reaction between benzophenone imine (0.3 mmol) and 1.5 equiv. of 1,4-cyclohexadiene using Cs(*t*BuDHP) (5 mol%) as catalyst in THF-d_8 showing the formation of the amine product after heating for less than 0.25 hours at 70 °C. Adamantane (0.071 mmol) standard has been used to calculate the percentage yield (Entry 27, Yield 99%)

2 Catalyst activity

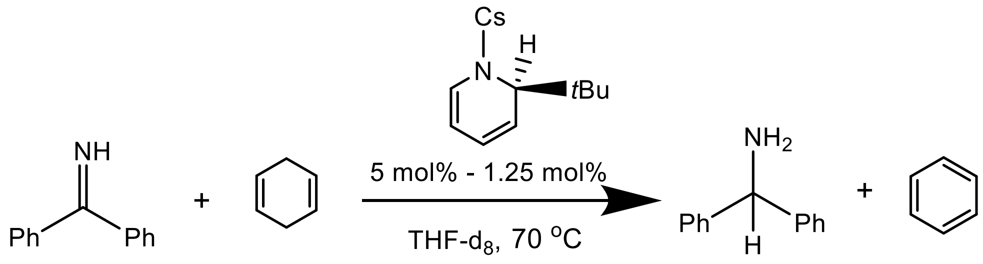

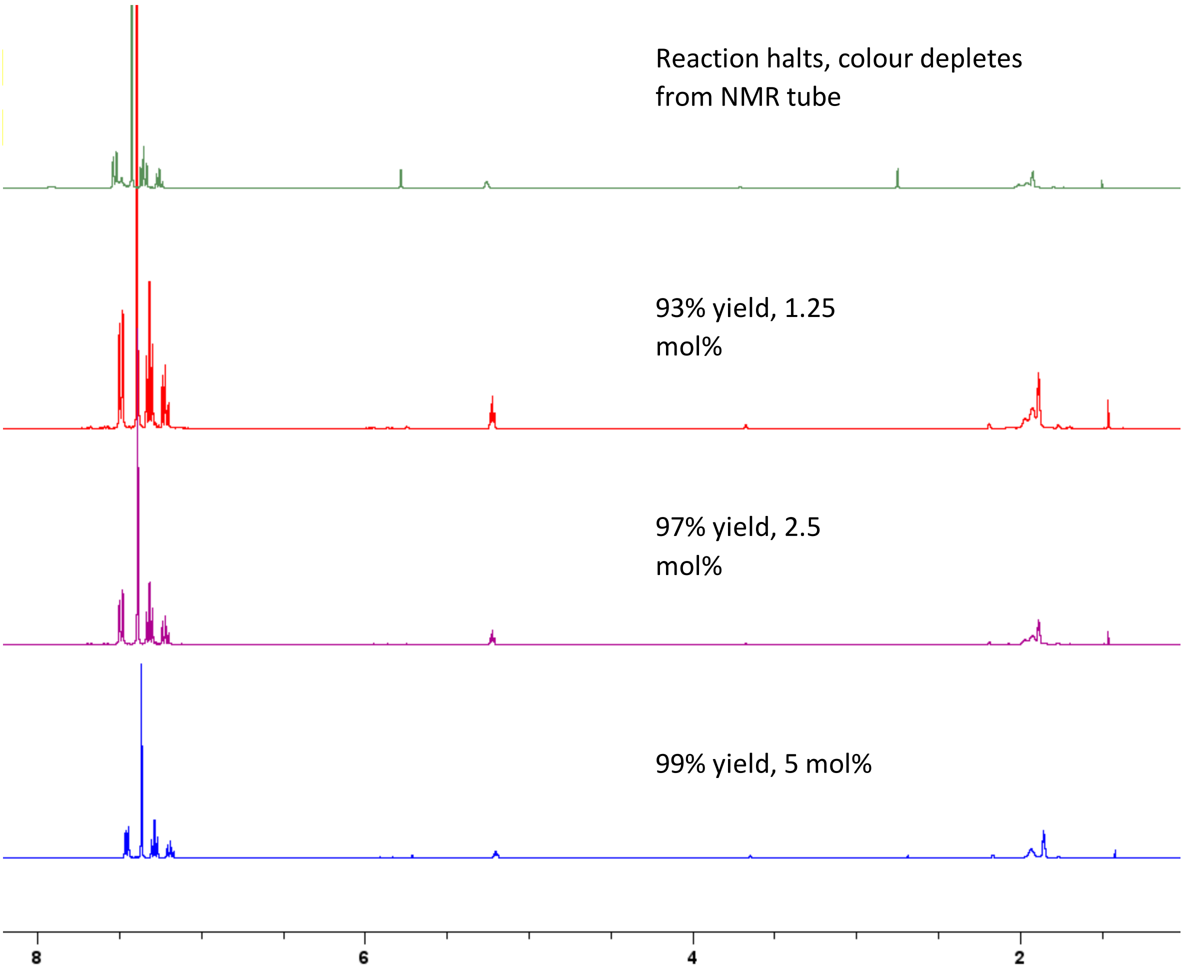

Figure S28 Completed reaction between benzophenone imine and 1,4-cyclohexadiene using Cs(*t*BuDHP) (5 mol%, blue NMR spectrum) as catalyst. Purple and red NMR spectra represent the additional equivalents of imine and 1,4-cyclochexadiene once respective catalytic reactions had stopped. Green NMR spectrum shows the reaction ceasing once catalytic loading has become so low.

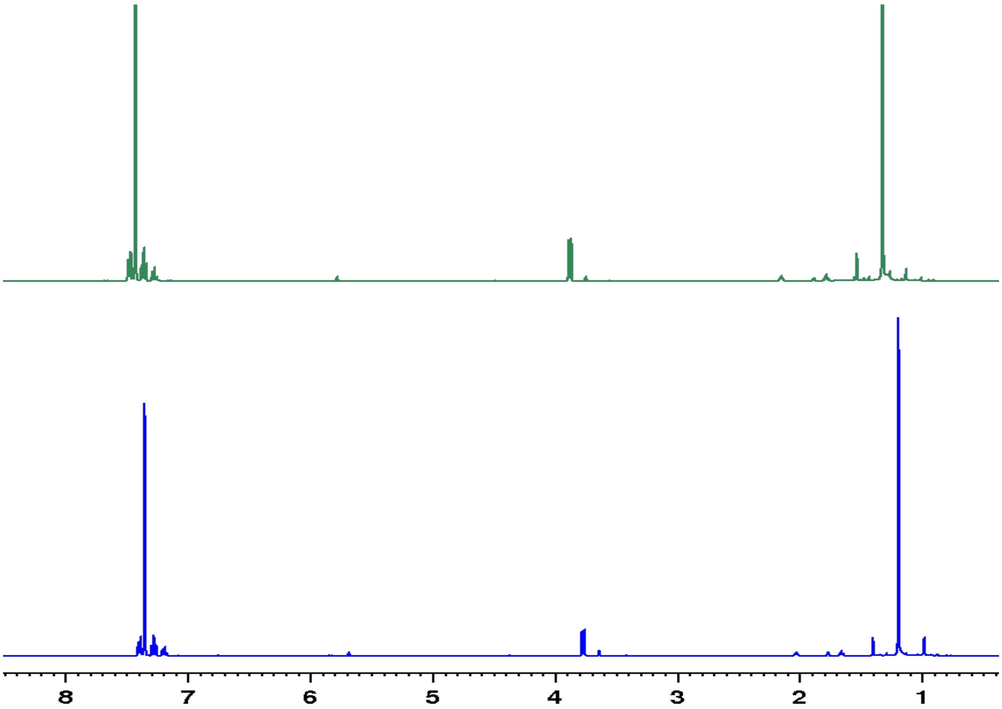

Figure S29 Top NMR spectrum features original transfer hydrogenation reaction between *N*-benzylidene-*tert*-butylamine and 1,4-cyclohexadiene, using Cs(*t*BuDHP) (5 mol%) as the catalyst in THF-d_8 (99%, 0.25 hours). Bottom NMR spectrum features the same reaction cycle **one week later** where another equiv. of 1,4-cyclohexadiene and benzophenone imine has been added (2.5 mol%, 95%, 0.5 hours)

3 Stoichiometric Reactions

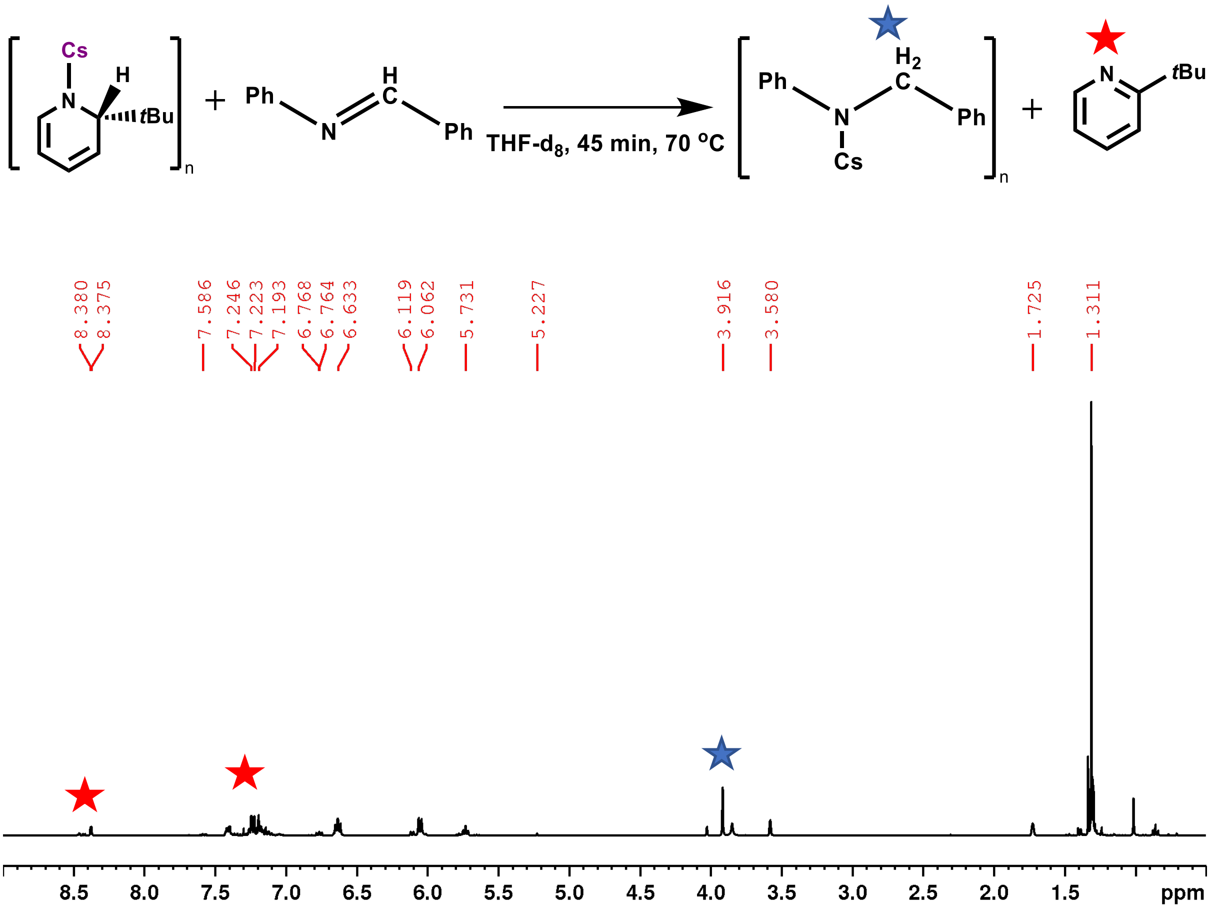

Figure S30 Stoichiometric reaction between Cs(*t*BuDHP) and *N*-benzylideneaniline to form the corresponding Cs amide compound (**2**) with its characteristic CH_2 peak at 3.92 ppm along with the by-product 2-*tert*-butylpyridine (aromatics)

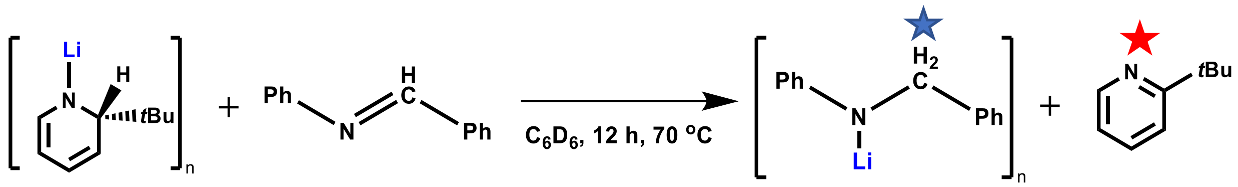

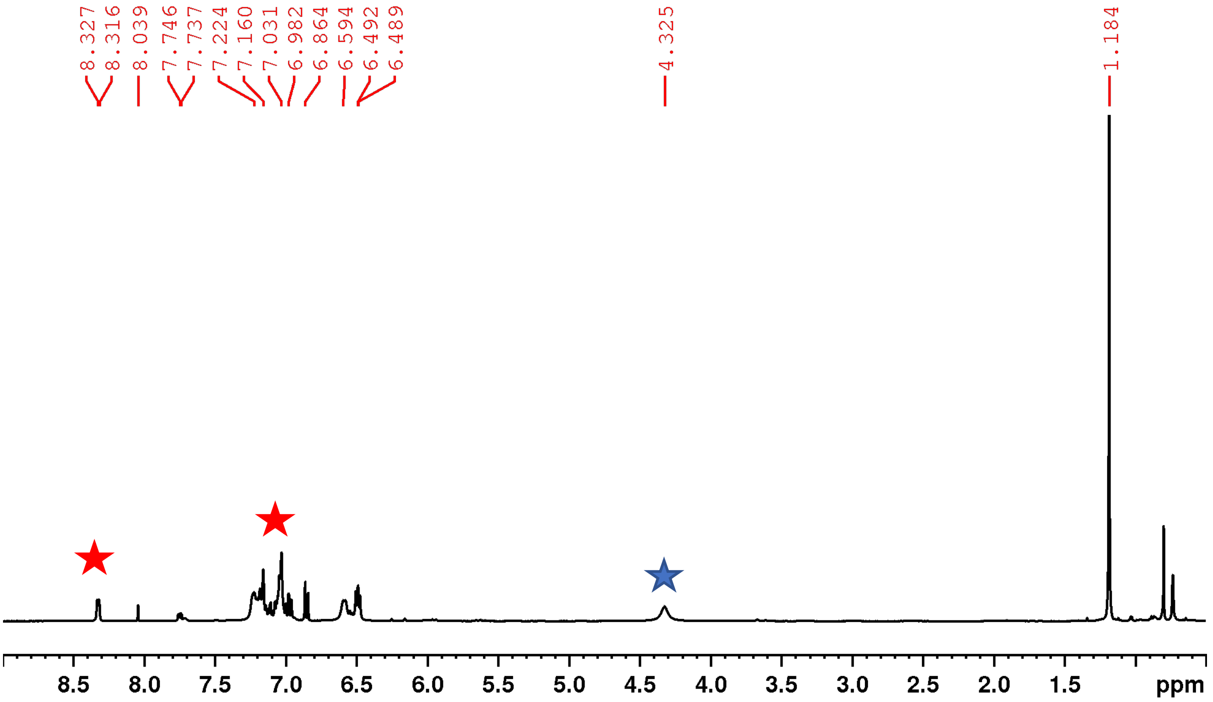

Figure S31 Stoichiometric reaction between Li(*t*BuDHP*)* and *N*-benzylideneaniline to form the corresponding Li amide compound (**1**) with its characteristic C**H**$_2$ peak at 4.33 ppm along with the by-product 2-*tert*-butylpyridine (aromatics)

4 Crystal Structures

4.1 $[PhCH_2N(Li)Ph]_\infty$ (**1**)

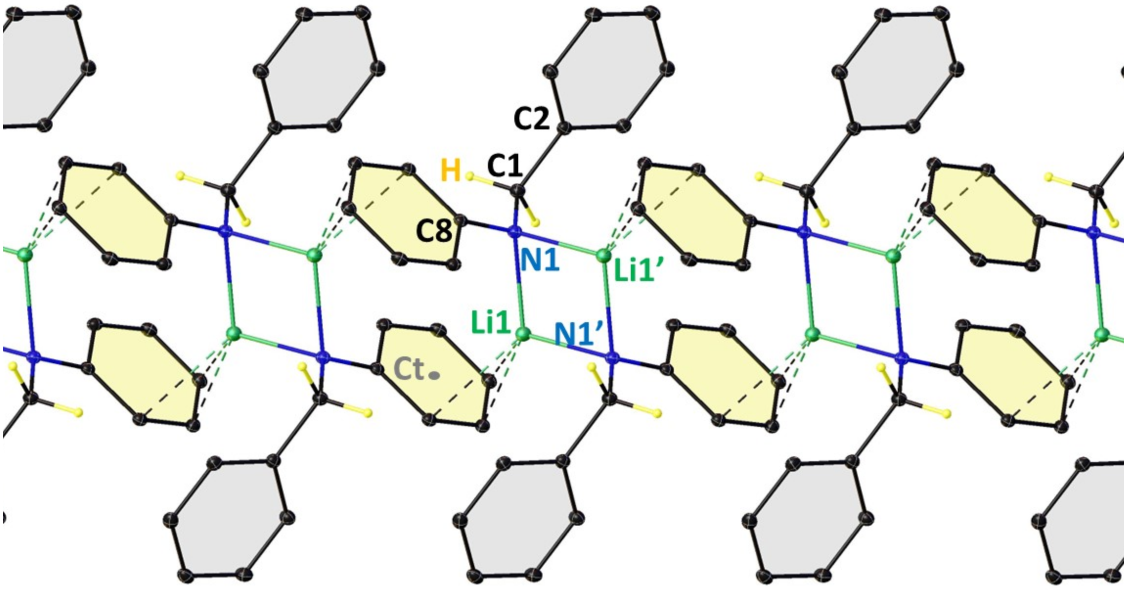

Figure S32 Section of polymeric structure of $[PhCH_2N(Li)Ph]_\infty$ (**1**). Thermal ellipsoids displayed at 30% probability. All H atoms except Ph-N(Li)C(*H*)$_2$-Ph have been omitted for clarity.

4.2 $[PhCH_2N(Cs)Ph]_\infty$ (**2**)

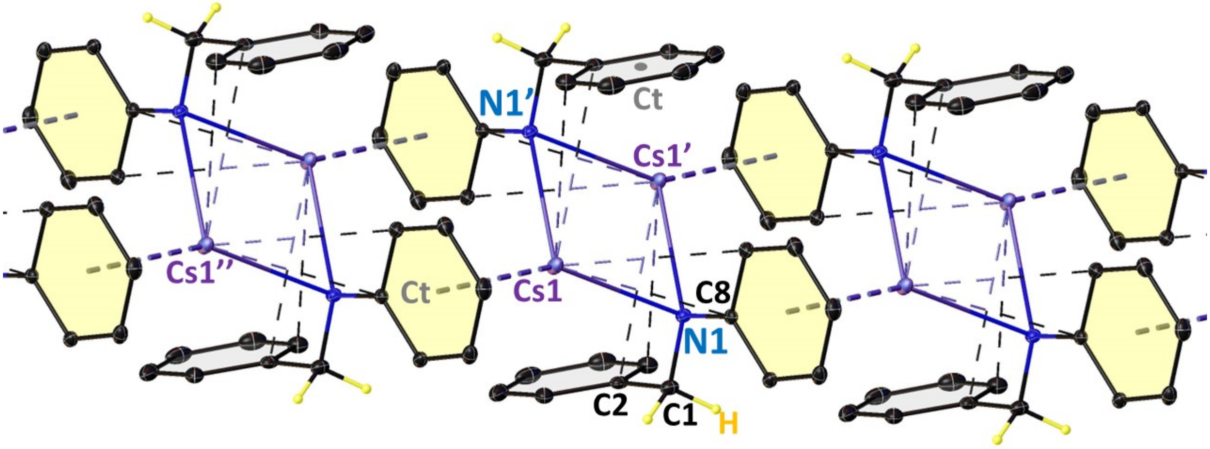

Figure S33 Section of polymeric structure of $[PhCH_2N(Cs)Ph]_\infty$ (**1**). Thermal ellipsoids displayed at 30% probability. All H atoms except Ph-N(Cs)C(*H*)$_2$-Ph have been omitted for clarity.

Table S1 Selected bond distances (Å) and angles (°) of **1** and **2**

	Li-amide **(1)**	Cs-amide **(2)**
Interaction	Bond lengths (Å)	
AM1-AM1’	2.524(5)	4.4399(3)
AM1-N1	2.019(3)	3.0950(15)
AM1’-N1	2.032(3)	3.1200(15)
AM1-$Ct_{(yellow\ plane)}$	2.3702(7)	3.2175(8)
AM1-$Ct_{(grey\ plane)}$	-	4.0320(8)
AM1’’-$Ct_{(yellow\ plane)}$	-	4.0482(9)
N1-C1	1.4742(17)	1.455(2)
N1-C8	1.3658(16)	1.348(2)
C1-C2	1.5221(18)	1.502(3)
Interaction	Bond angles (°)	
AM1-N1-Cs1’	77.08(11)	91.19(4)
N1-AM1-N1’	102.92(11)	88.81(4)
C2-C1-N1-C8	98.37(13)	177.30(16)

5 Investigation of Mechanism

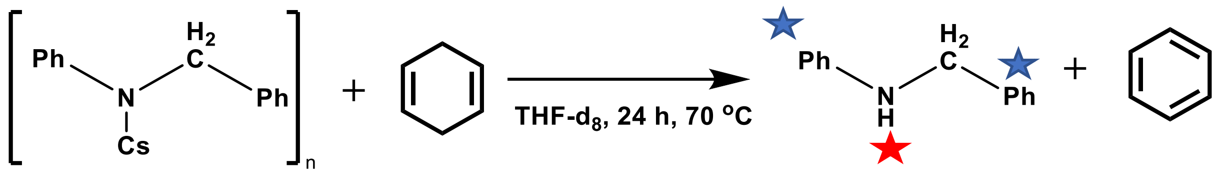

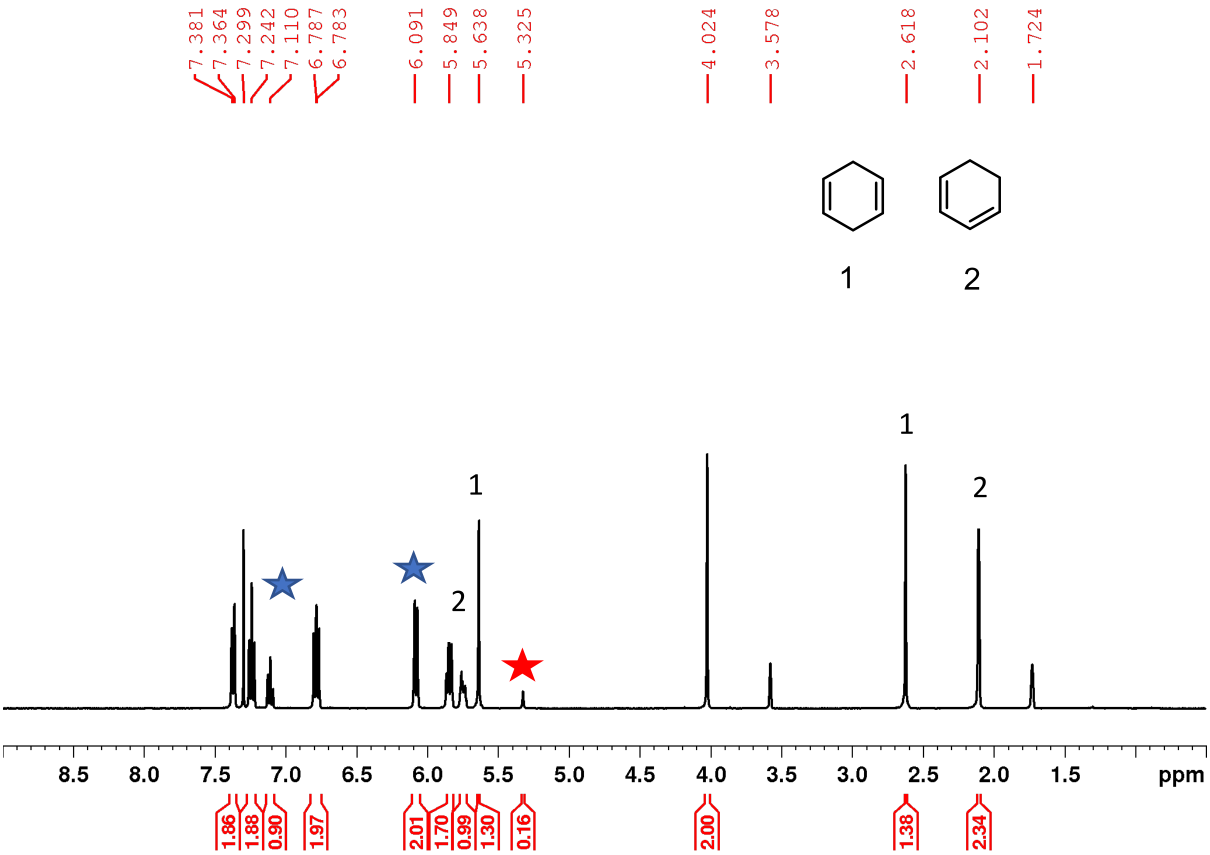

Figure S34 ^{1}H NMR spectrum of stoichiometric reaction between Cs benzyl amide compound (**2**) and 1,4-cyclohexadiene

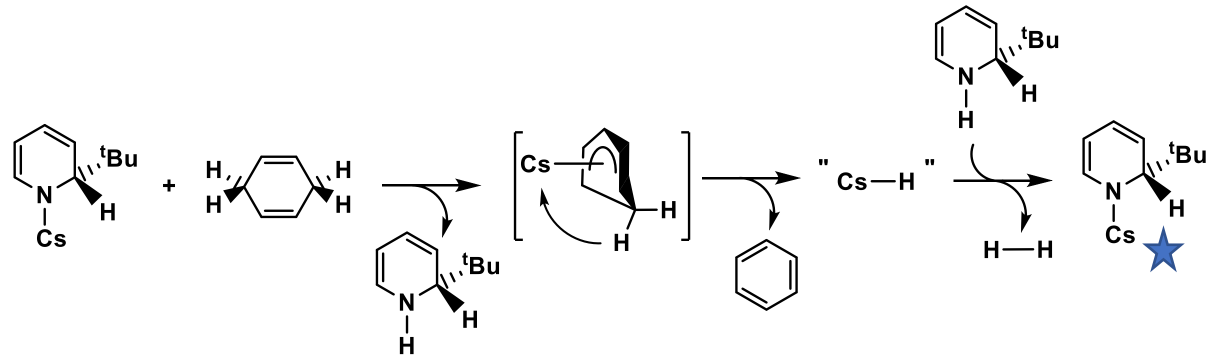

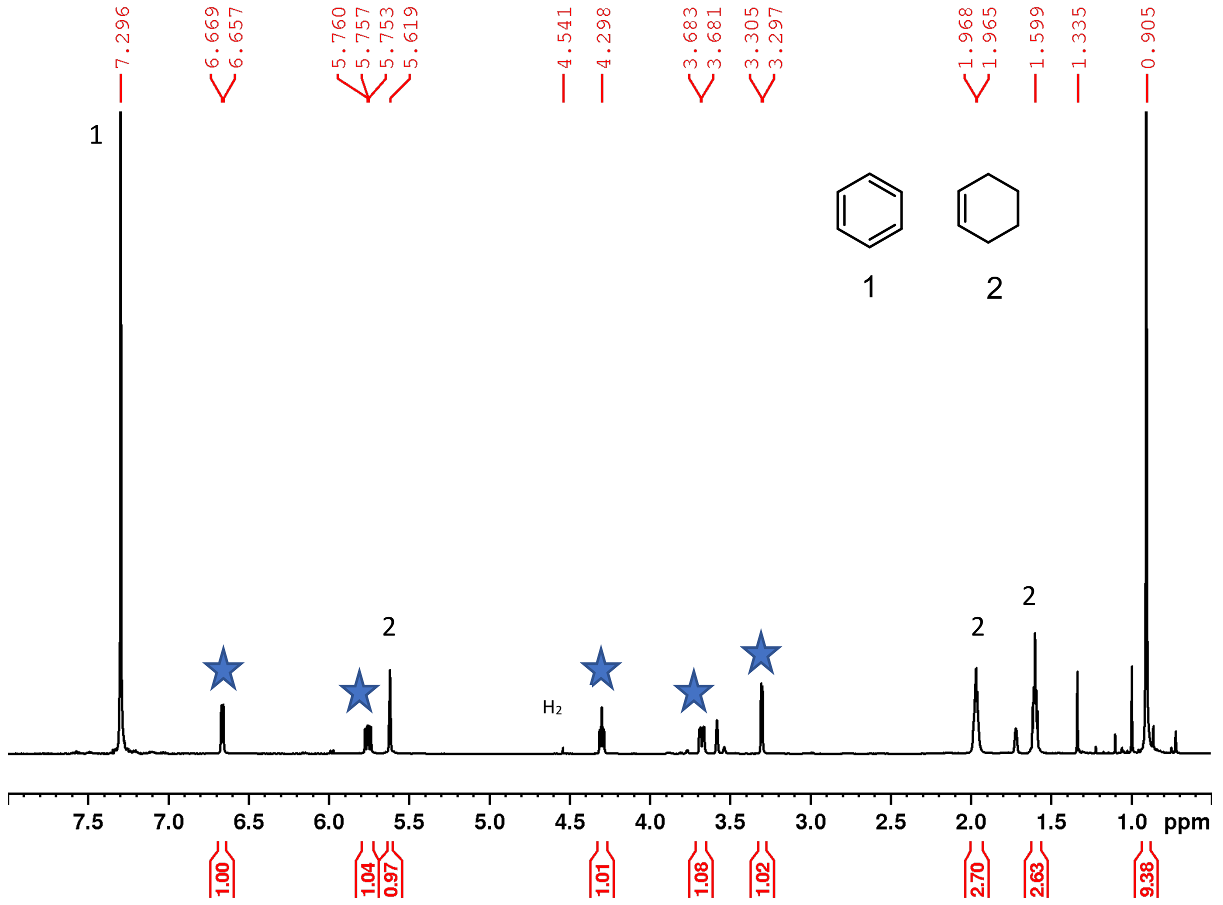

Figure S35 ^{1}H NMR spectrum of stoichiometric reaction between Cs(*t*BuDHP) and 1,4-cyclohexadiene

6 {[Cs(*t*BuDHP)]$_2$·py}$_\infty$ (3): Solid-state structure

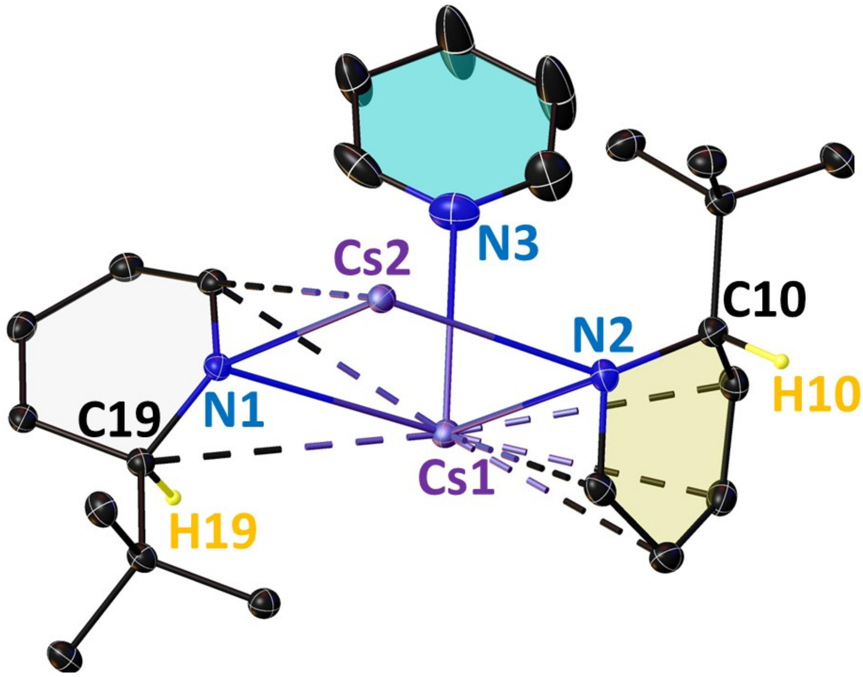

Figure S36 Asymmetric unit of {[Cs(*t*BuDHP)]$_2$·py}$_\infty$ (**3**). Thermal ellipsoids of all atoms displayed at 30% probability. H atoms except the sp^3 hybridized CH on the DHP ring have been removed for clarity.

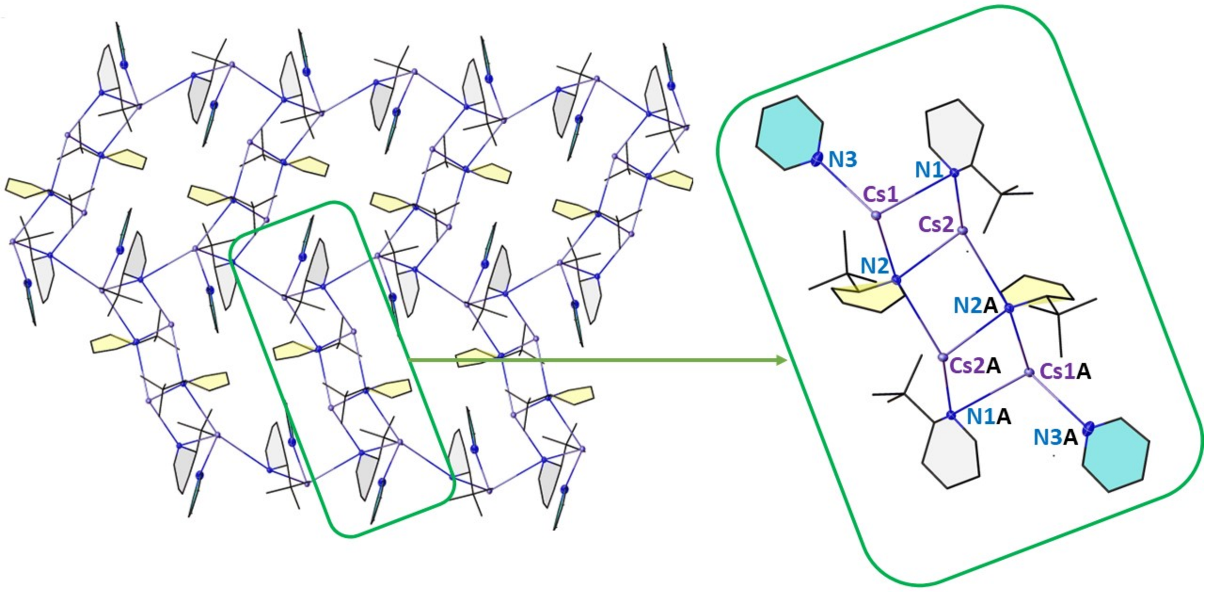

Figure S37 Section of the polymeric {[Cs(*t*BuDHP)]$_2$·py}$_\infty$ (**3**). Thermal ellipsoids of all non-C atoms displayed at 30% probability whereas C atoms are at 5% probability. H atoms and Cs---π interactions have been removed for clarity. Symmetry operator to generate atoms labelled A = 1-x, 1-y, 1-z

7 [Li(*t*BuDHP)·(py)$_3$] (4): Solid-state structure

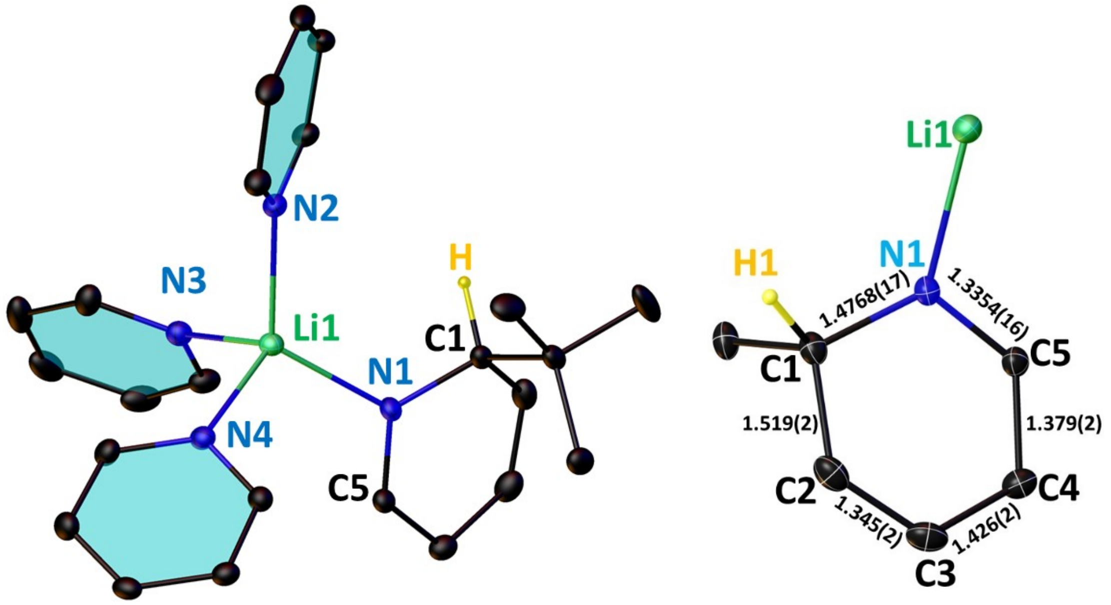

Figure S38 Molecular structure of [Li(*t*BuDHP)]·(py)$_3$] (**4**). Thermal ellipsoids of all atoms displayed at 30% probability. H atoms except the sp^3 hybridized CH on the DHP ring have been removed for clarity.

Table S2 Selected bond distances (Å) and angles (°) of **3** and **4**

	**3**	**4**
Interaction	Bond lengths (Å)	
AM1-N1(DHP)	3.344(4)	1.962(2)
AM1-N2(Py)	-	2.055(2)
AM1-N3(Py)	3.177(7)	2.090(3)
AM1-N4(Py)	-	2.074(2)
AM2-N1(DHP)	3.118(4)	-
AM2-N2(DHP)	3.078(4)	-
AM1-N2(DHP)	3.444(4)	-
AM1-AM2	4.5434(4)	-
Interaction	Bond angles (°)	
N1-AM1-N4	-	108.14(12)
N4-AM1-N3	-	105.74(9)
N3-AM1-N2	107.28(15)	110.38(12)
AM1-N1-AM2	89.28(8)	-
N1-AM2-N2	96.41(10)	-
AM2-N2-AM1	88.13(9)	-
N2-AM1-N1	85.74(9)	-
N1-AM1-N3	99.13(13)	

8 Experiments supporting proposed mechanistic cycle

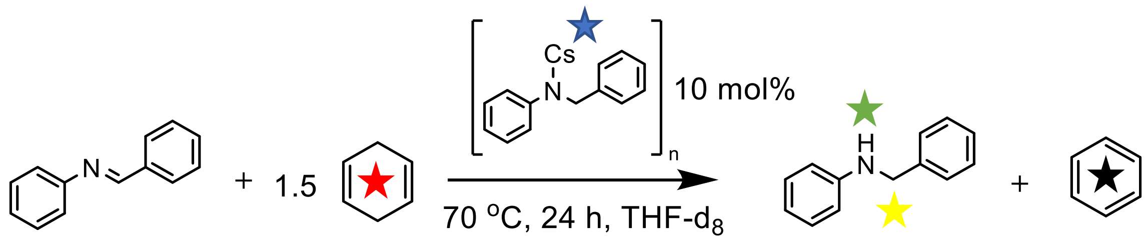

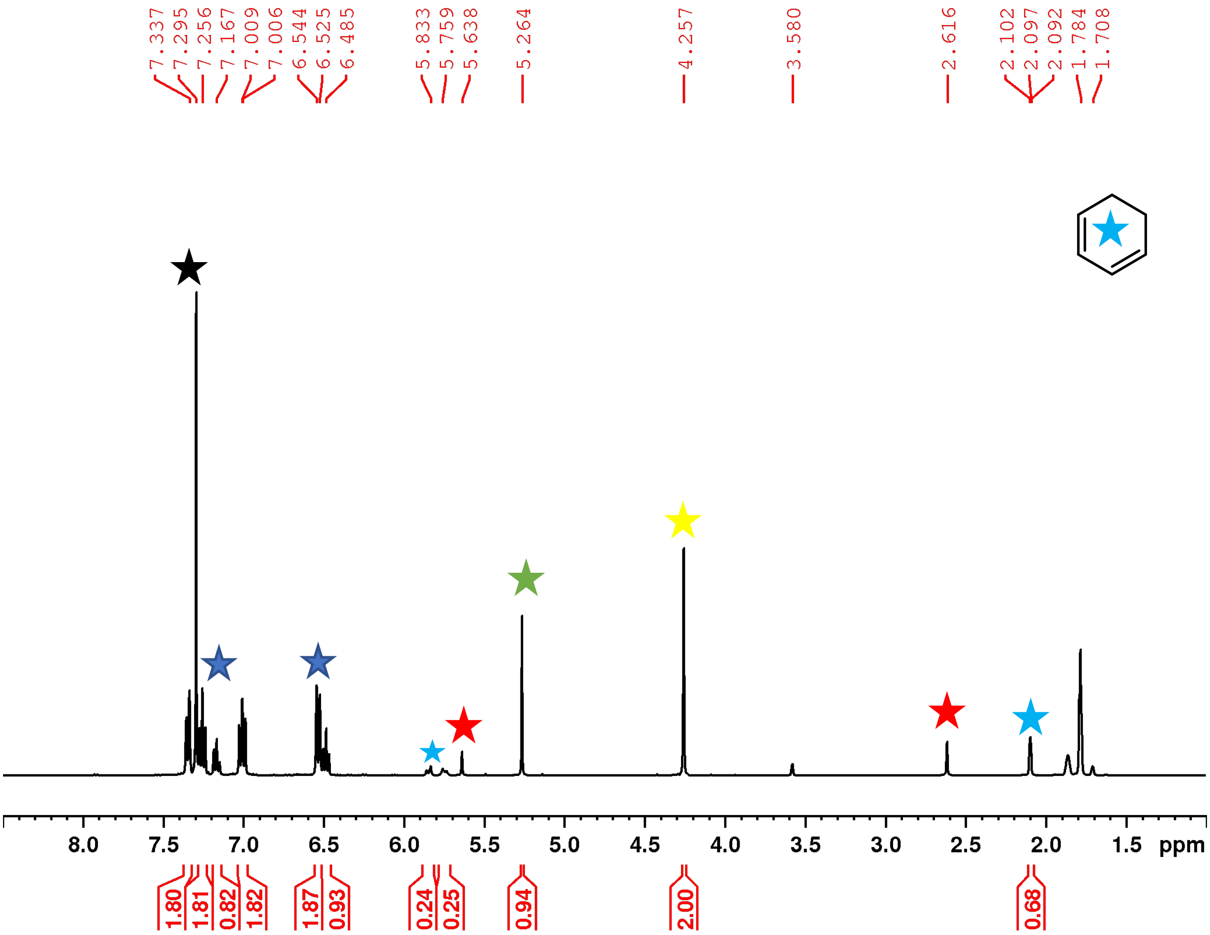

Figure S39 Control reaction of a 10 mol% sample of **2** in THF-d_8 which has been reacted with equimolar quantities of 1,4-CHD and the imine (*N*-benzylideneaniline) at 70 °C for 10 hours. This reaction was successful providing an NMR yield of 98% of the target product.

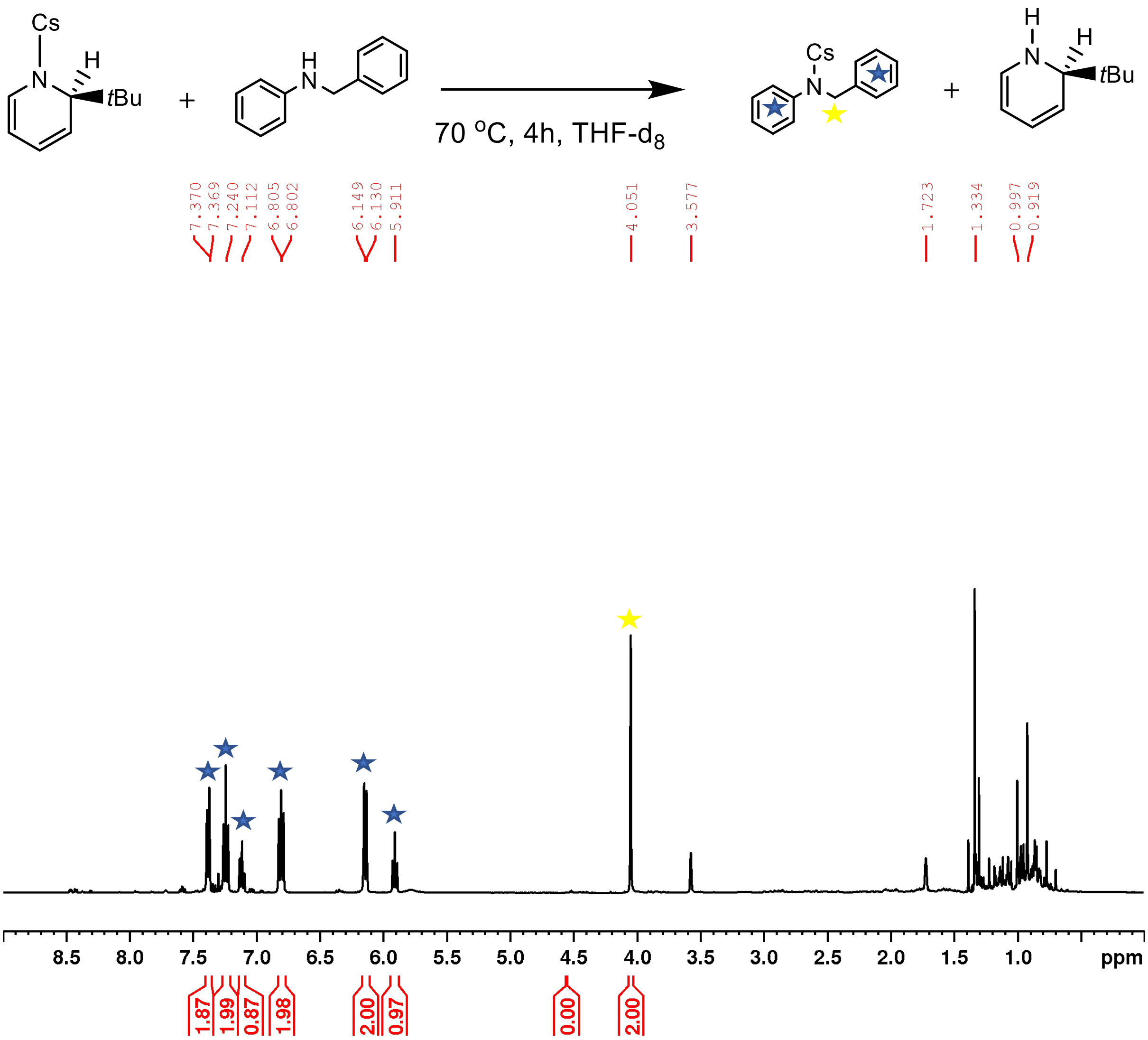

Figure S40 Control stoichiometric reaction between Cs(*t*BuDHP) and *N*-Benzylaniline in THF-d_8 after heating for 4 hours.

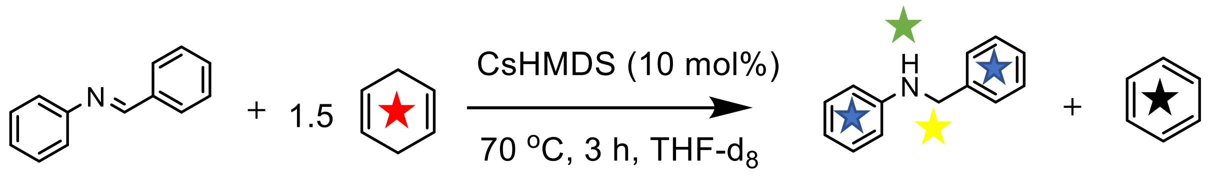

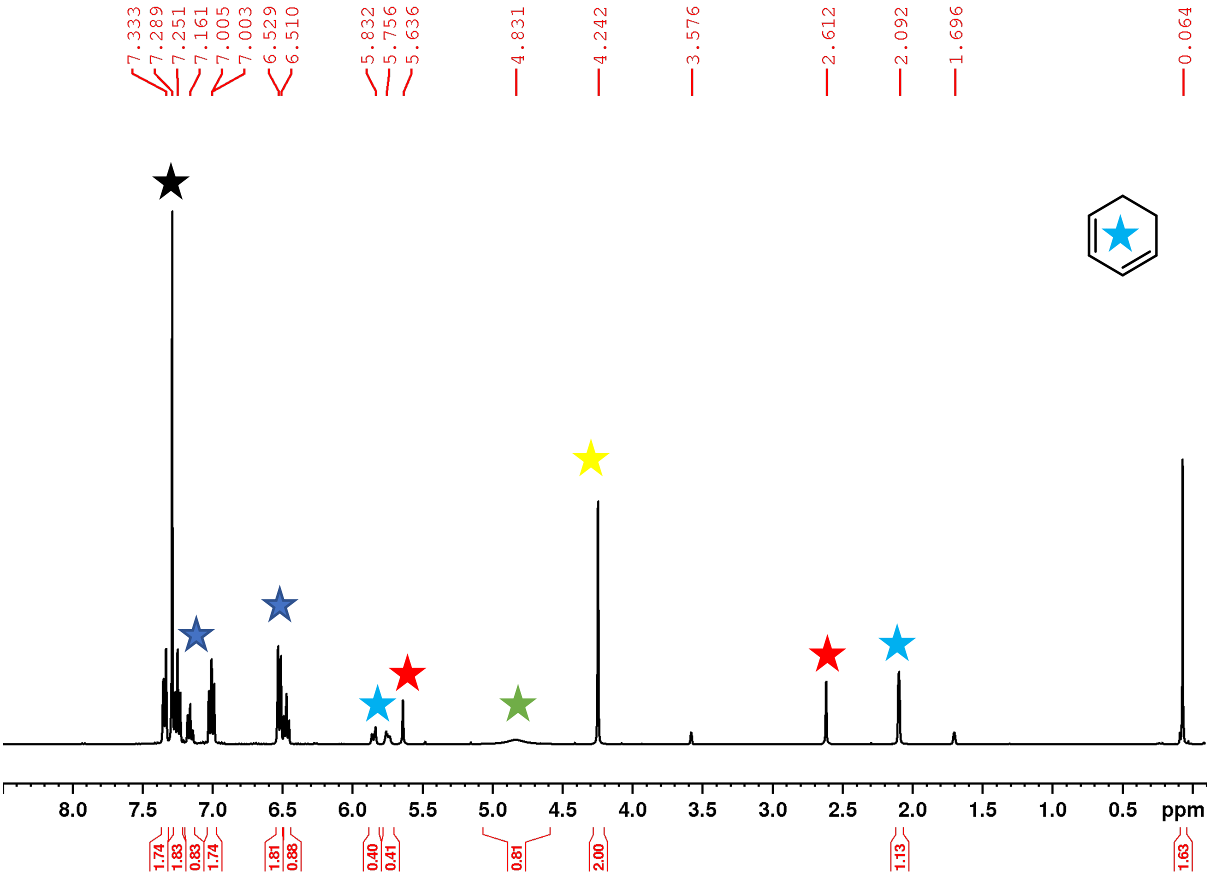

Figure S41 Reaction using Cs[N(SiMe$_3$)$_2$] as catalyst (10 mol%) for the transfer hydrogenation of *N*-benzylideneaniline with 1,4-CHD in THF-d$_8$. This reaction was found to reach 98% conversion after heating the mixture for 3 hours at 70 °C

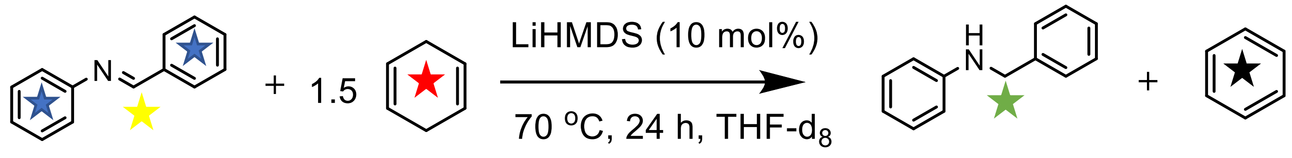

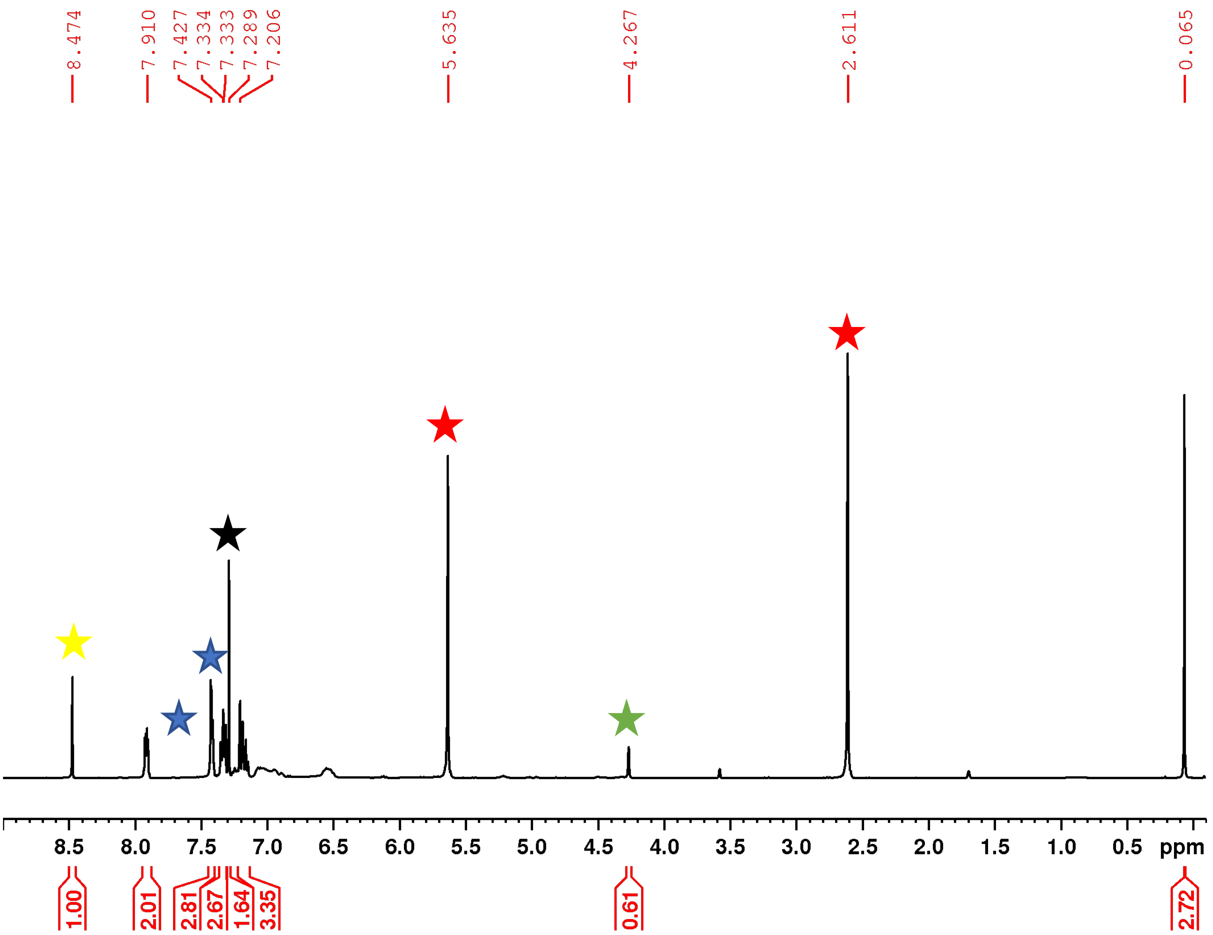

Figure S42 Reaction using $Li[N(SiMe_3)_2]$ as catalyst (10 mol%) for the transfer hydrogenation of *N*-benzylideneaniline with 1,4-CHD in THF-d_8. This reaction was found to only reach 10% conversion after heating the mixture for 24 hours at 70 °C

9 Lithium and Potassium mechanism energy diagrams

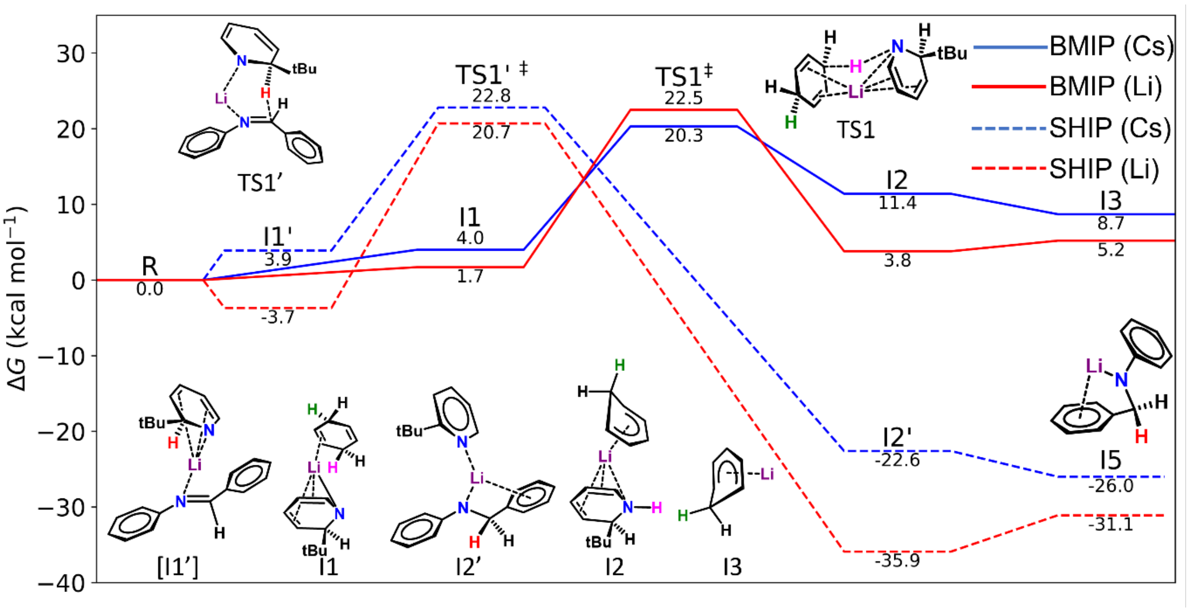

Figure S43 Gibbs free energy profile for the catalytic initiation mechanisms, (Solid) Base Mediated Initiation Pathway (BMIP), (Dashed) Surrogate Hydride Initiation Pathway (SHIP) for Caesium (blue) and lithium (red).

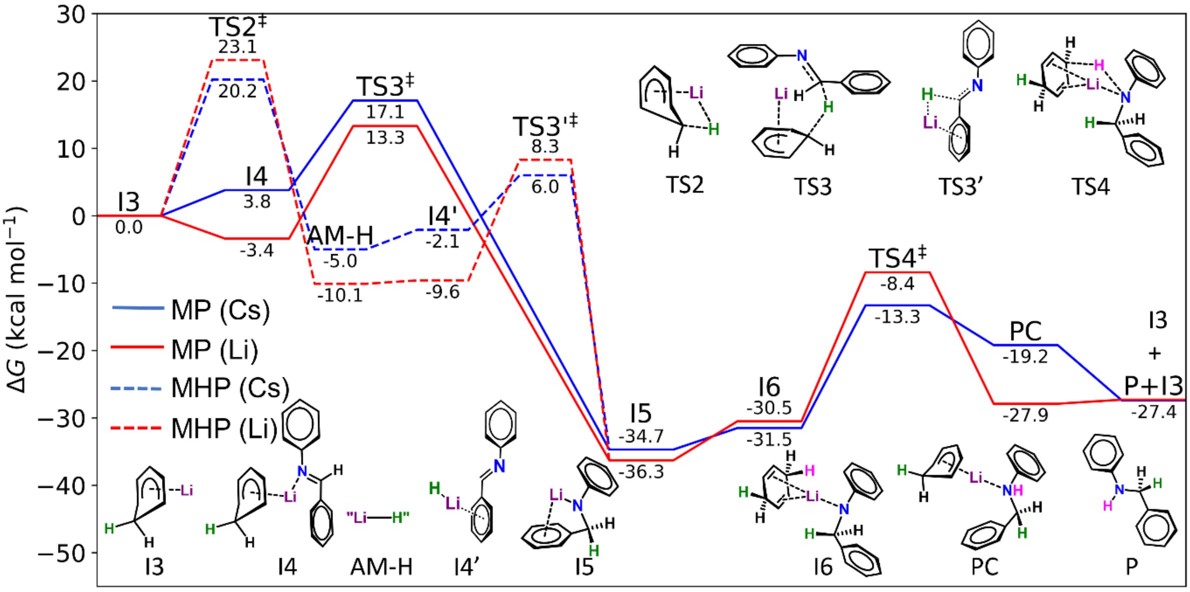

Figure S44 Gibbs free energy profile for the catalytic cycle mechanisms, for caesium (blue) and lithium (red). The solid energy profile represents Meisenheimer Pathway (MP) and dashed energy profile represents the Metal Hydride Pathway (MHP).

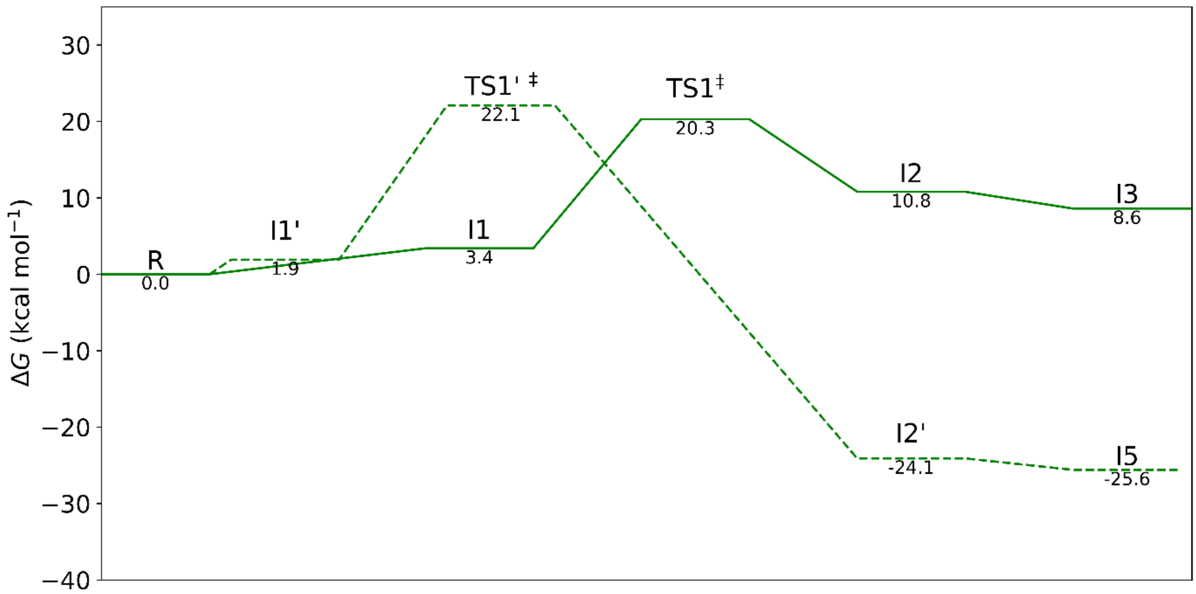

Figure S45 Gibbs free energy profile for the catalytic initiation mechanisms, (Solid) Base Mediated Initiation Pathway (BMIP), (Dashed) Surrogate Hydride Initiation Pathway (SHIP) for potassium.

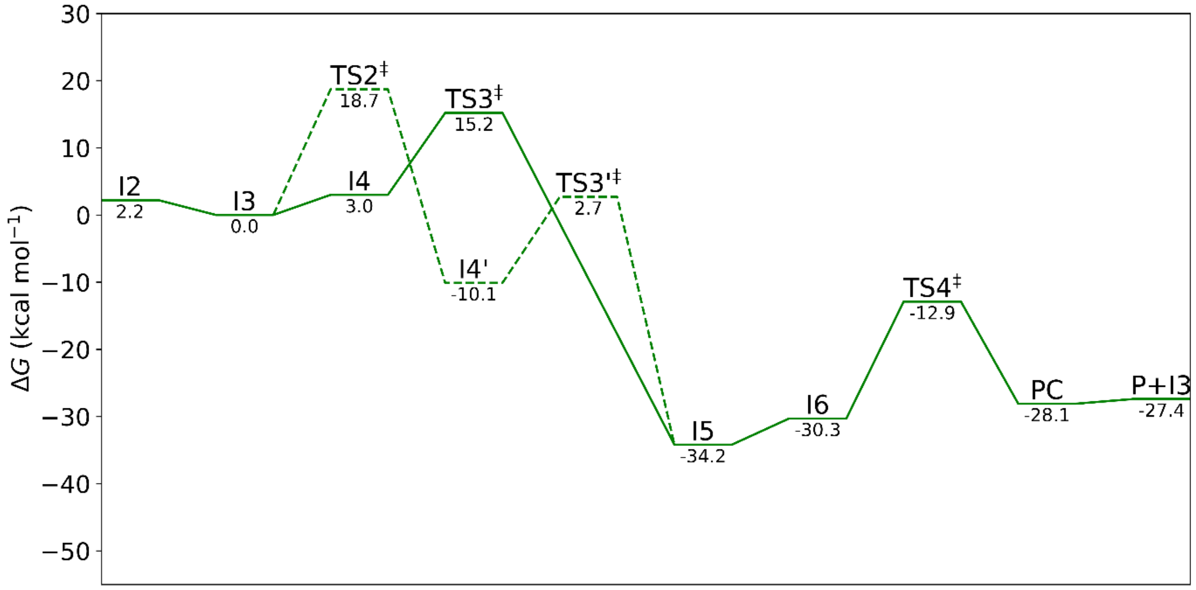

Figure S46 Gibbs free energy profile for the catalytic cycle mechanisms, for potassium. The solid energy profile represents Meisenheimer Pathway (MP) and dashed energy profile represents the Metal Hydride Pathway (MHP).The relative Gibbs free energy values have been re-zeroed at I3.

10 Parallel H_2 pathway

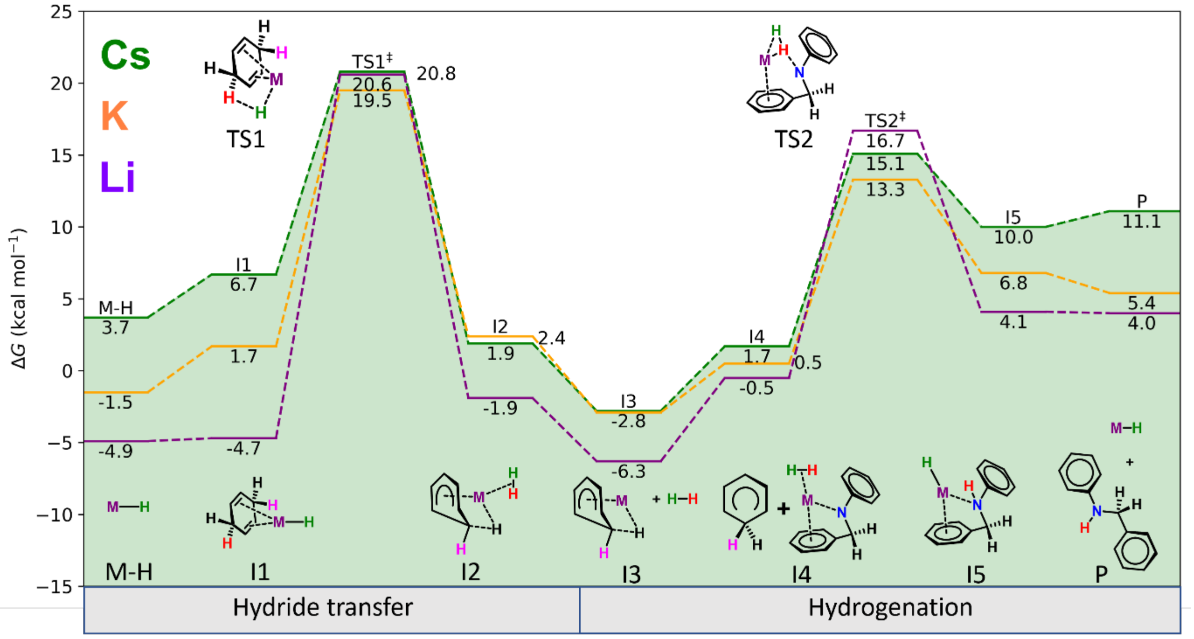

Figure S47 Parallel H_2 pathway

11 Deep neural network optimiser

To achieve DFT level accuracy from the deep neural network (DNN) the potential needs to be trained on >10^6 relevant structures for each new element that is included in the potential. In order to generate these structures, along with their corresponding energies and forces, at the DFT level of theory we will employed including generating conformers of chemically feasible molecules with up to 7 heavy atoms, generating reactions mechanisms *via* AutoDE,[1] extracting structures from high temperature molecular dynamics trajectories and brute force generation of atomic grids.

By employing this approach, the proposed mechanisms for K as the AM were evaluated. The structures along these pathways were located by the neural network potential within 10 minutes and the subsequent evaluation of the energies and thermodynamics were carried out over > 1 month period. For comparison, the structure obtained by the neural network for four TS's and the final DFT optimized structure are compared in figure S50.

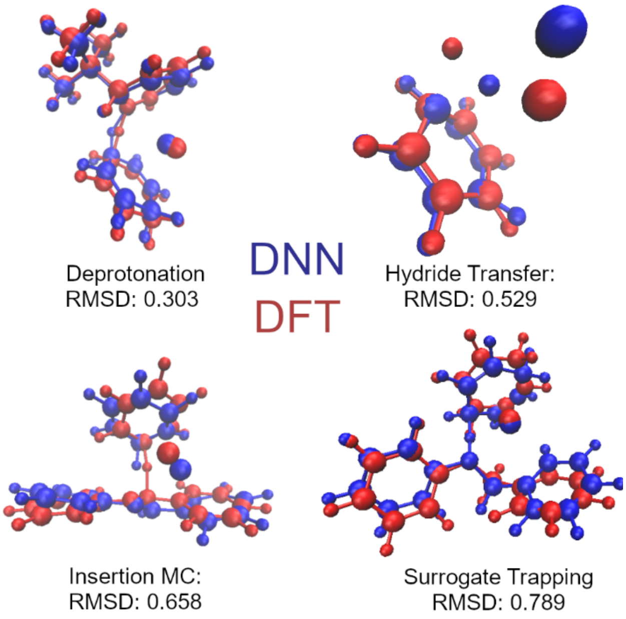

Figure S48 Four organopotassium transition states calculated *via* DFT and the DNN, each with an RMSD of < 1.0.

It must be noted that the energies were not calculated by the DNN but exclusively *via* DFT and the DNN at this stage acts only as an improved method for the initial guess at optimized geometry.

The dataset of organopotassium molecules and their energies was curated in a similar fashion to the original ANI-1 dataset generated by Roitberg *et al*.[2] and appended to it as allowing the deep learning algorithm access to more data in the same format is a common method of extending the data available cheaply. Our dataset was generated by taking every molecule of 6 or less heavy atoms (excluding potassium) in the GDB-11 dataset[3] and, using the RDKit software package, iteratively swapping potassium atoms with each of the hydrogen atoms in the saturated molecule (by default RDKit saturates molecules in such as a way that the charge will be 0 and the multiplicity will be 1). RDKit conformer generation was used because it has been successfully implemented in similar quantum machine learning research.[4]

For each organopotassium compound, K conformers (Equation 1) are generated using the stochastic RDKit embedding method. The number of conformers for each compound is scaled according to S, a constant that varies depending on the number of heavy atoms (excl. potassium) used to scale number of conformers as a consideration of the information each provides (more atoms = more information) and the time to calculate the energy using DFT methods. Values for S are the same as in the original ANI-1 dataset publication.[2]

Equation 1. Number of conformers generated for the dataset depends on the number of atoms (N).

$$K = S(N3 - 1)$$

An energy calculation if performed for each conformer using the wB97X[5] DFT functional with the 6-31G* basis set in the Gaussian 16 software package.[6] The model is trained using a shuffled 80/20 train-test split and an MSE loss until the validation RMSE is no longer improving with patience (100 epochs) at which point the reduce learning rate on plateau scheduler decays the learning rate and the process is repeated until the learning rate decays below a threshold value.

After the training on the initial dataset, query by committee (QBC) is used to select high energy conformers which the models do not predict well (defined as variance between model predictions) as these provide more information per conformer to the DNN. The committee is made up of 3 ANI models each trained with a different batch size as well as different data in the training and test sets. The high energy conformations are generated by running solvent molecular dynamics trajectories at high temperature, random placement of atoms in grids as well as transition states generated by the AutodE package.[1] By retraining on a dataset that includes these high energy conformers we find the DNN is better able to find transition states.

12 General experimental procedures

All synthetic procedures were performed under nitrogen (N_2) atmosphere using standard Schlenk techniques or in a glove box under argon (Ar) atmosphere. Prior to use, glassware was dried at 150 °C under vacuum and solvents were dried, distilled and degassed using standard methods.[7] n-Hexane, and tetrahydrofuran (THF) were dried by heating to reflux over sodium and benzophenone under N_2. Benzene (C_6D_6), and THF-d_8 were dried over molecular sieves (4 Å) and stored in the glove box. n-Pentane, toluene and THF were dried in the Solvent Purification System (Innovative Technology, PS-Micro), degassed, and stored under inert atmosphere over activated 4 Å molecular sieves. Benzene and pyridine were dried over CaH_2, distilled under N_2 atmosphere, and stored over activated 4 Å molecular sieves prior to use. *tert*-Butyllithium (*t*BuLi), rubidium fluoride, caesium fluoride, N-Benzylidene-*tert*-butylamine (PhC(H)=N*t*Bu) and benzophenone imine were obtained from commercial sources.

1-Alkali-metal-2-*tert*-butyl-1,2-dihydropyridine compounds 1-Li-2-*t*Bu(DHP), 1-Na-2-*t*Bu(DHP), 1-K-2-*t*Bu(DHP)[8], 1-Rb-2-*t*Bu(DHP) and 1-Cs-2-*t*Bu(DHP)[9] were synthesized and stored in the glove box at -20 °C as solid material following literature procedures. Li[N(SiMe$_3$)$_2$], Rb[N(SiMe$_3$)$_2$][10], Cs[N(SiMe$_3$)$_2$][11], RbOAm, CsOAm and *N*-benzylideneaniline[12] were synthesized following literature procedure and stored at room temperature in the glove box as white powder. NMR data for each was equivalent to that stated.

^{1}H, ^{13}C, ^{7}Li, ^{27}Al, DEPTQ135, COSY, and HSQC NMR spectra were recorded on an AV 400 MHz spectrometer. All ^{13}C spectra were proton decoupled. Chemical shifts (δ in ppm) in the ^{1}H and ^{13}C NMR spectra were referenced to the residual signals of the deuterated solvents. For describing signal multiplicities common abbreviations have been used: s (singlet), d (doublet), t (triplet), q (quartet), dd (doublet of a doublet), m (multiplet) and br (broad)

Catalytic reactions were completed as follows: Standard solutions were made up of each catalyst using deuterated solvents (C_6D_6, THF-d_8) The catalyst (0.03 mmol for 10 mol%; 0.015 mmol for 5 mol%; 0.0075 mmol for 2.5 mol%; 0.00375 mmol for 1.25 mol%) was added into a J Youngs tube along with 0.45 mL of the deuterated solvent (C_6D_6 or THF-D_8). The imine (0.3 mmol) was then added to the Youngs tube along with a suitable standard (Adamantane) and 1.1-1.5 eqv. of 1,4-cyclohexadiene (0.6 mmol). The reaction could then be heated to its desired temperature and monitored via NMR until completion. ^{1}H NMR spectraums were recorded every 30 minutes capped at a total time of 24 hours.

Stoichiometric reactions between the organometallic intermediates and 1,4-cyclohexadiene were taken as follows; The isolated intermediates (0.15 mmol) were dissolved in a dry J Youngs tube with deuterated solvent (C_6D_6 or THF-D_8). Subsequently 1 equiv. of 1,4-cyclohexadiene (0.15 mmol) was added to the solution. The reaction was then held at a suitable temperature (70 °C) where conversion was determined by ^{1}H NMR spectroscopy

every 30 minutes. The same procedure followed for stoichiometric reactions between Cs(*t*BuDHP) and 1,4-CHD.

Crystals were layered with perfluoropolyalkylether oil before mounting on the X-Ray diffractometer. The oil coated crystals started bubbling on the glass slide as soon as they met the atmosphere. Care was taken to quickly mount a single crystal on the goniometer. Data for all compounds were measured with Rigaku Synergy-i or Oxford Diffraction instruments using monochromated λ = 1.54184 or 0.71073 Å radiation, respectively. In all cases, data collection and processing used CrysalisPro software.[13] The structures were refined to convergence against F^2 using all independent reflections and SHELXL.[14] For compound **3** both the pyridine ligand and the partially present free pyridine solvate were treated as disordered. In all cases two site models were used. Suitable restraints and constraints were applied to the bond lengths and displacement parameters of the disordered groups in order to ensure that they approximated normal behaviour. Selected crystallographic data and refinement parameters are presented in Table S3. Deposition numbers 2183221-2183224 and 2214971 contain the full supplementary crystallographic data for this paper in cif format. These data are provided free of charge by the joint Cambridge Crystallographic Data Centre and Fachinformationszentrum Karlsruhe Access Structures service www.ccdc.cam.ac.uk/structures.

13 Synthetic procedures

13.1 Synthesis of of $[PhCH_2N(Li)Ph]_\infty$, (**1**)

$Li[N(SiMe_3)_2]$, (0.334 g, 2 mmol) was added to a Schlenk flask along with benzene (10 ml). To this *N*-benzylaniline $[PhCH_2N(H)Ph]$ (0.366 g, 2 mmol) was added via a solid addition tube and left to stir for 2 hours. The solvent was then removed under reduced pressure where the white solid was dried and transferred into the glovebox and weighed (0.205 g, 54%). The solid was then redissolved in hexane where a crop of colourless crystals were grown (0.056 g, 27%). The crystals were then characterised via NMR and XRD analysis (Section 5).

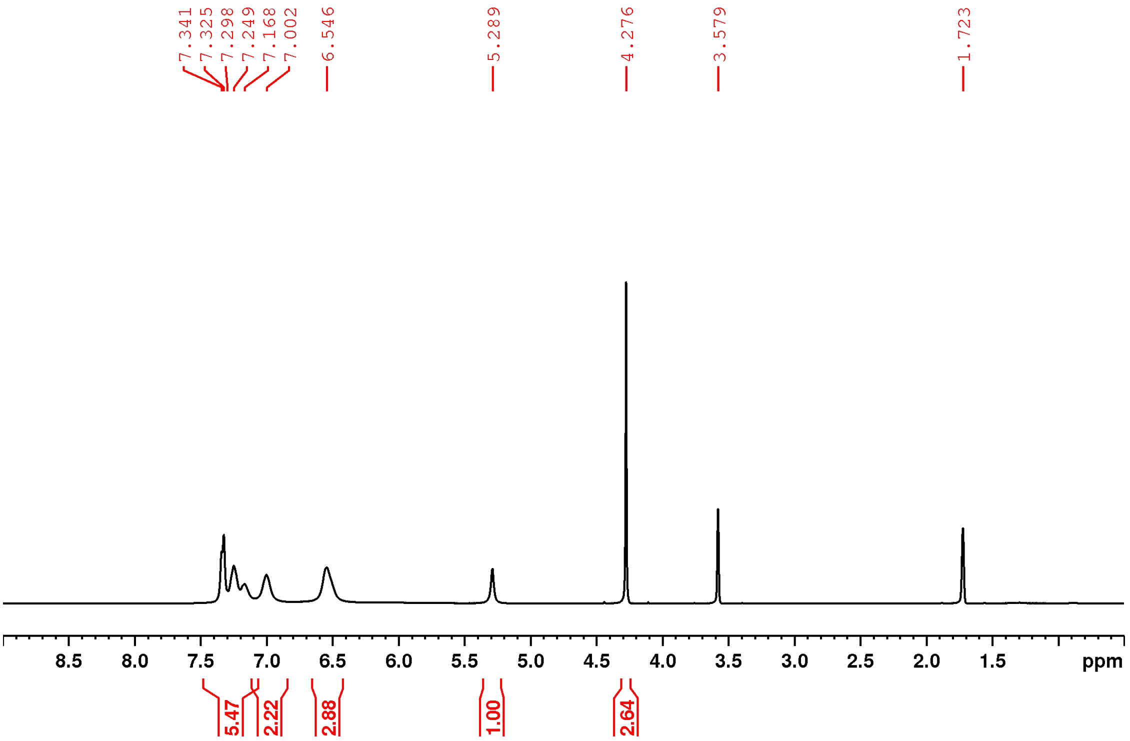

Figure S49 ^{1}H NMR spectrum of $[PhCH_2N(Li)Ph]_\infty$ (**1**) in THF-d_8

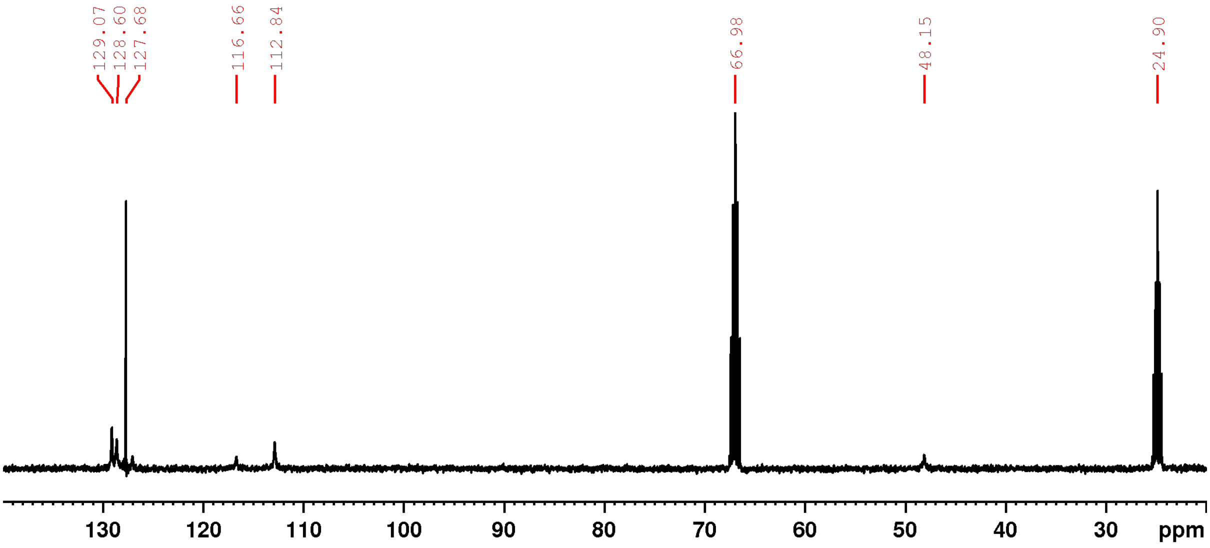

Figure S50 ^{13}C NMR spectrum of $[PhCH_2N(Li)Ph]_\infty$ (**1**) in THF-d_8

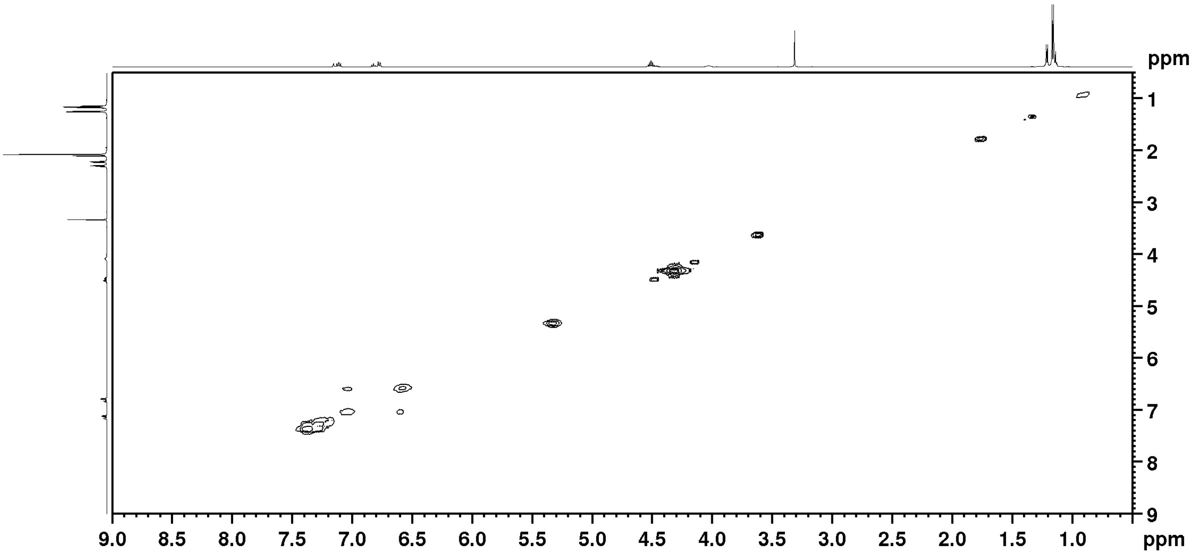

Figure S51 $^1H^1H$-COSY NMR spectrum of $[PhCH_2N(Li)Ph]_\infty$ (**1**) in THF-d_8

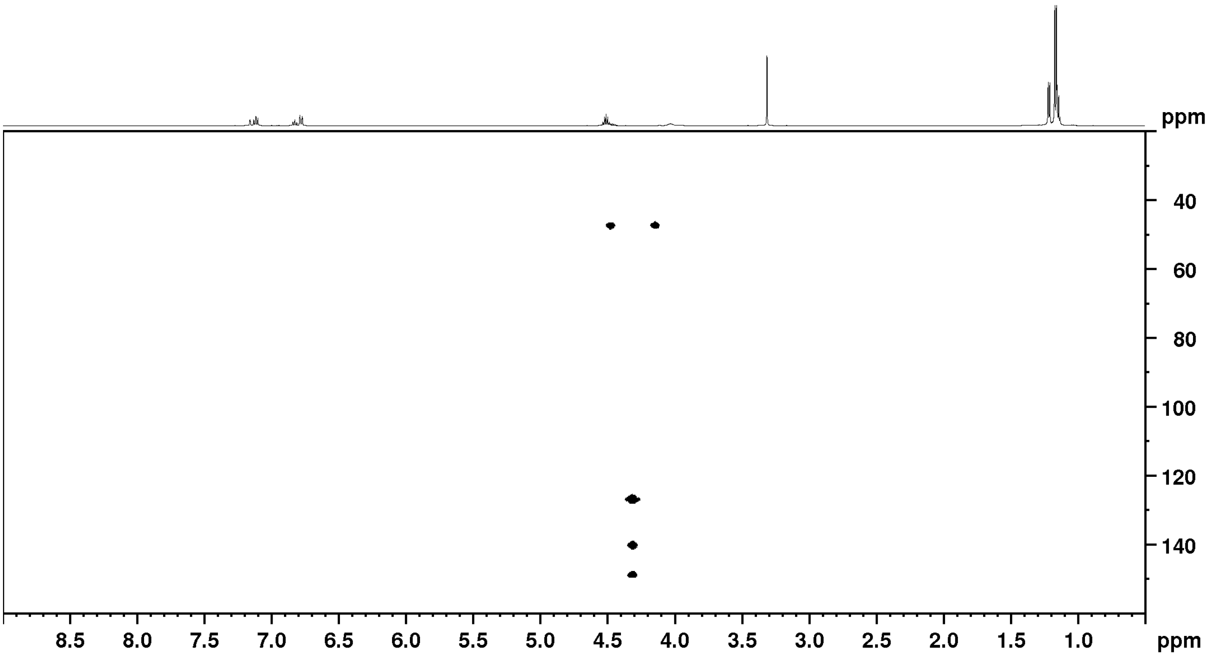

Figure S52 $^{1}H^{13}C$-HSQC NMR spectrum of $[PhCH_2N(Li)Ph]_\infty$ (**1**) in THF-d_8

13.2 Synthesis of [$PhCH_2N(Cs)Ph$]$_\infty$, (**2**)

Cs[$N(SiMe_3)_2$], (0.626 g, 2 mmol) was added to a Schlenk flask along with benzene (10 ml). To this *N*-benzylaniline [$PhCH_2N(H)Ph$] (0.366 g, 2 mmol) was added via a solid addition tube and left to stir for 4 hours. The solvent was then removed under reduced pressure where the pale yellow solid was dried and transferred into the glovebox and weighed (0.416 g, 65%). A small crop of yellow/brown crystals were then grown from a solution of hexane (0.056 g, 27%). The crystals were then characterised via NMR spectroscopy and XRD analysis.

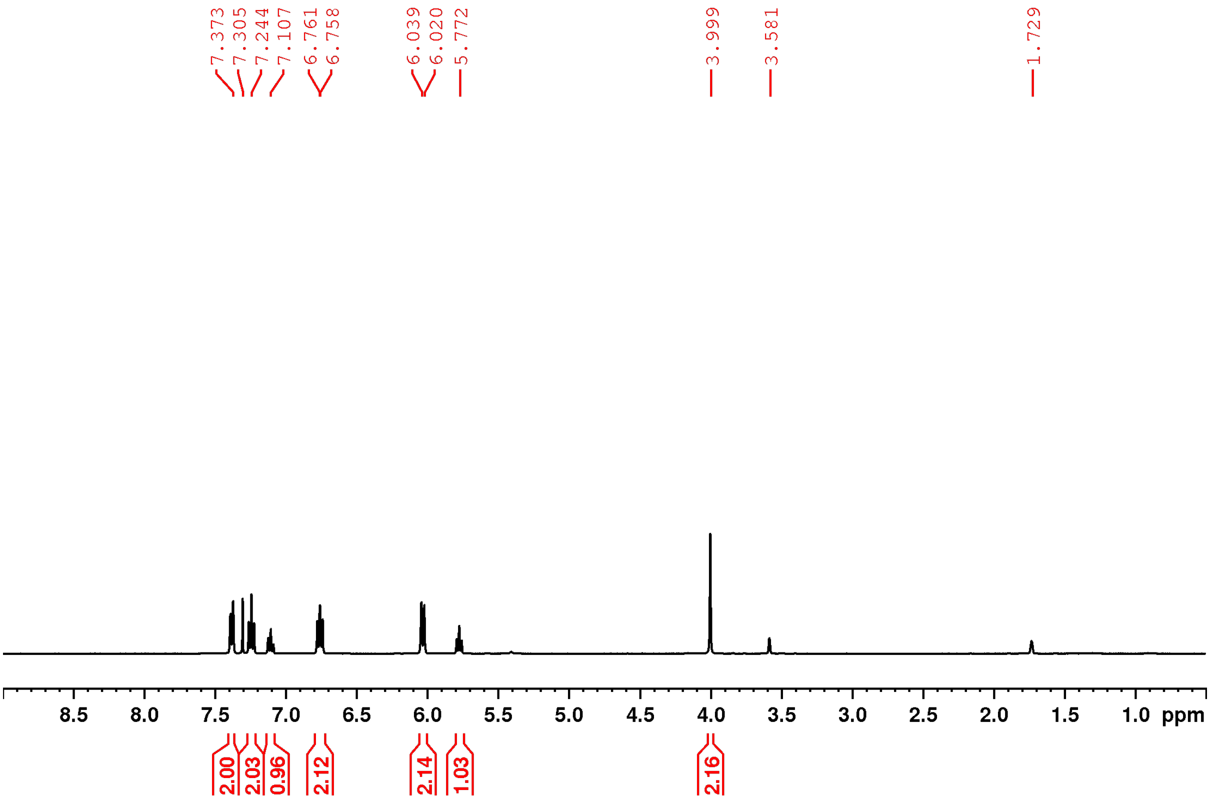

Figure S53 ^{1}H NMR spectrum of [$PhCH_2N(Cs)Ph$]$_\infty$, (**2**) in THF-d_8

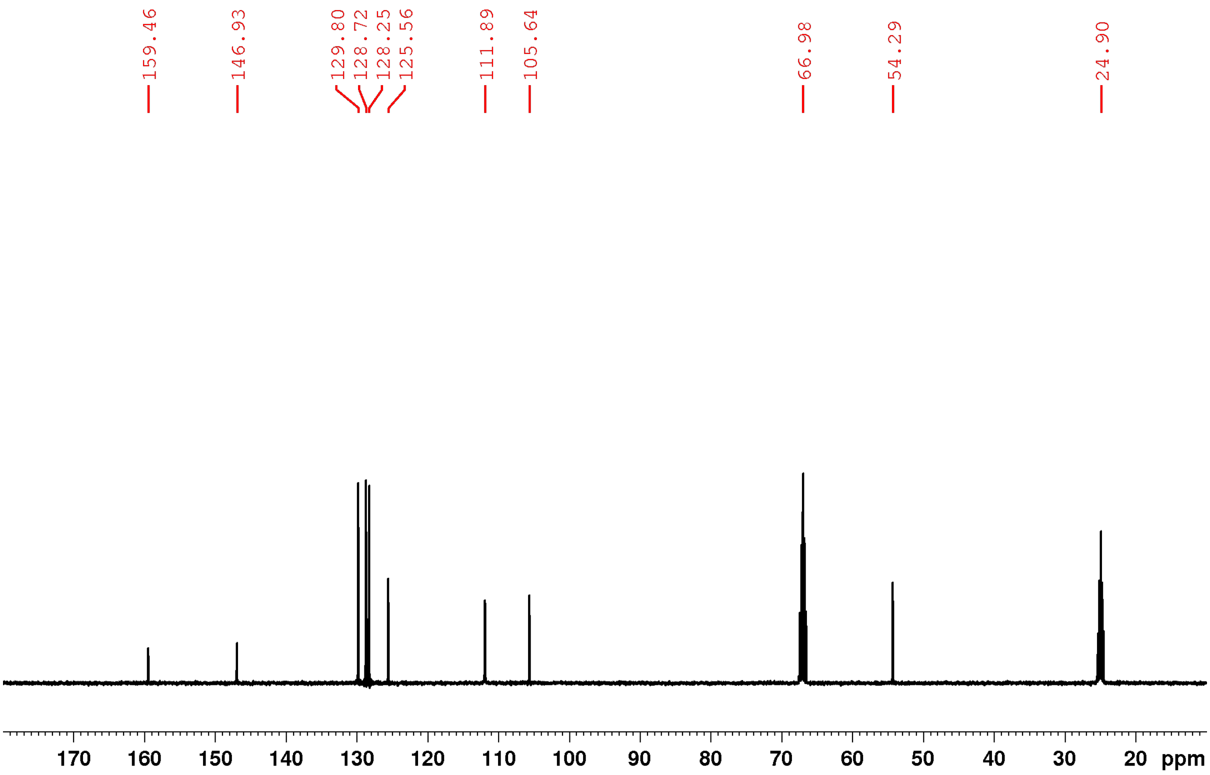

Figure S54 ^{13}C NMR spectrum of [PhCH$_2$N(Cs)Ph]$_\infty$, (**2**) in THF-d$_8$

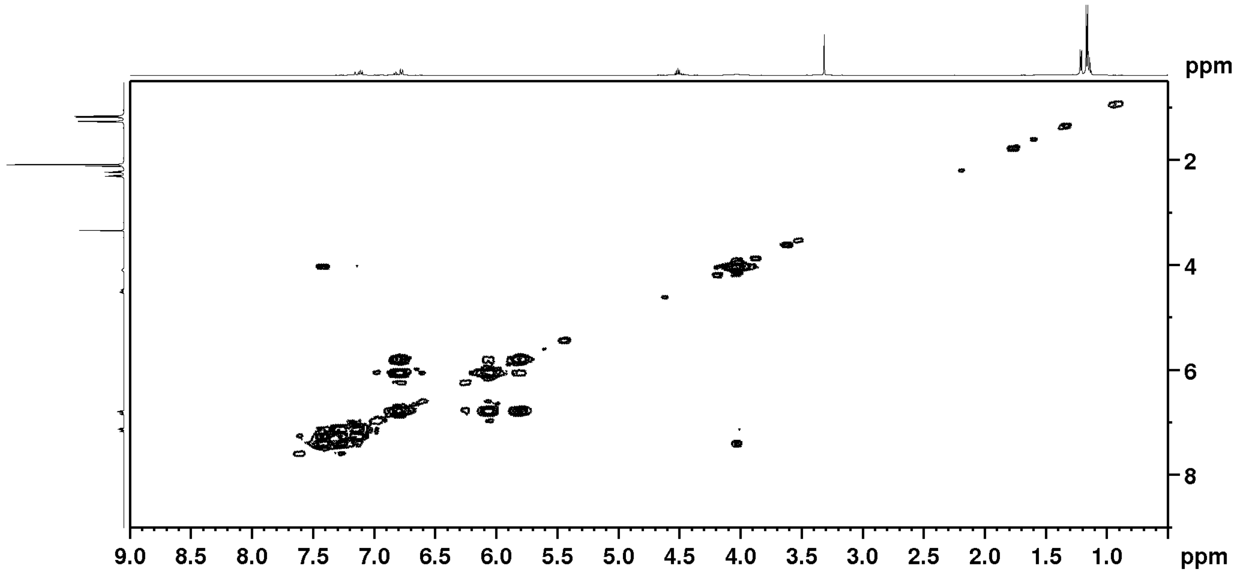

Figure S55 ^{1}H^{1}H-COSY NMR spectrum of [PhCH$_2$N(Cs)Ph]$_\infty$, (**2**) in THF-d$_8$

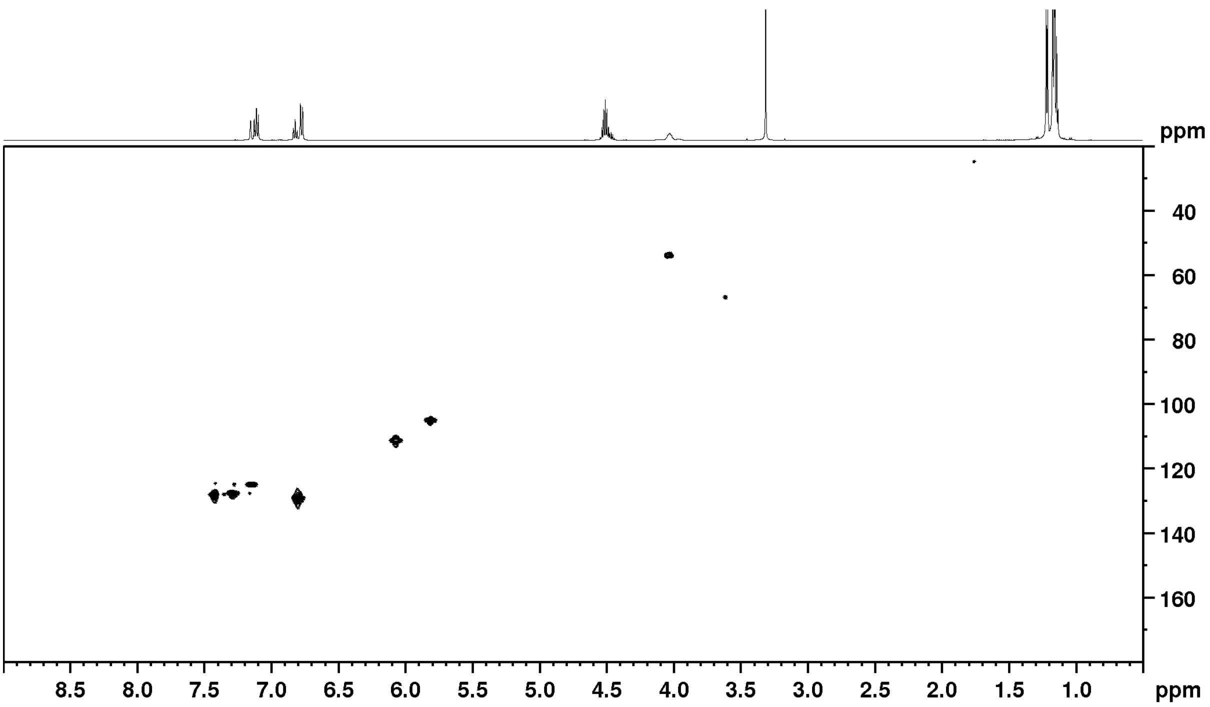

Figure S56 $^{1}H^{13}C$-HSQC NMR spectrum of $[PhCH_2N(Cs)Ph]_{\infty}$, (**2**) in THF-d_8

13.3 Synthesis of pyridine solvate {[Cs(*t*BuDHP)]$_2$·py}$_\infty$ (**3**)

1,2-Cs-*t*Bu(DHP), (0.538 g, 2 mmol) was added to a Schlenk flask along with excess pyridine (4 ml). The dark red solution was taken into the glovebox where it was transferred into vials for crystallisation. After layering with hexane the vial was placed into the -20 °C freezer where a small crop of crystals grew. These were then analysed via NMR spectroscopy and XRD measurements.

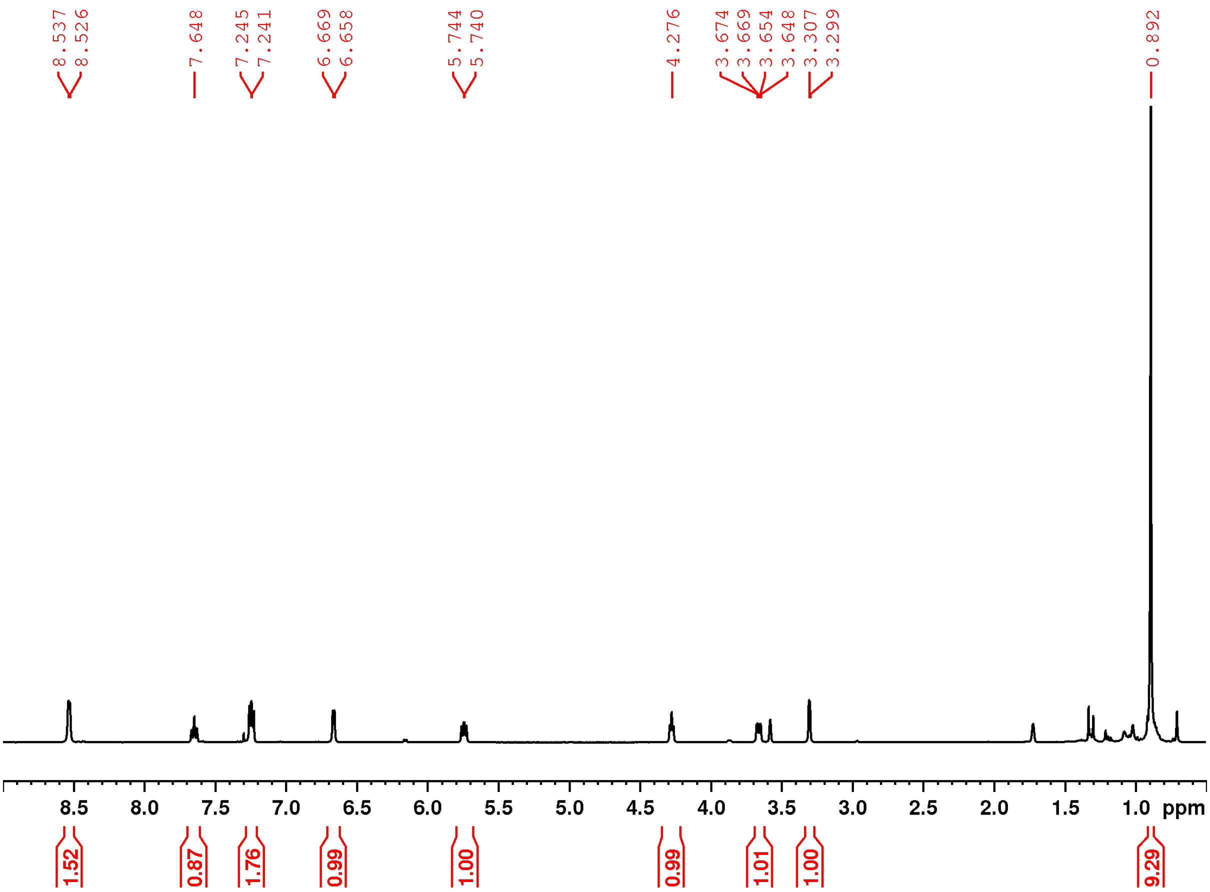

Figure S57 ^{1}H NMR spectrum of {[Cs(*t*BuDHP)]$_2$·py}$_\infty$ (**3**) in THF-d$_8$

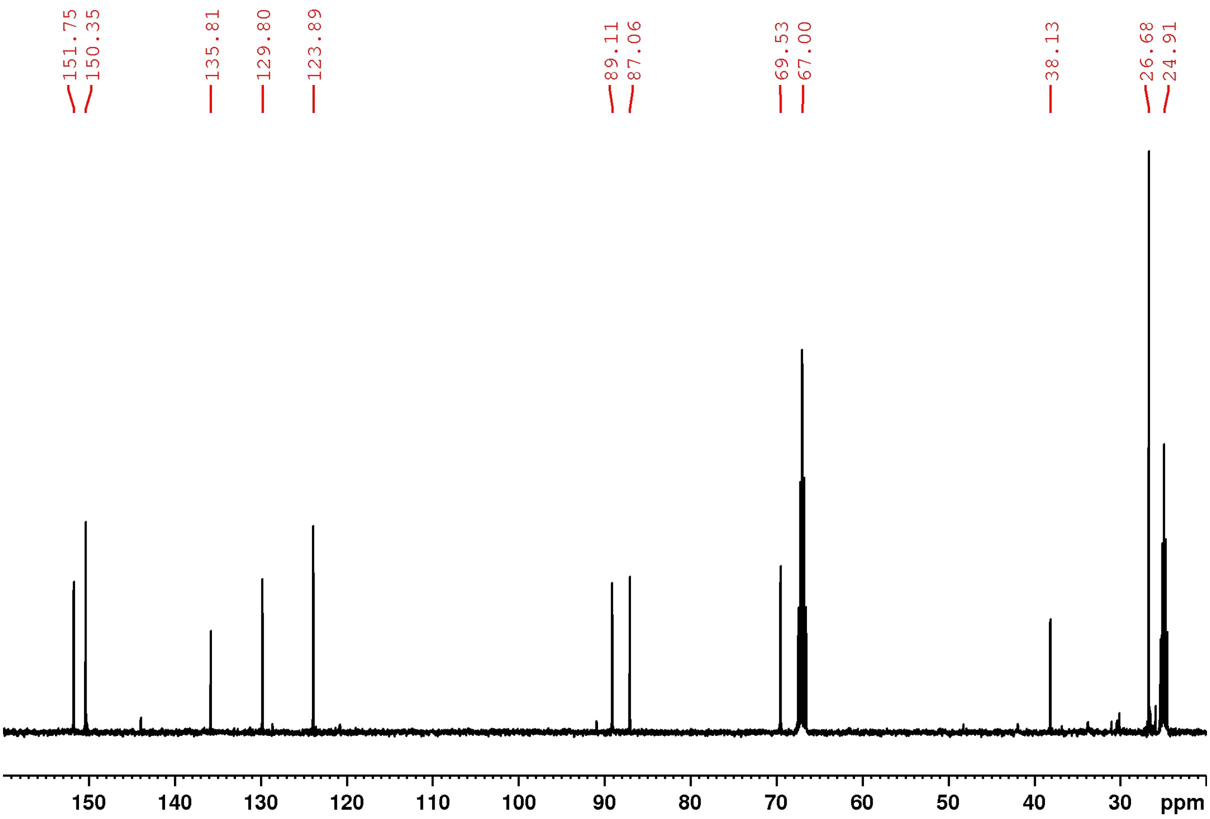

Figure S58 ^{13}C NMR spectrum of {[Cs(*t*BuDHP)]$_2$·py}$_\infty$ (**3**) in THF-d$_8$

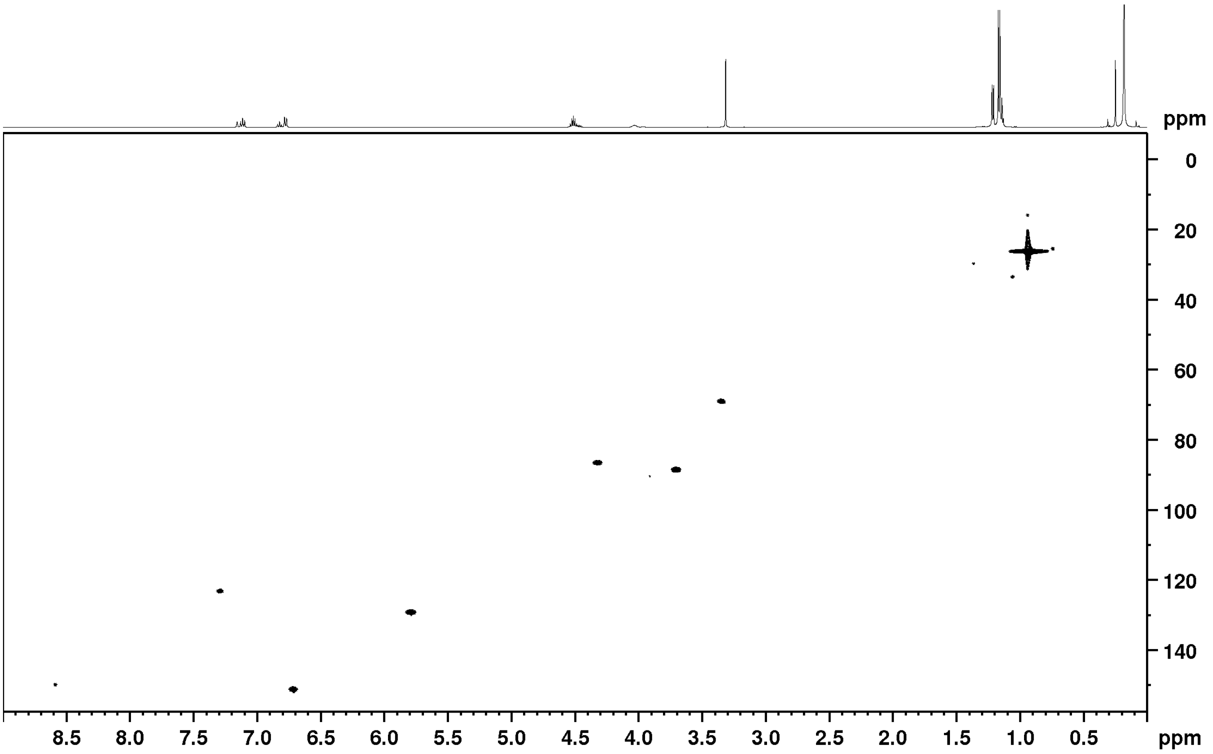

Figure S59 $^{1}H^{13}C$-HSQC NMR spectrum of {[Cs(*t*BuDHP)]$_2$·py}$_\infty$ (**3**) in THF-d$_8$

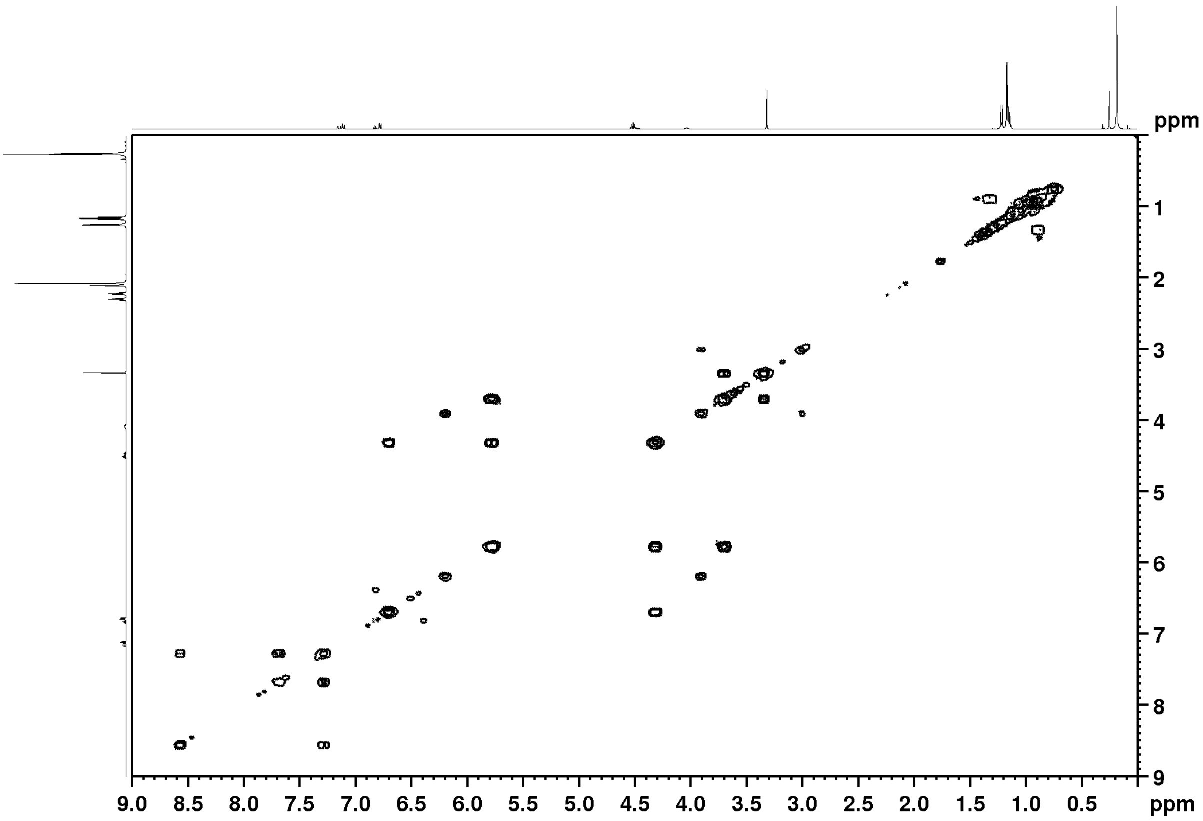

Figure S60 $^{1}H^{1}H$-COSY NMR spectrum of {[Cs(*t*BuDHP)]$_2$·py}$_\infty$ (**3**) in THF-d$_8$

13.4 Synthesis of monomeric tris-pyridine solvate [Li(*t*BuDHP)·(py)$_3$] (**4**)

1,2-Li-*t*Bu(DHP), (0.288 g, 2 mmol) was added to a miniature Schlenk flask along with pyridine (4 ml). On addition of pyridine the solution turned to a dark red colour where it was then layered with pentane and placed in the -20 °C freezer. After 24 hours a crop of dark red crystals had grown along the side of the Schlenk tube. The crystals were isolated and characterized via NMR spectroscopy and XRD measurements.

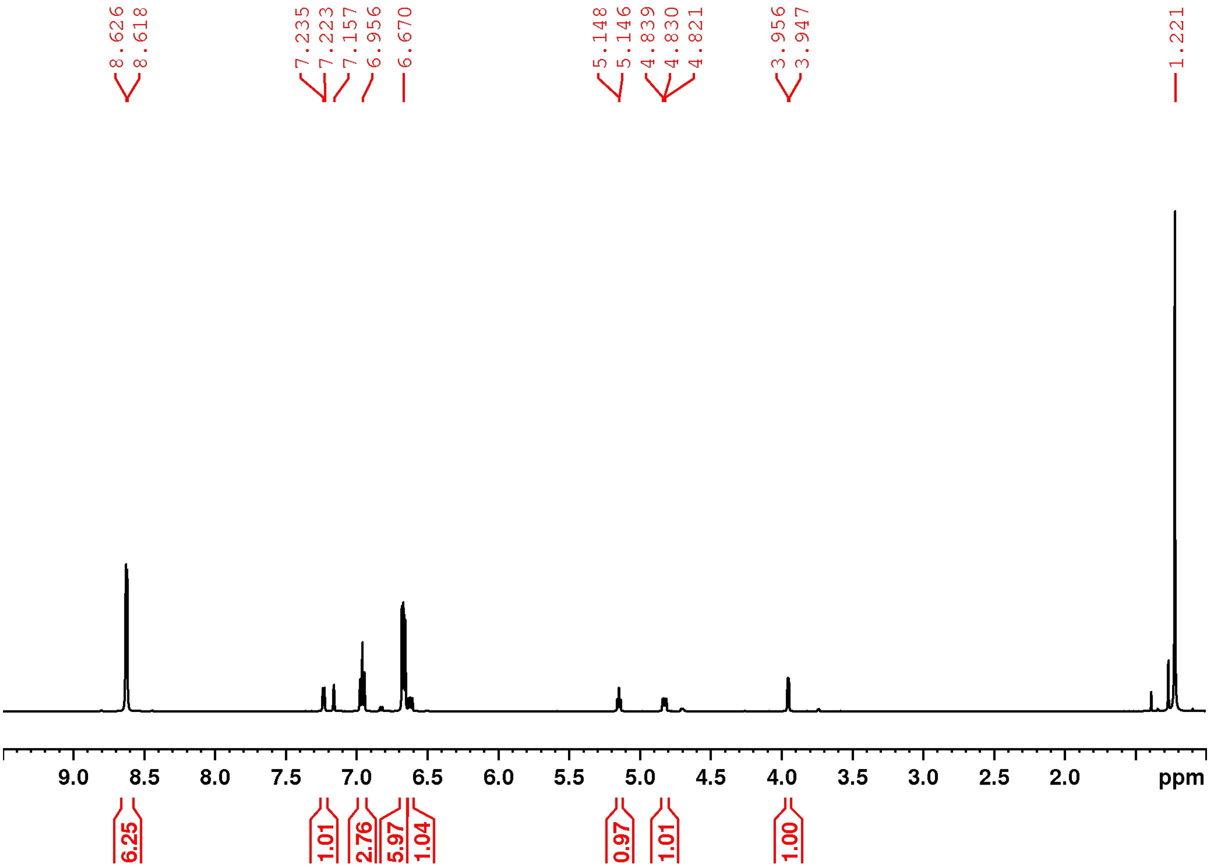

Figure S61 ^{1}H NMR spectrum of [Li(*t*BuDHP)·(py)$_3$] (**4**) in C_6D_6

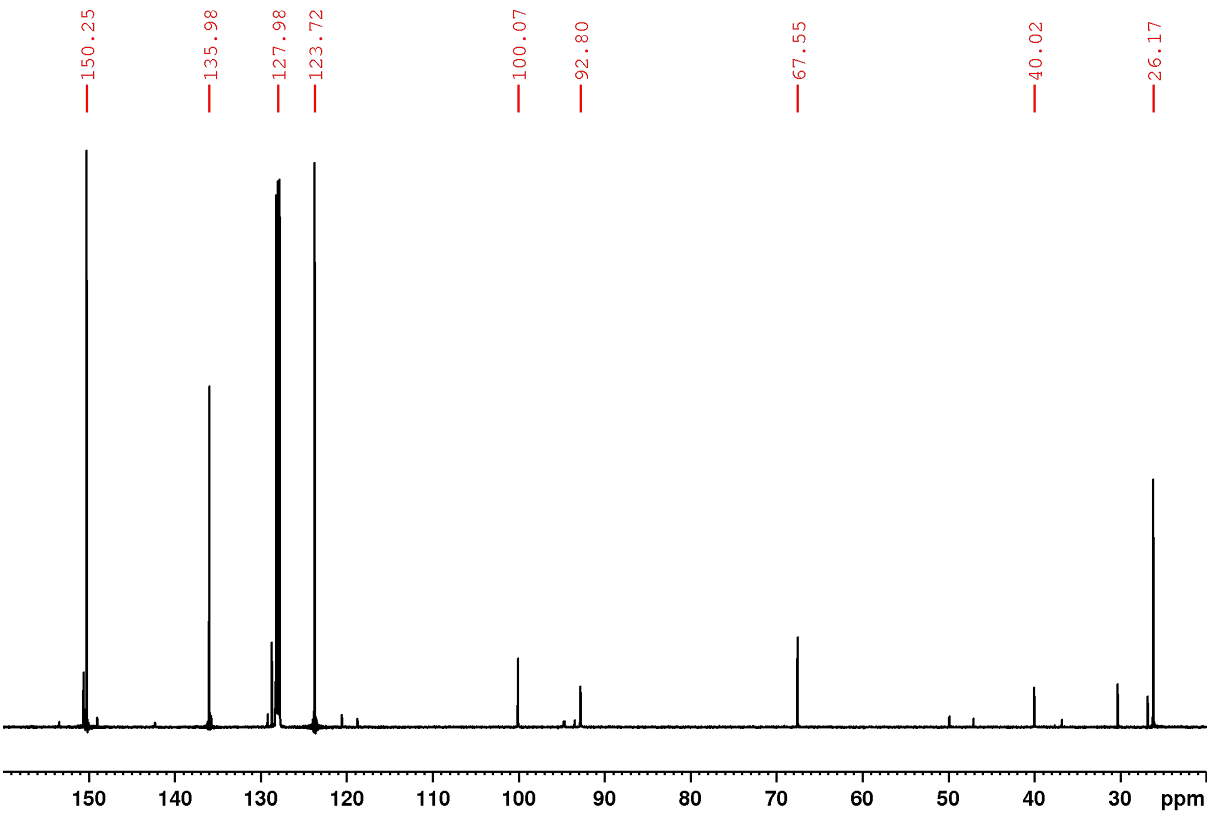

Figure S62 ^{13}C NMR spectrum of [Li(*t*BuDHP)·(py)$_3$] (**4**) in C_6D_6

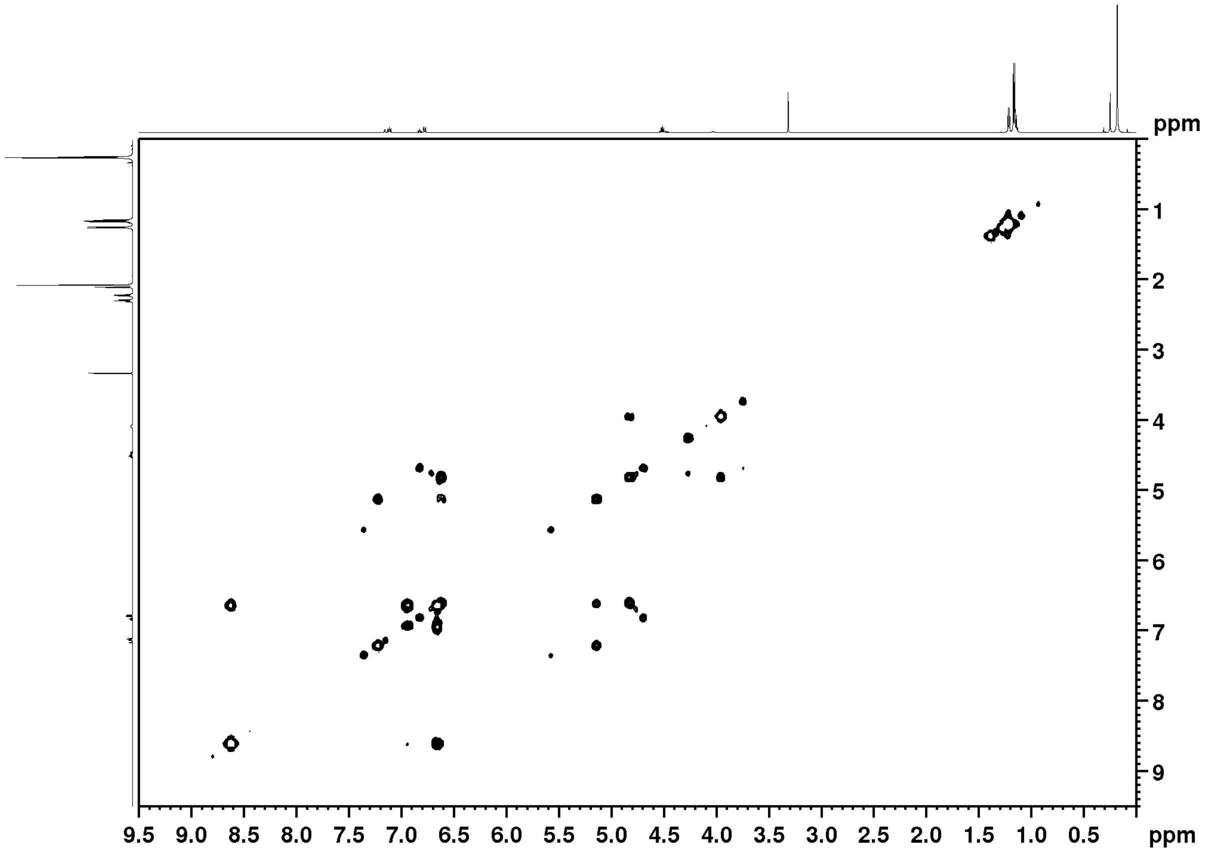

Figure S63 ^{1}H^{1}H-COSY NMR spectrum of [Li(*t*BuDHP)·(py)$_3$] (**4**) in C_6D_6

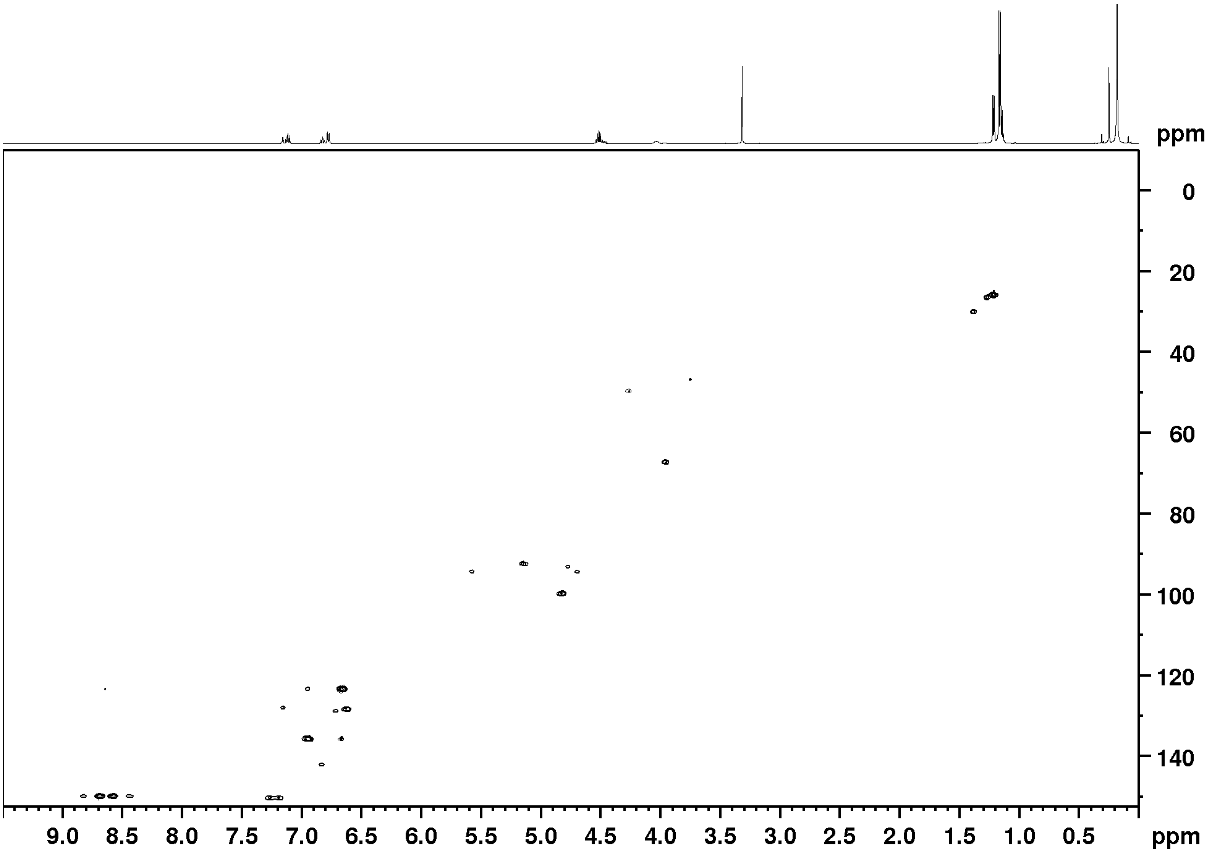

Figure S64 $^{1}H^{13}C$-HSQC NMR spectrum of [Li(*t*BuDHP)·(py)$_3$] (**4**) in C_6D_6

Table S3 Selected crystal structure data and refinement details

Compound	1	2	3	4
CCDC	2183221	2183224	2214971	2183222
Empirical Formula	$C_{13}H_{12}LiN$	$C_{13}H_{12}CsN$	$C_{24.25}H_{34.25}Cs_2N_{3.25}$	$C_{24}H_{29}LiN_4$
Molecular Mass	189.18	315.15	637.12	380.45
X-ray wavelength (Å)	1.54184	0.71073	1.54184	1.54184
Space Group	Monoclinic	Monoclinic	Monoclinic	Triclinic
Crystal system	$P2_1/c$	C2/c	$P2_1/c$	P-1
Temperature (K)	100(2)	153(2)	100(2)	100(2)
a/Å	5.6553(1)	19.0206(3)	13.9043(1)	9.3266(2)
b/Å	12.0113(2)	6.6473(1)	10.0549(1)	9.7255(2)
c/Å	14.7587(2)	20.1483(3)	20.7075(2)	13.0828(3)
α/°	90	90	90	76.604(2)
β/°	94.540(2)	109.236(2)	105.309(1)	84.0415(19)
γ/°	90	90	90	86.188(2)
Volume/Å^3	999.38(3)	2405.24(7)	2792.31(5)	1147.07(5)
Z	4	8	4	2
2θmax °	146.20	58.74	146.57	146.54
Measured Reflections	20665	25909	73193	24862
Unique Reflections	1981	3067	5607	4573
R_{int}	0.0521	0.0340	0.0639	0.0303
Observed Reflections [I>2σI]	1890	2770	5356	4153
No. parameters	184	136	347	285
Goodness of Fit	1.087	1.055	1.107	1.047
R [on F, obs refs only]	0.0429	0.0199	0.0392	0.0476
ωR [on F^2, all data]	0.1116	0.0431	0.1109	0.1300
Largest diff. peak /hole/Å^{-3}	0.285/-0.172	0.574/-0.431	2.069/-1.139	0.366/-0.220

14 References

[1] T. A. Young, J. J. Silcock, A. J. Sterling, F. Duarte, *Angew. Chem. Int. Ed.* **2021**, *60*, 4266-4274.

[2] J. S. Smith, O. Isayev, A. E. Roitberg, *Chem. Sci.* **2017**, *8*, 3192-3203.

[3] (a) T. Fink, H. Bruggesser, J.-L. Reymond, *Angew. Chem. Int. Ed.* **2005**, *44*, 1504-1508; (b) T. Fink, J.-L. Reymond, *J. Chem. Inf. Model* **2007**, *47*, 342-353.

[4] A. S. Christensen, F. A. Faber, O. A. v. Lilienfeld, *J. chem. Phys.* **2019**, *150*, 064105.

[5] J.-D. Chai, M. Head-Gordon, *J. Chem. Phys.* **2008**, *128*, 084106.

[6] M. J. Frisch, G. W. Trucks, H. B. Schlegel, G. E. Scuseria, M. A. Robb, J. R. Cheeseman, G. B. Scalmani, G. A. V.; Petersson, H. Nakatsuji, X. C. Li, A. V. M.; Marenich, J. Bloino, Janesko, , G. B.G., M. R., H. B., O. H.P., J.V., , A. F. Izmaylov, J. L. Sonnenberg, D. Williams-Young, F. Ding, F. Lipparini, F. Egidi, J. Goings, B. Peng, A. Petrone, T. Henderson, D. Ranasinghe, V. G. Zakrzewski, J. Gao, N. Rega, G. Zheng, W. Liang, M. Hada, M. Ehara, K. Toyota, R. Fukuda, J. Hasegawa, M. Ishida, T. Nakajima, Y. Honda, O. Kitao, H. Nakai, T. Vreven, K. Throssell, J. A. Montgomery Jr., J. E. Peralta, F. Ogliaro, M. J. Bearpark, J. J. Heyd, E. N. Brothers, K. N. Kudin, V. N. Staroverov, T. A. Keith, R. Kobayashi, J. Normand, K. Raghavachari, A. P. Rendell, J. C. Burant, S. S. Iyengar, J. Tomasi, M. Cossi, J. M. Millam, M. Klene, C. Adamo, R. Cammi, J. W. Ochterski, R. L. Martin, K. Morokuma, O. Farkas, J. B. Foresman, D. J. Fox*, Vol. GaussView 5.0.*, Gaussian, Inc., Wallingford CT, **2016**.

[7] W. L. F. Armarego, C. Chai, in *Purification of Laboratory Chemicals (Seventh Edition)* (Eds.: W. L. F. Armarego, C. Chai), Butterworth-Heinemann, Boston, **2013**, pp. 1-70.

[8] (a) S. A. Orr, A. R. Kennedy, J. J. Liggat, R. McLellan, R. E. Mulvey, S. D. Robertson, *Dalton Trans.* **2016**, *45*, 6234-6240; (b) S. D. Robertson, A. R. Kennedy, J. J. Liggat, R. E. Mulvey, *Chem. Commun.* **2015**, *51*, 5452-5455.

[9] P. A. Macdonald, S. Banerjee, A. R. Kennedy, R. E. Mulvey, S. D. Robertson, *Polyhedron* **2023**, *234*, 116302.

[10] S. Krieck, P. Schüler, H. Görls, M. Westerhausen, *Dalton Trans.* **2018**, *47*, 12562-12569.

[11] A. I. Ojeda-Amador, A. J. Martínez-Martínez, A. R. Kennedy, C. T. O'Hara, *Inorg. Chem.* **2016**, *55*, 5719-5728.

[12] S. J. Bae, Y. M. Ha, Y. J. Park, J. Y. Park, Y. M. Song, T. K. Ha, P. Chun, H. R. Moon, H. Y. Chung, *Eur. J. Med. Chem* **2012**, *57*, 383-390.

[13] CrysAlisPro Software system, version 1.171.39.46, Rigaku Corporation, Oxford UK, **2018**

[14] G. M. Seldrick, *Acta Crystallogr. Sect. C Struct. Chem.* **2015**, *71*, 3-8.
